# Development of an inhibitor of the mutagenic SOS response that suppresses the evolution of quinolone antibiotic resistance†

**DOI:** 10.1039/d4sc00995a

**Published:** 2024-05-16

**Authors:** Jacob D. Bradbury, Thomas Hodgkinson, Adam M. Thomas, Omprakash Tanwar, Gabriele La Monica, Vanessa V. Rogga, Luke J. Mackay, Emilia K. Taylor, Kiera Gilbert, Yihua Zhu, Amber Y. Sefton, Andrew M. Edwards, Charlotte J. Gray-Hammerton, Gerald R. Smith, Paul M. Roberts, Timothy R. Walsh, Thomas Lanyon-Hogg

**Affiliations:** a Department of Pharmacology, University of Oxford OX1 3QT UK thomas.lanyon-hogg@pharm.ox.ac.uk; b Ineos Oxford Institute for Antimicrobial Research, Sir William Dunn School of Pathology, University of Oxford OX1 3RE UK; c Dipartimento di Scienze e Tecnologie Biologiche, Chimiche e Farmaceutiche (STEBICEF), University of Palermo 90128 Italy; d Fred Hutchinson Cancer Center Seattle WA 98109-1024 USA; e Department of Infectious Disease, Faculty of Medicine, Imperial College London W2 1NY UK; f Chemistry Research Laboratory, Department of Chemistry, University of Oxford OX1 3TA UK

## Abstract

Antimicrobial resistance (AMR) is a growing threat to health globally, with the potential to render numerous medical procedures so dangerous as to be impractical. There is therefore an urgent need for new molecules that function through novel mechanisms of action to combat AMR. The bacterial DNA-repair and SOS-response pathways promote survival of pathogens in infection settings and also activate hypermutation and resistance mechanisms, making these pathways attractive targets for new therapeutics. Small molecules, such as IMP-1700, potentiate DNA damage and inhibit the SOS response in methicillin-resistant *S. aureus*; however, understanding of the structure–activity relationship (SAR) of this series is lacking. We report here the first comprehensive SAR study of the IMP-1700 scaffold, identifying key pharmacophoric groups and delivering the most potent analogue reported to date, OXF-077. Furthermore, we demonstrate that as a potent inhibitor of the mutagenic SOS response, OXF-077 suppresses the rate of ciprofloxacin resistance emergence in *S. aureus*. This work supports SOS-response inhibitors as a novel means to combat AMR, and delivers OXF-077 as a tool molecule for future development.

## Abstract 2

Bacterial DNA-damage repair activates the SOS response, which promotes hypermutation and antibiotic resistance. SAR investigation identified OXF-077, a small molecule DNA-repair and SOS-response inhibitor that suppresses resistance evolution.

## Graphical abstract

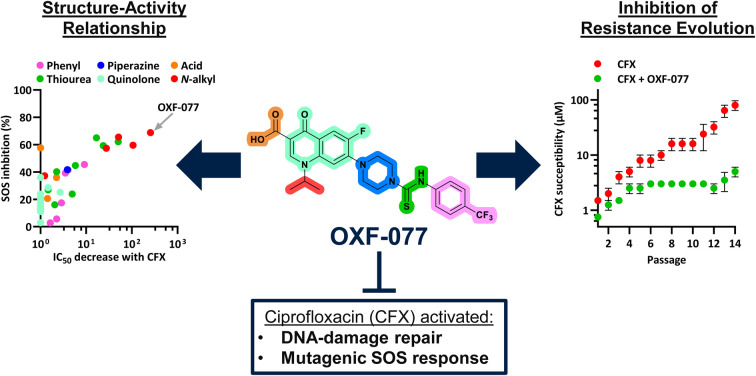

## Introduction

Antimicrobial resistance (AMR) is one of the most pressing challenges to global healthcare and is predicted to cause 10 million deaths per year by 2050.^1^ The evolution of AMR has been accelerated by improper and overuse of antibiotics in humans and agriculture,^2^ and there is an urgent need for development of new classes of antibiotics.^3^ However, even when new antibiotics have reached the market, these compounds have been rapidly compromised by the emergence of resistant strains or selection of bacteria already harbouring resistance to related antibiotics.^4^ Any new antibiotics must therefore be safeguarded as agents-of-last-resort, leading to a limited commercial market and financially disincentivising development.^5^

One aspect of addressing the AMR challenge requires development of new molecules with novel mechanisms of action (MoA). This includes not only new antimicrobial molecules but also compounds that can be partnered with existing antibiotics to block resistance mechanisms and enhance efficacy, such as inhibitors of beta-lactamases or efflux pumps.^6,7^ An emerging target for developing antibiotic adjuvants is the bacterial DNA-repair and SOS-response pathways, which control upregulation of hypermutation, horizontal gene transfer, persister cell formation, and virulence during bacterial stress.^8–13^ DNA damage in the bacterial genome can result from the oxidative burst generated by neutrophils during infection or by treatment with antibiotics such as quinolones, which induce DNA double-stranded breaks (DSBs).

**Fig. 1:**
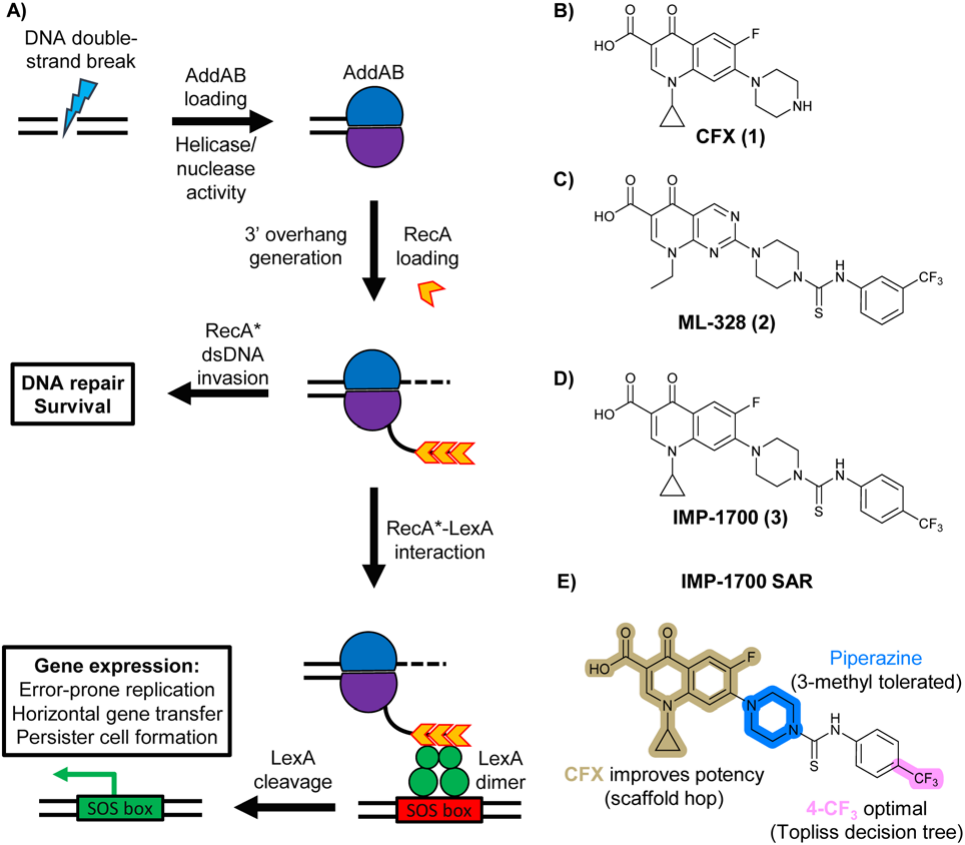
The bacterial DNA-damage repair and SOS-response pathways, and activator or inhibitor small molecules. (A) Schematic of the DNA-repair and SOS-response pathways initiated by processing of a DNA double-strand break by AddAB. (B) Structure of DNA-damaging quinolone antibiotic ciprofloxacin (CFX, 1), an inhibitor of DNA gyrase and topoisomerase IV. (C) Structure of first-generation bacterial DNA-repair inhibitor ML-328 (2).^20^ (D) Structure of MRSA DNA-repair and SOS-response inhibitor IMP-1700 (3).^21^ (E) Overview of prior structure–activity relationship (SAR) investigation of IMP-1700.^21^

In bacteria, repair of DSBs is initiated by the enzyme complex AddAB, found mainly in Gram-positive bacteria, or RecBCD, found mainly in Gram negatives.^9^ AddAB and RecBCD are ATP-dependent helicase–nucleases that function through complex biochemical mechanisms of DNA processing,^14–16^ ultimately resulting in generation of 3′ single-stranded DNA.^15^ Multiple copies of RecA protein bind to the single-stranded DNA to form a RecA-DNA filament (RecA*), which invades intact double-stranded DNA to continue homologous recombination.^17^ RecA* also binds the transcriptional repressor LexA, triggering LexA autocleavage^18^ and initiating expression of SOS box genes, such as error-prone DNA polymerase IV, LexA repressor protein, and DNA gyraseAB (Fig. 1A).^19^

AddAB and RecBCD are attractive targets for small-molecule intervention, as these complexes initiate the process leading to both homologous DNA repair and activation of the SOS response. Genetic studies in methicillin-resistant *S. aureus* (MRSA) show AddAB (also known as RexAB in *S. aureus*) is required for infection,^22^ mutagenic DNA repair^23^ and antibiotic tolerance.^24^ AddAB and RecBCD are highly conserved, with either complex present in approximately 95% of sequenced bacteria;^25^ thus, small-molecule inhibitors may have broad spectrum activity. AddAB has two nuclease domains and one helicase, whereas RecBCD has two helicases and one nuclease. Sequence homology indicates the helicase–nuclease domains of AddA correspond to RecB, whereas AddB and RecC possess similar inactivated helicase motifs, with an addition nuclease domain in AddB.^25^ Crystal structures of RecBCD^14^ and AddAB^26^ have confirmed structural similarities, although with further important differences (discussed in detail in ref. 15). Furthermore, no closely related mammalian orthologue has been identified,^25^ making RecBCD and AddAB attractive antibiotic targets as there may be limited effects on humans. Genetic knockouts have shown that loss of AddAB/RecBCD activity increases the efficacy of DNA-damaging quinolone antibiotics, such as ciprofloxacin (CFX, 1, Fig. 1B).^9,21^ Small-molecule AddAB/RecBCD inhibitors may therefore increase the efficacy of DNA-damaging antibiotics, and also potentially synergise with non-DNA damaging antibiotics since almost all antibiotics have been shown to upregulate the SOS response to promote bacterial survival and resistance.^24^ As stand-alone agents, AddAB/RecBCD inhibitors may also promote immune clearance of infections by the host immune response.^22^

There have been several reports of AddAB/RecBCD inhibitors, although development has been hindered by mammalian cytotoxicity and lack of *in vivo* efficacy.^9^ML-328 (2, Fig. 1C) was identified by high-throughput screening against *E. coli* growth in the presence of T4 *gene* 2 mutant phage, whose survival is blocked by RecBCD activity.^20^ Compound 2 inhibits purified RecBCD and AddAB with a half-maximal inhibition (IC_50_) value of 25 μM and 5 μM, respectively.^20^ A more potent inhibitor, IMP-1700 (3, Fig. 1D), was subsequently developed by substitution of the pipemidic acid substructure with a CFX substructure, based on the potential for DNA intercalation as a mechanism of inhibition, and optimisation of the aromatic thiourea substituent following the Topliss decision tree^27^ to a 4-(trifluoromethyl)phenyl group (Fig. 1E).^21^ Compound 3 sensitizes clinical MRSA to sub-lethal concentrations of CFX (9.4 μM) with nanomolar potency and also exhibits modest inhibition of the MRSA SOS response.^21^ However, despite the potentially therapeutically beneficial phenotypes produced by 3, the structure–activity relationship (SAR) of this series remains largely unexplored.

We therefore sought to investigate the SAR of 3 to understand key pharmacophoric requirements for DNA-damage potentiation and SOS inhibition. SAR exploration delivered 39 (OXF-077) as the new best-performing compound from this series, which both potentiates DNA damage and inhibits the evolution of quinolone resistance in *S. aureus*. This work demonstrates the potential of SOS-response inhibitors as a novel means to slow resistance emergence and provides OXF-077 as a valuable tool molecule for future development.

## Results and discussion

### Design and synthesis of analogues of 3

**Scheme 1:**
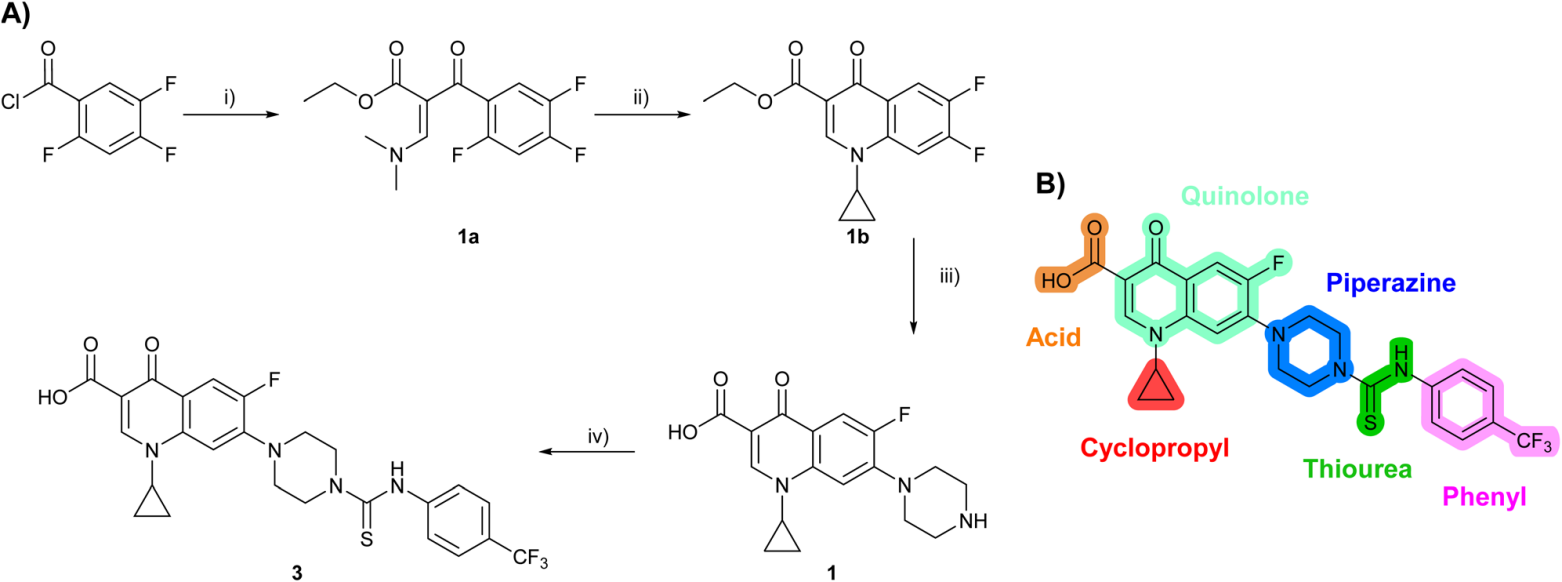
Synthesis of compounds 1 and 3. (A) Reagents, conditions and yields: (i) ethyl 3-(*N*,*N*-dimethylamino)acrylate, Et_3_N, toluene, 80 °C, 20 h (65%); (ii) (a) cyclopropylamine, Et_2_O, EtOH, RT, 3 h. (b) K_2_CO_3_, DMF, 100 °C, 18 h (58%); (iii) (a) piperazine, MeCN, 80 °C, 18 h; (b) NaOH (1 M), 80 °C, 2 h (96%); (iv) 4-(trifluoromethyl)phenyl isothiocyanate, K_2_CO_3_, MeCN, RT, 18 h (92%). (B) Overview of structure–activity relationship (SAR) investigation in this study, with points of variation of 3 colour coded as phenyl (pink), thiourea (green), piperazine (blue), quinolone (turquoise), carboxylic acid (orange), and cyclopropyl (red). Synthetic routes to specific analogues are presented in ESI Schemes S1–S5,† colour coded as in panel (B).

Building on the development of 3, further exploration was performed at sites where SAR understanding was limited, including the phenyl ring, thiourea linker, quinolone substructure, carboxylic acid and *N*-alkyl substituent (Fig. 1E). Synthetic routes were established to introduce variations at multiple positions within the scaffold of 3 (Schemes 1 and S1–S5†). Compound 3 was synthesised from ethyl 3-(*N*,*N*-dimethylamino)acrylate and 1,3,4-trifluorobenzoyl chloride, with subsequent cyclisation using cyclopropyl amine to generate the fluoroquinolone subunit 1b.^28^ S_N_Ar with piperazine and ester hydrolysis using base afforded CFX (1), which was coupled with 4-(trifluoromethyl)phenyl isothiocyanate to give 3 in a 33% yield over five steps (Scheme 1).

Substitution of the phenyl ring was previously optimised, demonstrating *para*-electron withdrawing groups (–CF_3_, –NO_2_) offered improved potency compared to the *ortho*- or *meta*-substituted phenyl.^20,21^ However, heteroaromatic and aliphatic substituents at this position were not investigated. Reaction of CFX with either commercial isothiocyanates or amines activated with 1,1′-thiocarbonyldiimidazole afforded analogues 4–7 (Table 1 and Scheme S1†). Further analogues were prepared from reaction of CFX with 1,1′-thiocarbonyl compounds to generate analogues 8–10 (Table 1 and Scheme S1†).

**Table 1:** Compound activity in JE2 MRSA. Points of variation in the structure of 3 are colour coded as in Scheme 1B; phenyl (pink), thiourea (green), piperazine (blue), quinolone (turquoise), carboxylic acid (orange), and cyclopropyl (red). Inhibition of *S. aureus* (USA300 JE2) growth IC_50_ (μM) measured with or without CFX (6.1 μM). Inhibition (%) of *S. aureus* (USA300 JE2 *precA-gfp*) SOS response activated by CFX (96 μM) at 2.5 μM compound and in dose–response for selected compounds

Compound	Scaffold	R	Compound IC_50_ (μM)	Compound + CFX IC_50_ (μM)	SOS inhibition at 2.5 μM (%)	SOS inhibition IC_50_ (μM)
2 (ML-328)	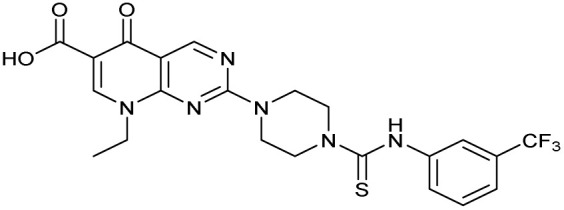	—	>10	3.7 ± 0.29	25 ± 2.0	>10
3 (IMP-1700)	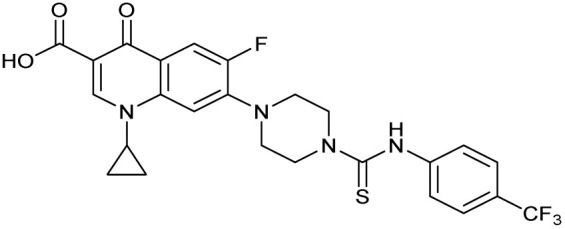	—	7.6 ± 0.14	0.071 ± 0.0018	60 ± 8.0	0.73 ± 0.087
4	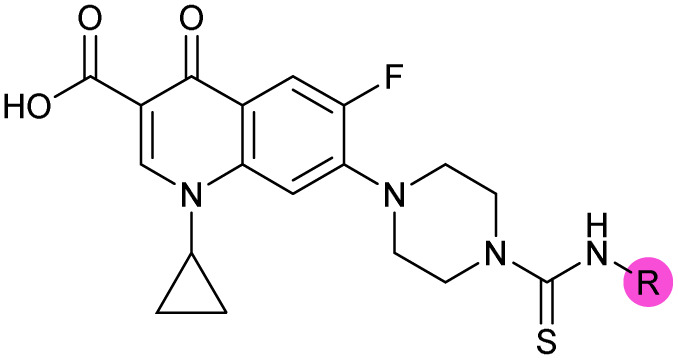	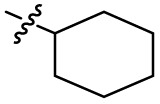	3.2 ± 0.20	0.92 ± 0.035	39 ± 4.0	ND
5		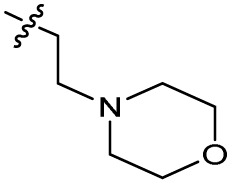	>10	6.1 ± 0.33	3.0 ± 2.0	ND
6		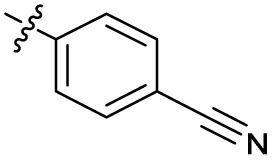	>10	2.5 ± 0.19	42 ± 2.0	ND
7		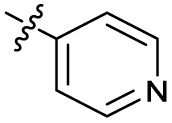	>10	>10	13 ± 1.0	ND
8	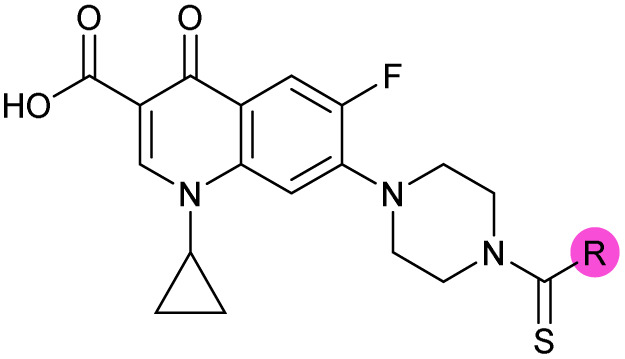	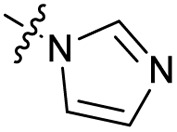	>10	3.5 ± 0.12	17 ± 7.0	ND
9		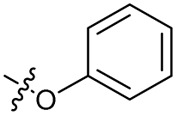	6.0 ± 0.41	0.65 ± 0.047	46 ± 8.0	ND
10		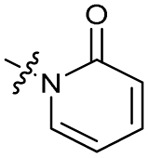	6.8 ± 0.27	3.0 ± 0.16	45 ± 9.0	ND
11	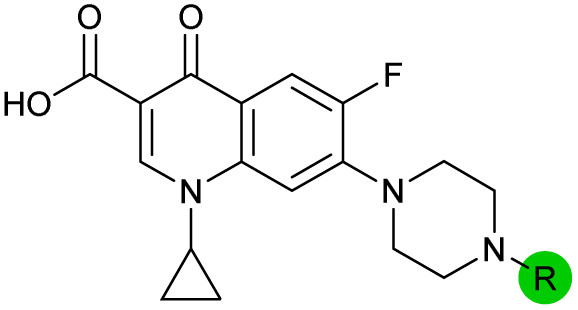	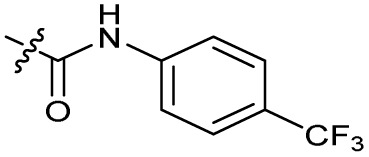	>10	0.20 ± 0.021	62 ± 6.0	2.6 ± 0.18
12		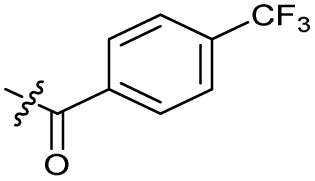	9.0 ± 0.28	1.8 ± 0.12	24 ± 2.0	ND
13		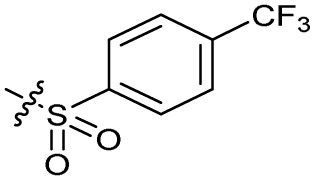	>10	4.9 ± 0.17	16 ± 4.0	ND
14		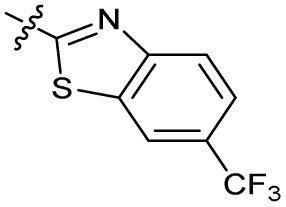	1.8 ± 0.060	1.3 ± 0.050	27 ± 4.0	ND
15	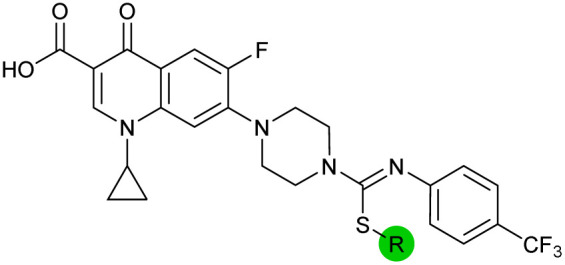	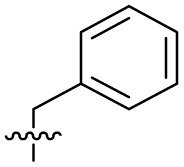	8.5 ± 0.34	0.36 ± 0.040	59 ± 9.0	ND
16		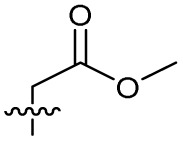	>5	2.2 ± 0.21	40 ± 2.0	ND
17		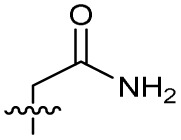	>10	1.7 ± 0.16	45 ± 5.0	ND
18	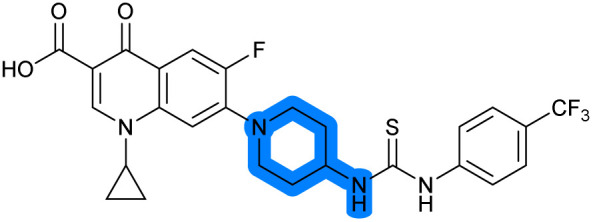	—	4.6 ± 0.090	1.2 ± 0.040	42 ± 1.0	ND
19	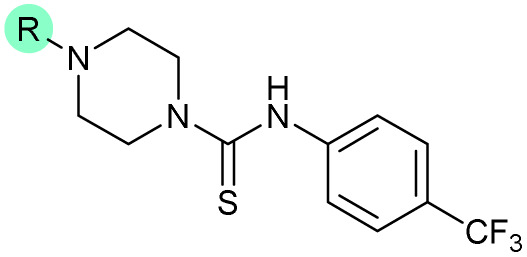	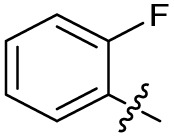	>10	>10	3.0 ± 2.0	ND
20		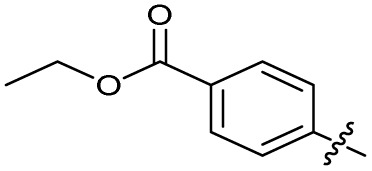	>10	>10	13 ± 9.0	ND
21		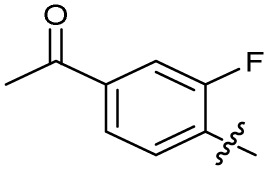	>10	>10	16 ± 4.0	ND
22		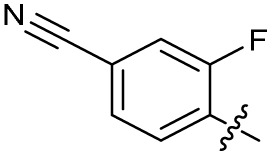	>10	6.8 ± 0.19	29 ± 7.0	ND
23		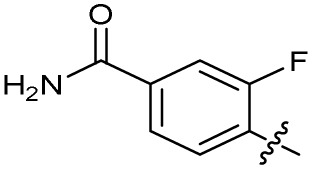	>10	>10	20 ± 9.0	ND
24		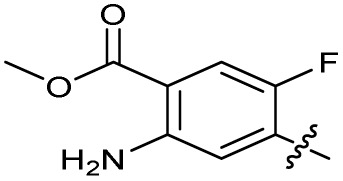	>10	>10	15 ± 4.0	ND
25		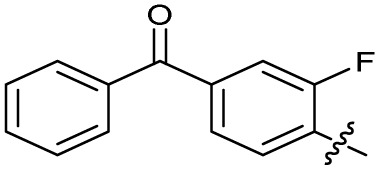	>10	>10	36 ± 6.0	ND
26		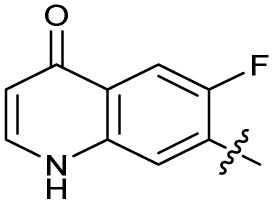	>10	>10	17 ± 6.0	ND
27		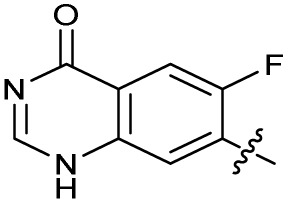	>10	>10	11 ± 3.0	ND
28		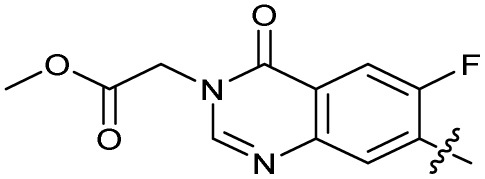	>10	>10	24 ± 11	ND
29		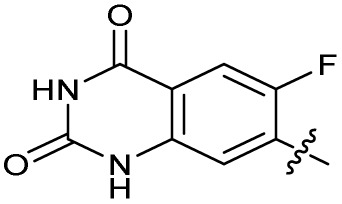	>10	>10	21 ± 18	ND
30		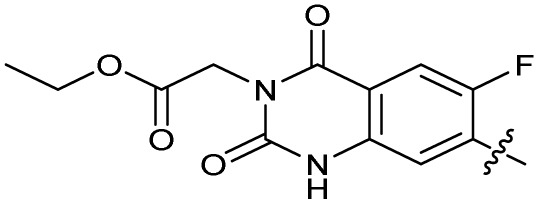	>10	>10	16 ± 3.0	ND
31		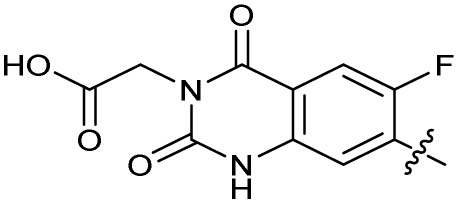	>10	>10	17 ± 3.0	ND
32		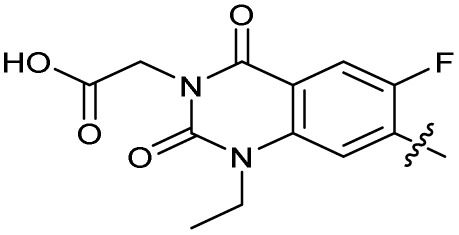	>10	>10	20 ± 2.0	ND
33	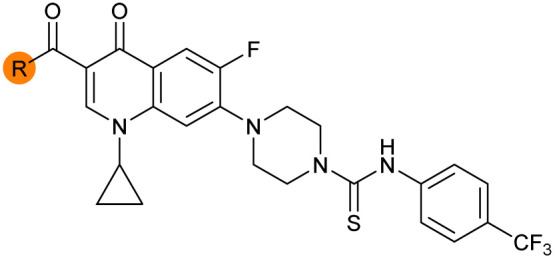	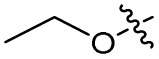	>10	>10	58 ± 4.0	0.46 ± 0.070^a^
34		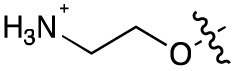	>10	4.5 ± 0.19	36 ± 2.0	ND
35		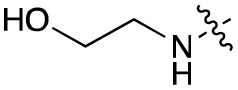	>10	7.0 ± 0.42	21 ± 3.0	ND
36	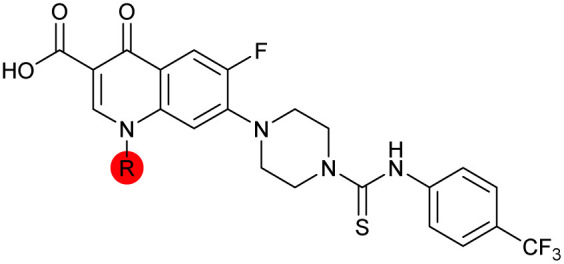	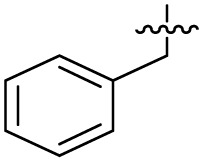	7.4 ± 0.36	6.0 ± 1.3	38 ± 6.0	ND
37		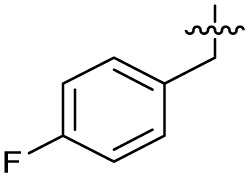	>5	0.19 ± 0.017	57 ± 3.0	ND
38		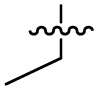	>5	0.098 ± 0.0044	66 ± 2.0	ND
39 (OXF-077)		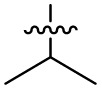	>10	0.039 ± 0.0023	69 ± 1.0	0.33 ± 0.036
40	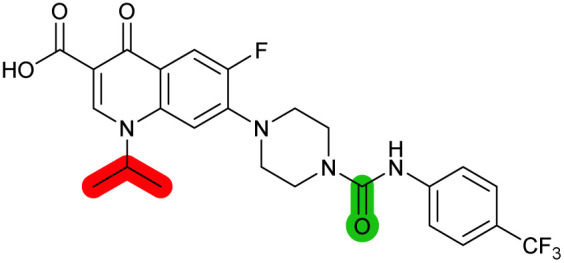	—	>10	0.56 ± 0.025	65 ± 1.0	0.71 ± 0.076

a Compound 33 exhibited a non-sigmoidal SOS inhibition dose response (Fig. S4) and an upper plateau <60% for SOS inhibition. ND = not determined. Data represent mean ± standard error of the mean (SEM, *n* = 3).

Alteration of the thiourea linker was achieved by reaction of CFX with 4-(trifluoromethyl)phenyl-substituted electrophiles to form analogues 11–13 (Table 1 and Scheme S1†). Benzothiazole analogue 14 (Table 1) was generated by reaction of 4-amino-3-iodobenzotrifluoride with 1,1′-thiocarbonyldiimidazole, followed by coupling to CFX and cyclisation (Scheme S1†). *S*-Alkylated thiourea analogues were formed by reaction of 3 with primary alkyl bromides to afford compounds 15–17 (Table 1 and Scheme S1†).

The SAR of the piperazine group has previously been investigated, showing limited tolerance for variation.^21^ An additional piperazine analogue (18, Table 1) was generated by base hydrolysis of 1b, followed by S_N_Ar with 4-*N*-Boc-aminopiperidine, and subsequent Boc deprotection followed by 4-(trifluoromethyl)phenyl thiourea formation (Scheme S2†).

The SAR of the quinolone substructure of 3 is unexplored; therefore quinolone analogues were prepared by S_N_Ar of fluorobenzyl compounds with piperazine, followed by thiourea formation as previously described to give analogues 19–25 (Table 1, Scheme S3†). Further bicyclic aromatic heterocycles were prepared from 3,4-difluoroaniline or 2-amino-4,5-difluorobenzoic acid (Scheme S3†), and used to form quinolone analogues 26–32 (Table 1).

Alteration of the carboxylate of 3 was achieved *via* intermediate 1b (Table 1); ethyl ester analogue 33 was generated by piperazine S_N_Ar and subsequent thiourea coupling (Scheme S4†). Alternatively, functionalisation of the carboxylate was achieved by hydrolysis of 1b and amide coupling with *N*-Boc-ethanolamine, followed by S_N_Ar with piperazine and subsequent thiourea formation. *N*-Boc-deprotection afforded aminoethyl ester 34, which was rearranged under basic conditions to afford hydroxyethyl amide 35 (Table 1, Scheme S4†).

To investigate the *N*-alkyl substituent of the fluoroquinolone substructure, for which no prior SAR information was available, analogues 36–39 (Table 1) were prepared by two routes. Firstly, the fluoroquinolone substructure was prepared through a Gould–Jacobs reaction of 3,4-difluoroanaline and diethyl ethoxymethylenemalonate and subsequently alkylated to generate precursors to 36 and 37 (Scheme S5†). Secondly, intermediate 1a was reacted with alkyl amines to afford precursors to 38 and 39 (Scheme S5†). Lastly, a urea analogue of compound 39 was synthesised using 4-(trifluoromethyl)phenyl isocyanate to afford compound 40 (Scheme S5†).

Collectively, a panel of 37 analogues of 3 were synthesised, with variation introduced at multiple positions in the scaffold.

### Potentiation of CFX antibacterial activity in MRSA

The prepared panel of analogues was tested in *S. aureus* JE2, a clinical MRSA strain of the USA300 lineage dominant in the USA, for potentiation of DNA damage induced by CFX. JE2 has multiple resistance elements, including a mutated DNA gyrase (Ser84Leu)^29,30^ conferring CFX resistance with a minimum inhibitory concentration (MIC) = 8 μg mL^−1^ (24 μM) (Fig. S1†). Determination of the half-maximal growth inhibition (IC_50_) of 3 at varying CFX concentrations in JE2 indicated decreased potency of 3 at lower CFX concentrations (Fig. S1†), consistent with previous reports of quantitative synergy between 3 and CFX.^21^ Growth inhibition by analogues was measured either as single agents or in combination with the highest concentration of CFX at which ≥90% bacterial growth occurred (6.1 μM), a lower CFX concentration than used in previous reports of DNA-damage potentiation by 3 (9.4 μM).^21^ To compare the activity of compounds, the fold-change in growth IC_50_ between treatment with compound alone compared to compound with CFX (ΔCFX) was calculated as a proxy measure of DNA-damage potentiation. For compounds not exhibiting toxicity at the top concentration tested (10 μM), the ΔCFX value therefore represents the lower limit of fold change in growth inhibition in the presence of CFX.

In JE2, the growth IC_50_ of 3 with CFX was 71 nM, compared to a single agent IC_50_ of 7.6 μM (Table 1, ΔCFX = 110), in-line with previous studies with higher CFX concentration.^21^ Exchange of the phenyl substituent to aliphatic groups (4, 5) resulted in a loss of CFX potentiation. In the development of 3, electron withdrawing phenyl ring substituents were found to increase CFX potentiation;^21^ however, analogues with an electron-withdrawing nitrile substituent (6), or heterocyclic substituents (7, 8, 10), showed a loss of CFX potentiation. *O*-Phenyl thiocarbamate 9 retained moderate potentiation of CFX (IC_50_ = 6.0 μM, IC_50_ + CFX = 650 nM, ΔCFX = 9.2). Collectively, this indicated the optimised 4-(trifluoromethyl)phenyl substituent of 3 afforded the highest potency of all analogues tested (Table 1).

Substitution of the thiourea linkage for a urea in compound 11 exhibited no single-agent growth inhibition (IC_50_ > 10 μM), with retention of sub-micromolar CFX potentiation (IC_50_ + CFX = 200 nM, ΔCFX ≥ 50). Exchange of the thiourea for other linkers (12–14) led to a decrease in CFX potentiation. In contrast to exchange of the thiourea, *S*-alkylation appeared to be more tolerated. Benzylated analogue 15 retained activity (IC_50_ = 8.5 μM, IC_50_ + CFX = 360 nM, ΔCFX = 24); however, *S*-acetyl analogues 16 and 17 resulted in a loss of CFX potentiation. Substitution of the piperazine linker for 4-aminopiperidine (18) exhibited moderate toxicity both with and without CFX (IC_50_ 4.6 μM, IC_50_ + CFX = 1.2 μM, ΔCFX = 3.8).

Analogues 19–32 substituting the quinolone substructure, as well as carboxylic acid analogues 33–35, demonstrated these alterations substantially decreased CFX potentiation (Table 1). In contrast, variation of the *N*-alkyl substituent of the quinolone was more broadly tolerated. Exchange of the cyclopropyl for a benzyl group (36) abolished CFX potentiation, whereas 4-fluorobenzyl analogue 37 retained activity (IC_50_ > 5.0 μM, IC_50_ + CFX = 190 nM, ΔCFX > 26). Exchange of the cyclopropyl for ethyl (38) or isopropyl (39) resulted in low growth inhibition as single agents (IC_50_ > 5.0 μM and >10 μM, respectively). Treatment with CFX resulted in IC_50_ + CFX = 99 nM (38) and 39 nM (39). This gives 38 ΔCFX = 51, and 39 ΔCFX ≥ 260, which makes 39 a more potent DNA-damage potentiator than 3 in clinical MRSA (Table 1).

Thioureas can be a point of metabolic instability in small molecules;^31^ therefore, an analogue of best-performing compound 39 was prepared, substituting the thiourea for a urea (Scheme S5†). Composite analogue 40 retained DNA-damage potentiation (IC_50_ > 10 μM, IC_50_ + CFX = 600 nM, ΔCFX > 17), consistent with prior SAR data, although DNA-damage potentiation of 40 was reduced compared to cyclopropyl-containing urea analogue 11.

Potentiation of CFX was also investigated in *E. coli* (K12 MG1655) containing a Ser83Leu *gyrA* mutation; however, compound 3 and the panel of analogues did not show activity (Fig. S2, and Table S1†). Cell penetration and efflux in Gram-negative bacteria present an additional challenge for small-molecule inhibitors and are common reasons for lack of activity in these species.^32,33^ Optimisation of Gram-negative activity therefore remains an important goal for future work.

### Compound effects on the SOS response in MRSA

**Fig. 2:**
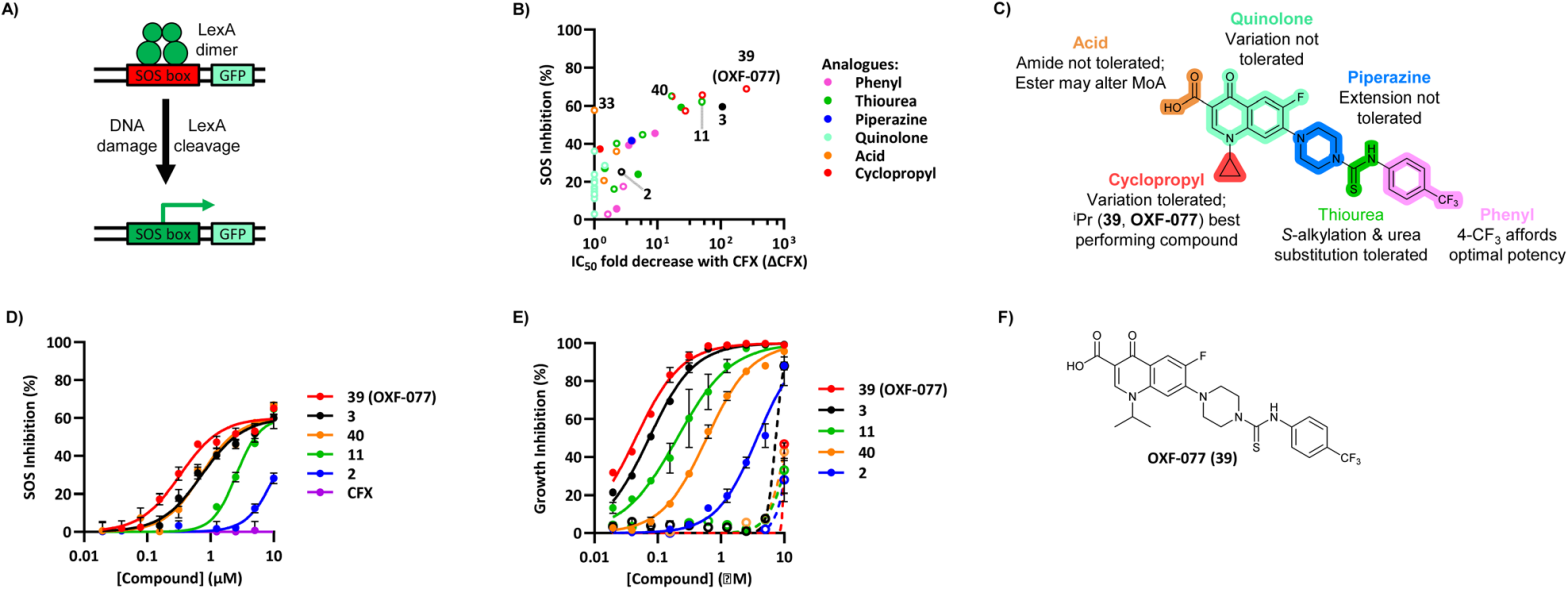
Structure–activity relationship (SAR) study of DNA-repair and SOS-response inhibitors in methicillin-resistant *S. aureus* (MRSA). (A) Schematic of MRSA JE2 SOS reporter assay; SOS response was calculated as the ratio of GFP fluorescence to OD_600_. (B) SOS response inhibition (%) by analogues at 2.5 μM compared to ΔCFX of compounds (Table 1). ● = IC_50_ values ± CFX calculated in CFX-potentiation assay; ○ = one or both IC_50_ > 10 μM in CFX-potentiation assay. Data points are colour coded corresponding to the position of variation in the structure of 3, as in Scheme 1, Table 1 and (C). Literature compounds 2 and 3 (black) are also shown. (C) Overview of SAR conclusions from this work. (D) Dose–response analysis of SOS inhibition (%) by selected compounds. Data represent mean ± SEM (*n* = 3). (E) Dose–response analysis of growth inhibition (%) by selected compounds, in the presence (solid line) or absence (dashed line) of CFX. Data represent mean ± SEM (*n* = 3). (F) Structure of best performing compound OXF-077 (39).

The compound panel was further assessed for inhibition of the SOS response in a JE2 cellular reporter assay, expressing GFP under control of the *recA* promoter, *precA-gfp* (Fig. 2A).^24^ An overnight culture of JE2-*precA-gfp* was diluted 16-fold in MHB containing CFX (96 μM) to activate the SOS response, and GFP fluorescence per OD_600_ unit (GFP/OD_600_) measured after 6 h incubation. A higher cell seeding density and CFX concentration were used in these assays compared to growth inhibition assays to increase the SOS response signal recorded. SOS inhibition (%) was initially assessed at 2.5 μM test compound, with best performing analogues progressed to dose–response analysis (Table 1). At 2.5 μM, 2 and 3 inhibited the SOS response by 25% and 60%, respectively. Phenyl analogues 5, 7, and 8 displayed <20% SOS inhibition, whereas cyclohexyl (4, 39%), 4-cyanophenyl (6, 42%), *O*-phenyl thiocarbamate (9, 46%), and 2-pyridone (10, 45%) analogues exhibited modest SOS inhibition. Thiourea analogues 12–14 showed decreased SOS inhibition; however, urea 11 (62%), thiourea benzyl adduct 15 (59%), and composite compound 40 (65%) all retained activity. Substitution of the piperazine ring for 4-aminopiperidine (18) retained a modest level of SOS inhibition (42%). Quinolone analogues showed decreased SOS inhibition, whereas variation of the cyclopropyl group was again more broadly tolerated. 4-Fluorobenzyl (37, 57%), ethyl (38, 66%), and isopropyl (39, 69%) analogues all showed similar or improved SOS inhibition compared to 3 (Table 1).

Comparison of SOS inhibition and DNA-damage potentiation (ΔCFX) indicated a statistically-significant correlation between potency in both assays (Spearman correlation *r* = 0.72, *P* < 0.0001, Fig. 2B), potentially indicative of a common mechanism or target in these effects. Matched thiourea/urea pairs (compounds 3 and 11, and compounds 39 and 40) showed increased activity with the more lipophilic thiourea analogues. However, comparison of lipophilicity (*c*Log*D*_pH 7.4_) against SOS inhibition or CFX potentiation across the analogue panel did not show increased activity with increased *c*Log*D* in either assay (Fig. S3†), suggesting lipophilicity was not a general driver of activity. Alteration of the quinolone, piperazine, and (4-trifluoromethyl)phenyl rings decreased potency in both assays, whereas analogues at the cyclopropyl and thiourea positions were tolerated and resulted in modest increases in DNA-damage potentiation and SOS inhibition. Interestingly, ethyl ester analogue 33 inhibited the SOS response (58%) but showed no growth inhibition in DNA-damage potentiation assays (ΔCFX = 1). Further investigation will be required to determine if this is a result of cellular ester hydrolysis, or if the SAR for these effects is divergent. A structural map summarising the collective findings from the SAR study is presented in Fig. 2C.

The best performing SOS inhibitors at each variation position that exhibited no single-agent growth inhibition (11, 33, 39 and 40) were further analysed in dose–response, alongside literature compounds 2 and 3. SOS inhibition reached a maximum upper plateau of ∼60% compared to no-CFX activation control; the inability to achieve complete SOS inhibition may result from partial pathway inhibition, SOS activation *via* additional pathways, or background GFP expression. Literature compounds 2 and 3 exhibited IC_50_ > 10 μM and = 730 nM, respectively (Fig. 2D). Ethyl ester analogue 33 had IC_50_ = 470 nM, although a decrease in SOS inhibition was observed above 5 μM (Fig. S4†), potentially due to limited solubility. Collectively, dose–response analysis of the selected compounds was in good agreement with CFX potentiation dose-responses (Fig. 2E).

Isopropyl analogue 39 was the most potent compound, with SOS inhibition IC_50_ = 290 nM. Urea analogue 11 possessed IC_50_ = 3.3 μM, and the composite urea and isopropyl analogue 40 exhibited IC_50_ = 900 nM. Across both assays, compound 39 was the best-performing CFX potentiator and SOS inhibitor, which we here term OXF-077 (Fig. 2F).

A recent report assessing single agent toxicity of 2 and 3 in *E. coli* using genetic methods suggests these compounds act through inhibition of DNA gyrase or topoisomerase IV, potentially resulting from the presence of quinolone substructures.^34^ Cleavage of the thiourea bond of this series under assay conditions could release the quinolone subunit and result in inhibition of gyrase/topoisomerase IV. Aqueous stability of 2, 3, 11, OXF-077 and 40 was therefore assessed in PBS, using HPLC to measure release of the corresponding quinolone subunits (Fig. S5†). Release of pipemidic acid from 2 increased over time; however, only 5% fragmentation was observed after 48 h at 37 °C. Analogues of 3 showed <2% quinolone release over 48 h, with OXF-077 displaying improved stability compared to 3 (Fig. S5†), indicating these compounds are not readily degraded in aqueous solution at 37 °C over the timeframe of biological assays. Release of quinolone subunits in intact cells, potentially as the result of enzymatic degradation, is an alternative route to generate quinolone-like phenotypes. Activation of the SOS response by 2, 3, 11, 33, OXF-077, 40 and CFX as single agents was therefore assessed in the SOS reporter assay. Analogues of 3 did not activate the SOS response at concentrations ≤10 μM (Fig. S4†), consistent with their lack of cytotoxicity as single agents ≤10 μM (Table 1). In contrast, DNA gyrase/topoisomerase IV inhibitor CFX activated the SOS response as a single agent (Fig. S4†) and did not inhibit the SOS response when already activated (Fig. 2C). Collectively, these data suggest the best performing compounds may act *via* a distinct mechanism of action in DNA-damage potentiation and SOS inhibition to canonical DNA gyrase/topoisomerase IV inhibitors. Toxicity of 2 and 3 to bacteria as single agents, rather than when used in combination with a DNA-damaging antibiotic, may be indicative of binding to ‘off-targets’ at high concentrations. In this regard, engagement of DNA gyrase/topoisomerase IV by this compound series may be possible if sufficiently high concentrations are used; however, this does not preclude binding of other biologically relevant targets, such as DNA-damage repair enzymes, at lower concentrations where compounds will act more selectively. Single-agent toxicity and a lack of CFX potentiation, for example as observed for compound 14 or 18, may be indicative of molecules acting through gyrase/topoisomerase IV inhibition. Robust determination of the target(s) and mechanism(s) of action of this series therefore remain key unanswered questions for future investigation.

### OXF-077 effect on antibiotic potency and resistance evolution

A range of antibiotics induce the SOS response in *S. aureus*, with *addB* transposon insertion mutants showing increased antibiotic sensitivity,^24^ putatively through production of ROS during antibiotic treatment. However, potentiation of other antibiotic classes by 3 has not been investigated. OXF-077, 2 and 3 (5.0 μM) were therefore tested for MIC reduction of CFX, ampicillin, cefazolin, vancomycin, gentamicin, and linezolid in JE2 MRSA (Table 2). This revealed 3 reduced the MIC of CFX and gentamicin 2-fold. OXF-077 reduced gentamicin MIC 4-fold, as well as reducing CFX and vancomycin MIC 2-fold. The decreases in antibiotic MIC are not as substantial as the decrease in OXF-077 IC_50_ with sub-lethal CFX; however, these values are in-line with quantitate drug-synergy measurements showing a 2.8-fold dose-reduction index of 3 with CFX ^21^ and 4-fold reduction of CFX MIC in *addB* transposon insertion mutants.^24^

**Table 2:** Potentiation of antibiotic activity by analogues of 3. Minimum inhibitory concentration (MIC) of antibiotics assessed against JE2 MRSA in the presence of either DMSO vehicle or analogues 2, 3, and OXF-077 (39) (5.0 μM, *n* = 3)

Antibiotic	MIC	MIC-fold reduction		
	μg mL^−1^ (μM)	2	3	OXF-077
CFX	8 (24)	1	2	2
Ampicillin	1 (2.9)	2	1	1
Cefazoline	1 (2.2)	2	1	1
Vancomycin	1 (0.7)	2	1	2
Gentamicin	1 (2.1)	2	2	4
Linezolid	2 (5.9)	1	1	1

**Fig. 3:**
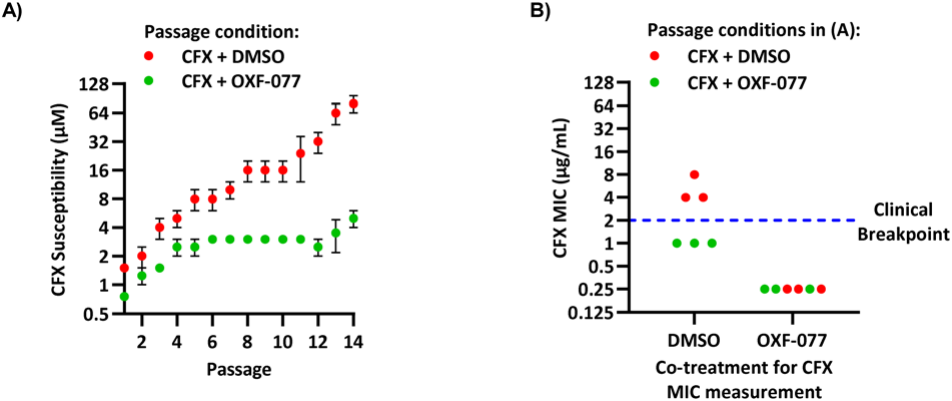
Inhibition of CFX resistance evolution with OXF-077. (A) Serial passage of MSSA with CFX in the presence of OXF-077 (5.0 μM) or DMSO vehicle, showing OXF-077 inhibited the rate of CFX resistance evolution. Data represent mean ± SEM (*n* = 3). (B) CFX MIC of strains resulting from the serial passage experiment shown in panel (A), measured using the Clinical and Laboratory Standards Institute (CLSI) method M07-A11.^35^ Either OXF-077 (5.0 μM) or DMSO was added as a co-treatment to investigate the effect of OXF-077 on cells once CFX resistance had evolved (*n* = 3). Blue dotted line indicates the EUCAST clinical breakpoint for CFX (2 μg mL^−1^).

The SOS response initiates expression of error-prone DNA polymerases which increase mutation rates and thus promote the evolution of antibiotic resistance.^19^ The effect of OXF-077 on CFX-resistance evolution was therefore investigated by serial passage of a CFX-susceptible strain of methicillin-sensitive *S. aureus* (MSSA), SH1000.^21^ SH1000 has 32-fold higher CFX susceptibility compared to the clinical JE2 isolate, with CFX MIC = 0.25 μg mL^−1^ (0.75 μM, Fig. S1†). Briefly, a CFX titration was used to determine MSSA CFX susceptibility with either OXF-077 (5.0 μM) or DMSO vehicle after overnight growth. Culture from the highest concentration of CFX at which ≥90% bacterial growth occurred was then used to inoculate the next CFX-susceptibility determination passage with either OXF-077 (5.0 μM) or DMSO vehicle; this process was repeated for 14 consecutive passages (Fig. 3A). MSSA passaged with either CFX + OXF-077 or CFX + DMSO both showed a ∼5-fold decrease in CFX susceptibility over the first five passages. After this initial decrease, the CFX susceptibility of cells passaged with CFX + OXF-077 remained at 3.0 μM. In contrast, MSSA passaged with CFX + DMSO continued to become progressively less susceptible and, after 14 passages, CFX susceptibility was 80 μM, a ∼50-fold decrease in susceptibility. The ∼5-fold decrease in CFX susceptibility observed over passages 1–5 with CFX + OXF-077 may reflect upregulation of non-mutational resistance mechanisms, such as efflux pumps. DNA sequencing of strains passaged with OXF-077 may provide further mechanistic insight into the target of this series and resistance mechanisms. The effect of OXF-077 (5.0 μM) or DMSO alone on CFX susceptibility was also determined by serial passage without CFX, which indicated CFX susceptibility remained constant for both conditions over 14 passages (Fig. S6†).

To assess the clinical relevance of suppressed CFX resistance evolution, the CFX MICs of isolates resulting from the serial passage were measured using the Clinical and Laboratory Standards Institute (CLSI) method M07-A11 ^35^ (Fig. 3B). Isolates arising from serial passage with CFX + DMSO had a CFX MIC of 4 or 8 μg mL^−1^, which is above the CFX clinical breakpoint for *S. aureus* of 2 μg mL^−1^ as defined by the European Committee on Antimicrobial Susceptibility Testing (EUCAST).^36^ In contrast, isolates resulting from serial passage with CFX + OXF-077 had a CFX MIC of 1 μg mL^−1^, remaining below the EUCAST clinical breakpoint even after serial passage with the antibiotic.^36^ The CFX MIC of isolates from the serial passage were also determined with OXF-077 co-treatment using the CLSI method (Fig. 3B). Isolates from serial passage with either CFX + OXF-077 or CFX + DMSO had a CFX MIC of 0.25 μg mL^−1^ when co-treated with OXF-077, indicating that OXF-077 reduced the CFX tolerance to below the EUCAST clinical breakpoint.^36^ The CFX MIC of these isolates when co-treated with OXF-077 was also equal to the CFX MIC of isolates that had been serially passaged with OXF-077 or DMSO alone and had not been exposed to CFX (Fig. S6†).

Collectively, these data therefore indicate that not only can OXF-077 slow the evolution of resistance against CFX, but also that OXF-077 can re-sensitize bacteria that have already acquired resistance to CFX, reducing the MIC to the level of bacteria that have not been exposed to the antibiotic. The previously noted potential for this series to engage DNA gyrase/topoisomerase IV at higher concentrations raises the interesting possibility of optimising compounds that could simultaneously induce DNA damage and also inhibit DNA repair and the SOS response. Such compounds may show decreased rates of resistance emergence compared to traditional quinolone antibiotics.

## Conclusions

AMR is one of the most serious public health threats and presents multifaceted challenges to development of new treatments, including technical difficulties and financial barriers.^1^ One aspect of addressing this challenge is the development of new molecules with novel mechanisms of action. Bacterial DNA repair inhibitors may act as antibiotic adjuvants to potentiate the efficacy of failing DNA damaging antibiotics, such as CFX, or slow emergence of resistance to new antibiotics which activate the SOS response.^9,20,21^IMP-1700 (3) was previously optimised as a potentiator of DNA-damage in MRSA; however, SAR information was limited.^21^

We report here the first comprehensive exploration of the scaffold of 3 with variation at multiple structural positions, identifying the thiourea and *N*-alkyl groups as sites tolerating modification. We further demonstrate a statistically significant correlation between DNA-damage potentiation and SOS-response inhibition across the series, suggesting a common mechanism in these effects. Compounds in this series have been proposed to inhibit AddAB in *S. aureus*,^20^ a component of both the DNA damage repair and SOS pathways, although the cellular target(s) remain to be robustly determined. The best-performing compound in this series, OXF-077, reduced the rate of CFX resistance evolution in *S. aureus* and re-sensitized *S. aureus* to a concentration of CFX below the clinical breakpoint once resistance had emerged. OXF-077 (39) is therefore a valuable tool molecule for further development, guided by the SAR presented here, to explore the potential of bacterial DNA-repair and SOS inhibitors in combatting the global challenge of AMR.

## Data availability

Synthetic procedures, compound characterisation, biological methods and supplementary data can be found in the ESI.†

## Author contributions

J. D. B., T. H., A. M. T., O. T., G. L. M., V. R., L. J. M., E. K. T., and K. G. synthesised compounds. J. D. B., A. M. T., A. Y. S., and Y. Z. performed testing. A. M. E., G. R. S., T. R. W., and T. L.-H. were responsible for funding acquisition. A. M. E., C. J. G.-H., G. R. S., P. M. R., T. R. W., and T. L.-H. supervised the research. J. D. B., A. M. T., and T. L.-H. wrote the manuscript with input from all authors.

## Conflicts of interest

There are no conflicts to declare.

## Supplementary Material

SC-015-D4SC00995A-s001
