# Supplementary material for: Development of an inhibitor of the mutagenic SOS response that suppresses the evolution of quinolone antibiotic resistance
Source: Chem Sci. 2024 May 16;15(25):9620–9. doi: 10.1039/d4sc00995a (PMC11206376; doi:10.1039/d4sc00995a)
Supplement: SC-015-D4SC00995A-s001 [file SC-015-D4SC00995A-s001.pdf]

Electronic Supplementary Material (ESI) for Chemical Science.
This journal is © The Royal Society of Chemistry 2024

Supporting Information

Development of an inhibitor of the mutagenic SOS response that suppresses the evolution of quinolone antibiotic resistance

Jacob D. Bradbury[a,b], Thomas Hodgkinson[a], Adam M. Thomas[a], Omprakash Tanwar[a], Gabriele La Monica[a,c], Vanessa V. Rogga[a], Luke J. Mackay[a], Emilia K. Taylor[a], Kiera Gilbert[a], Yihua Zhu[d], Amber Y. Sefton[e], Andrew M. Edwards[e], Charlotte J. Gray-Hammerton[b], Gerald R. Smith[d], Paul M. Roberts[f], Timothy R. Walsh[b], Thomas Lanyon-Hogg[a,*]

[a]Department of Pharmacology, University of Oxford, OX1 3QT, UK

[b]Ineos Oxford Institute for Antimicrobial Research, Sir William Dunn School of Pathology, University of Oxford, OX1 3RE, UK

[c]Dipartimento di Scienze e Tecnologie Biologiche, Chimiche e Farmaceutiche (STEBICEF), University of Palermo, 90128, Italy

[d]Fred Hutchinson Cancer Center, Seattle, WA, 98109-1024, USA

[e]Department of Infectious Disease, Faculty of Medicine, Imperial College London, W2 1NY, UK

[f]Chemistry Research Laboratory, Department of Chemistry, University of Oxford, OX1 3TA, UK

*thomas.lanyon-hogg@pharm.ox.ac.uk

Contents:

Supplementary Schemes 3

Supplementary Figures 9

Biological Methods 14

Chemical Synthesis 17

NMR Spectra of Final Compounds 56

HPLC Traces of Key Compounds 102

References 108

Supplementary Schemes

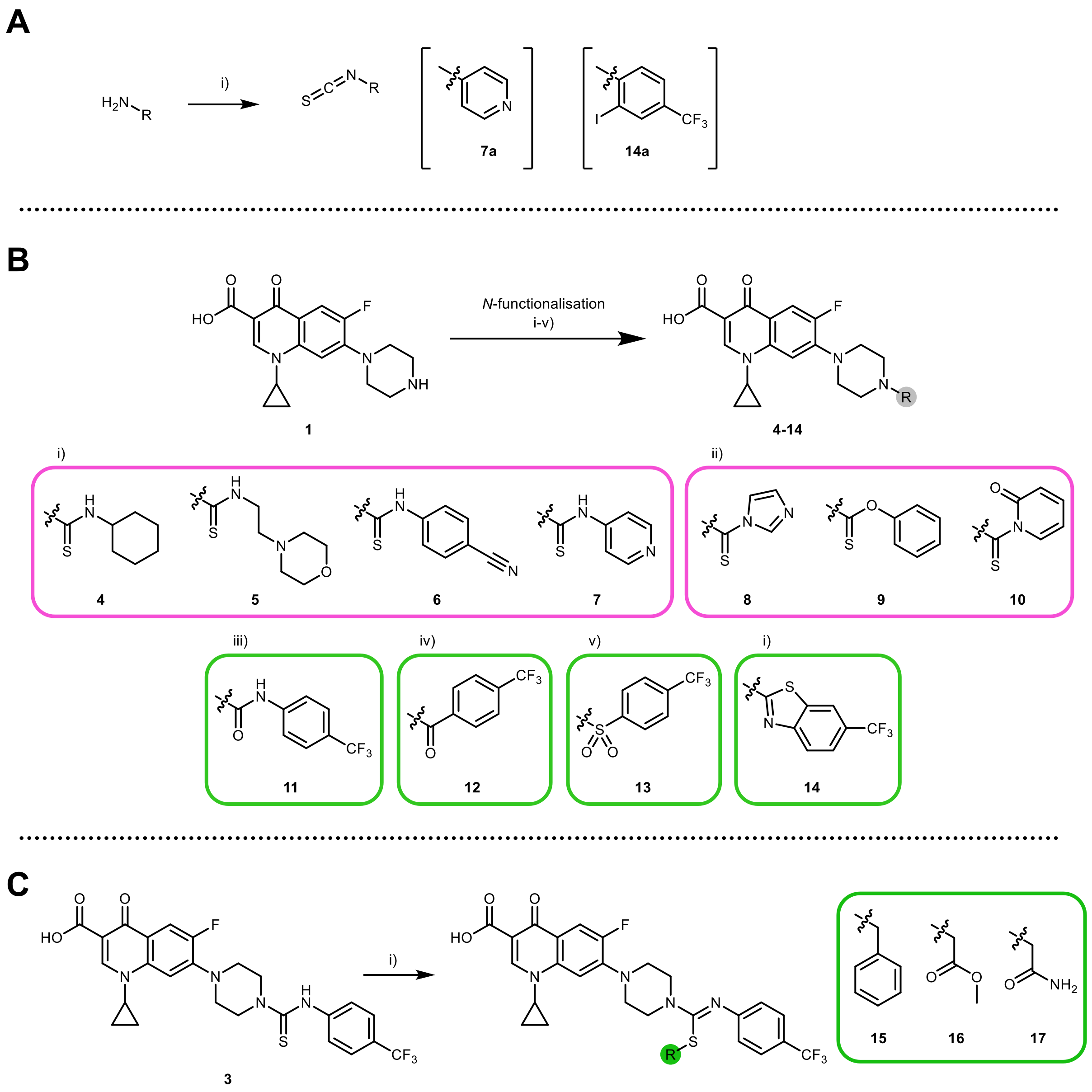

Scheme S1. Synthesis of analogues of 3 with variation at the phenyl (pink) and thiourea (green) positions. General reagents, conditions and yields: A) i) NaH, 1,1′-thiocarbonyldiimidazole, MeCN, 30 min, RT. **B**) i) R-NCS, DMF, RT, 18 h *or* R-NCS, Cs_2CO_3, MeCN, DCM, 60 °C, 18 h (4–43%); ii) respective electrophile, $NaHCO_3$, MeCN, RT, 18 h (74–84%); iii) 4-(trifluoromethyl)phenyl isocyanate, Cs_2CO_3, MeCN, DCM, 60 °C, 18 h (80%); iv) 4-(trifluoromethyl)benzoic acid, DMF, Et_3N, HATU, 0 °C to RT, 16 h (41%); v) 4-(trifluoromethyl)benzene sulfonyl chloride, Cs_2CO_3, DCM, reflux, 18 h (80%). **C**) i) R-Br, DMF, Na_2CO_3 *or* K_2CO_3, RT, 16 h (32–48%). Literature precedent indicates *S*-alkylation is favoured over the alternative *N*-alkylated regioisomer, which was supported by a ~35 ppm shift in the ^{13}C NMR thiocarbonyl peak of **16** compared to **3**.[1]

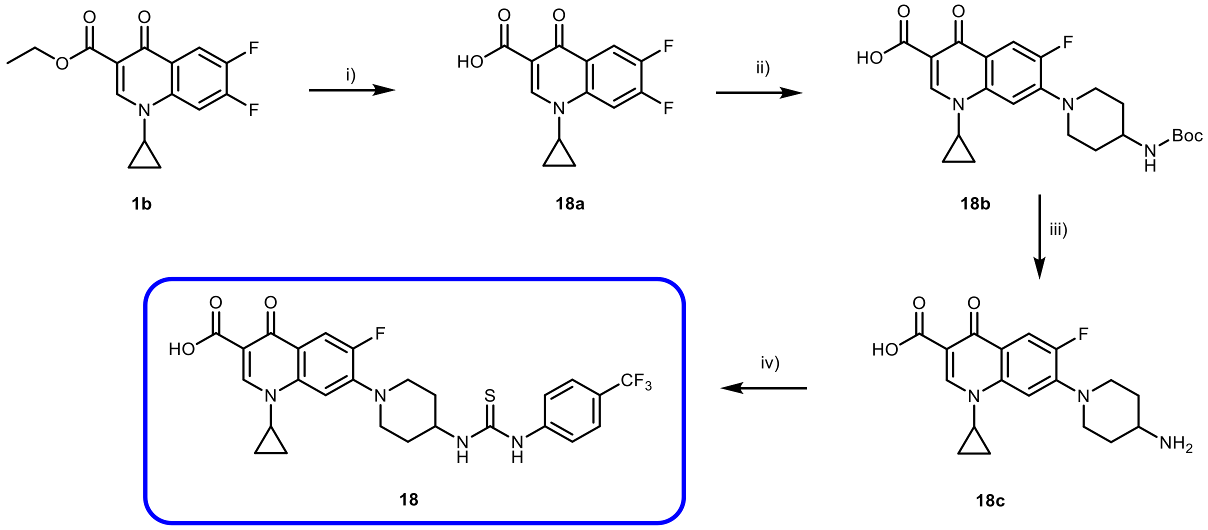

Scheme S2. Synthesis of analogue of 3 with variation at the piperazine (blue) position. Reagents, condition and yieldss: i) 1 M NaOH, THF, 60 °C, 18 h (83%); ii) 4-*N*-Boc-amino-piperidine, MeCN, 80 °C, 18 h (98%); iii) 4 M HCl in dioxane, DCM (89%); iv) 4-(trifluoromethyl)phenyl isothiocyanate, Cs_2CO_3, MeCN, DCM, 60 °C, 18 h (49%).

A

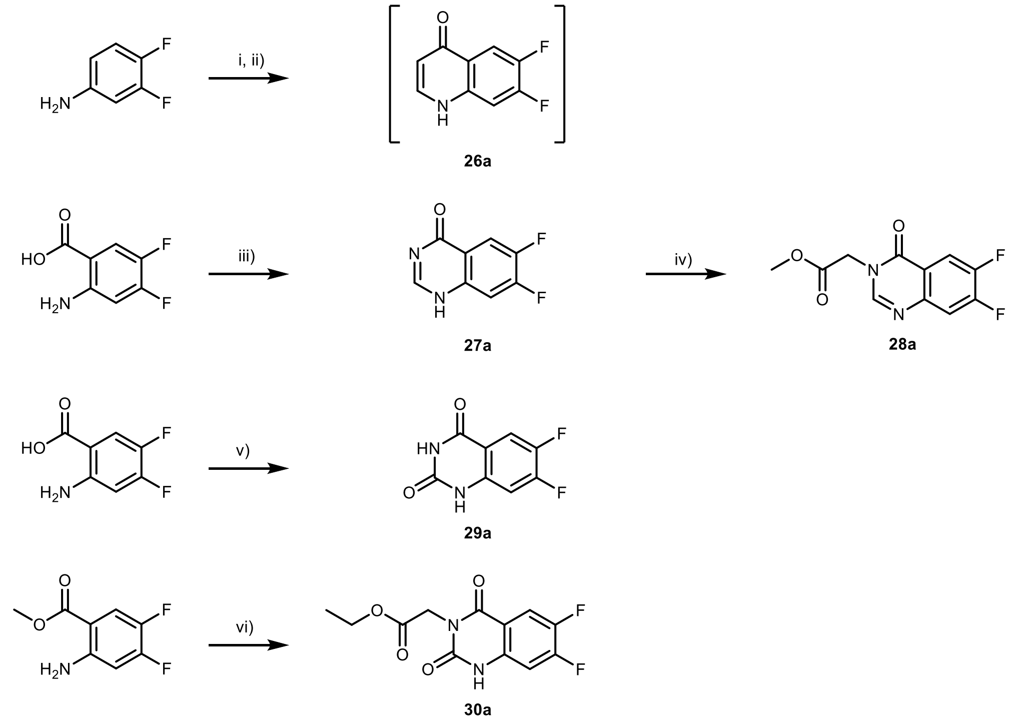
i, ii)
26a
iii)
27a
iv)
28a
v)
29a
vi)
30a

B

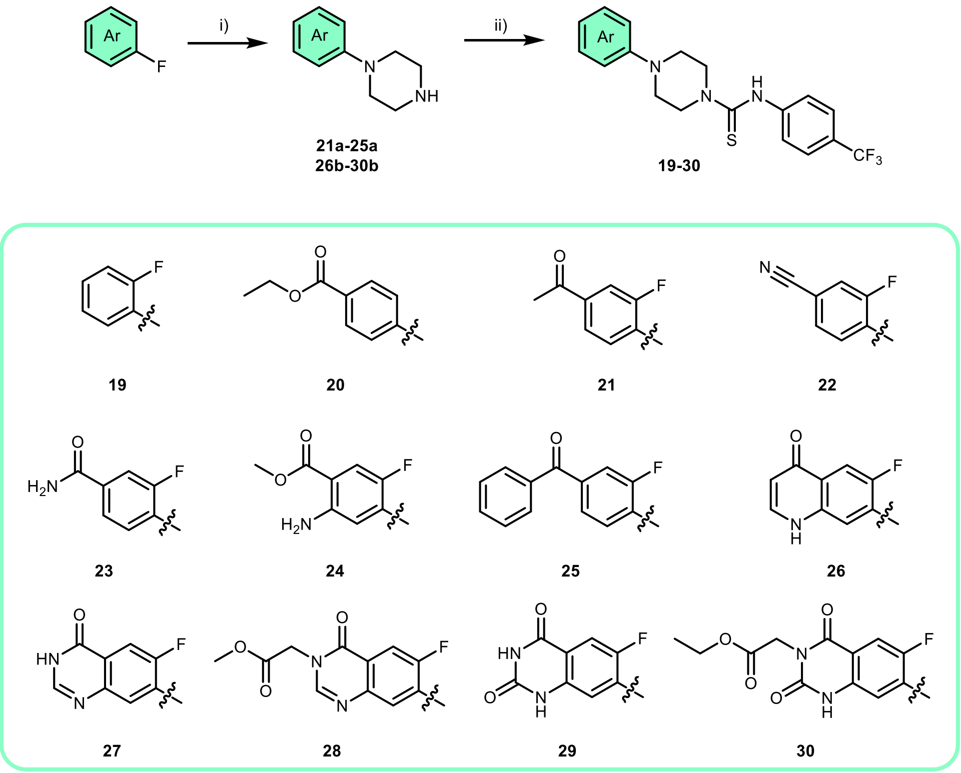
i)
ii)
21a-25a
26b-30b
19-30
19
20
21
22
23
24
25
26
27
28
29
30

C

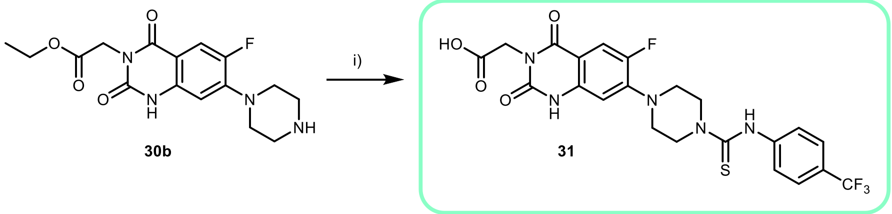

D

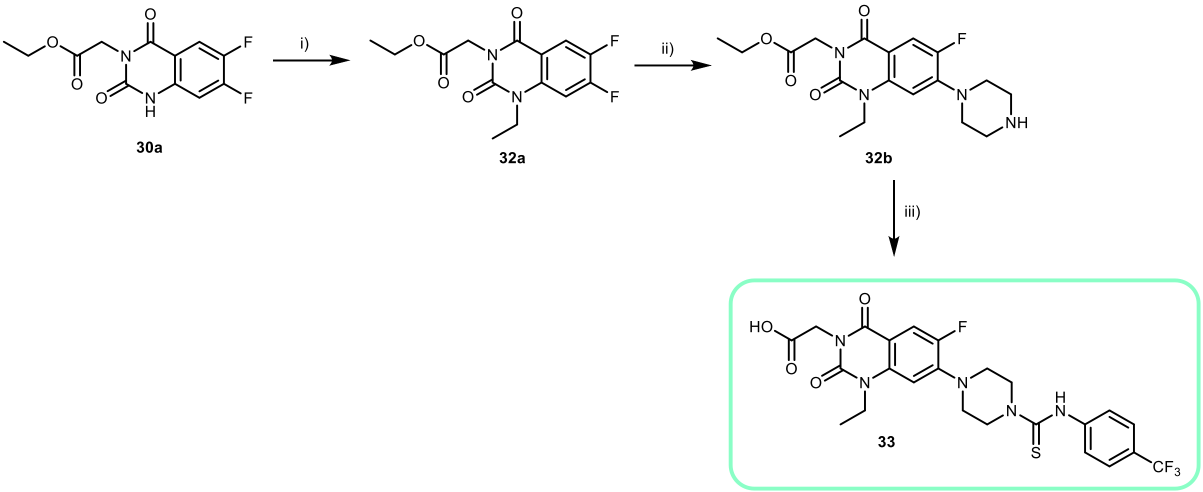

Scheme S3. Synthesis of analogues of 3 with variation at the quinolone (turquoise) position. Reagents, conditions and yields: A) i) 5-methoxymethylene-2,2-dimethyl-[1,3]-dioxane-4,6-dione, i-PrOH, 70 °C, 30 min; ii) Ph_2, Ph_2O, 220°C, 1 h; iii) AcOH, formamide, 125 °C, 24 h; iv) DMF, K_2CO_3, methyl 2-bromoacetate, 50 °C, 2 h; v) a) KOCN, H_2O, AcOH, RT, 18 h; b) NaOH, RT, 10 min; vi) a) Ethyl isocyanatoacetate, pyridine, 50 °C, 5 h; b) NaOEt, EtOH, RT, 1 h. **B**) i) Piperazine, MeCN, 80 °C, 18 h (60–96%); ii) 4-(trifluoromethyl)phenyl isothiocyanate, DMF, RT, 18 h *or* 4-(trifluoromethyl)phenyl isothiocyanate, Cs_2CO_3, MeCN, DCM, 60 °C, 18 (8–96%) h. **C**) i) a) LiOH, H_2O, RT, 30 min, b) 4-(trifluoromethyl)phenyl isothiocyanate, DMF, RT, 18 h (82%). **D**) i) EtBr, K_2CO_3, DMF, RT, 18 h (91%); ii) piperazine, MeCN, 80 °C, 18 h (96%); iii) a) LiOH, H_2O, RT, 30 min, b) 4-(trifluoromethyl)phenyl isothiocyanate, DMF, RT, 18 h (54%).

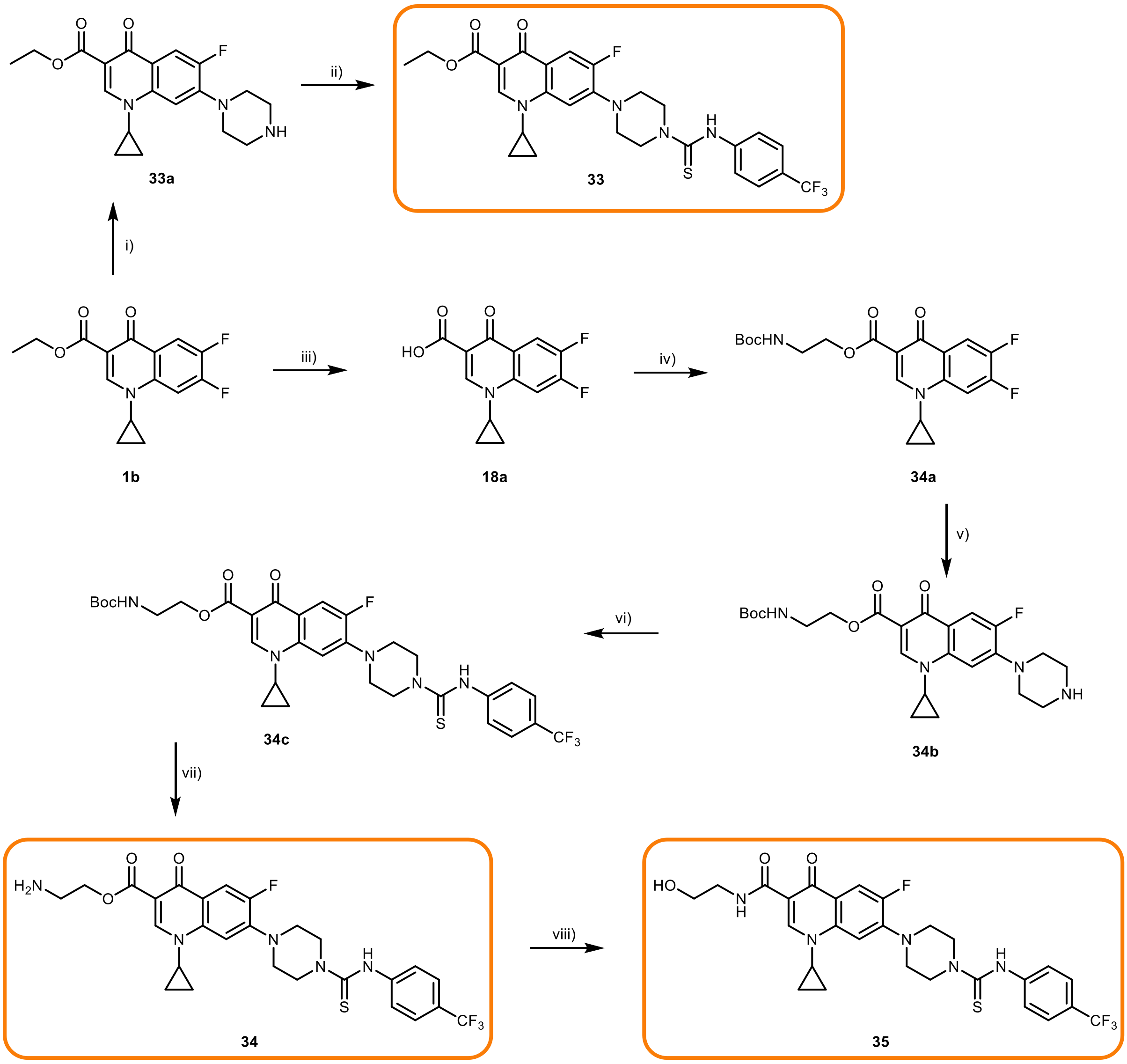

Scheme S4. Synthesis of analogues of 3 with variation at the carboxylate (orange) position. Reagents, conditions and yields: i) Piperazine, MeCN, 80 °C, 18 h (70%); ii) 4-(trifluoromethyl)phenyl isothiocyanate, DMF, RT, 18 h (54%); iii) 1 M NaOH, THF, 60 °C, 18 h (83%); iv) DIAD, PPh_3, *N*-Boc-ethanolamine, RT, 18 h (79%); v) piperazine, MeCN, 80 °C, 18 h (96%); vi) 4-(trifluoromethyl)phenyl isothiocyanate, DMF, RT, 18 h (49%); vii) 4 M HCl in dioxane, DCM, RT, 18 h (53%); viii) MeOH, Et_3N, RT, 30 min (38%).

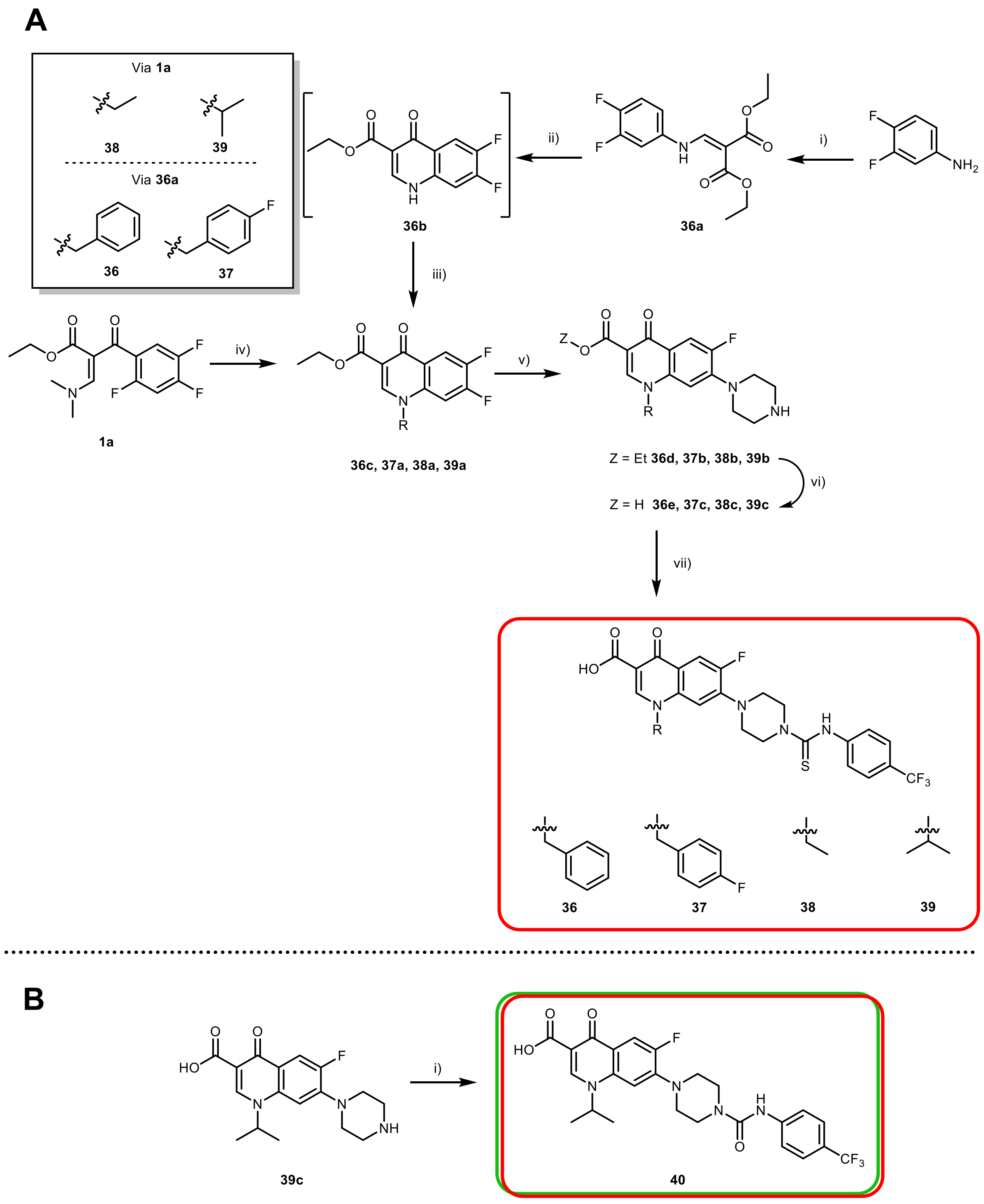

Scheme S5. Synthesis of analogues of 3 with variation at the alkyl (red) position. Reagents, conditions and yields: A) i) Diethylethyoxymethelyne malonate, 100 °C, 18 h (87%); ii) Ph_2O, reflux, 1 h; iii) RBr, K_2CO_3, DMF, RT, 16 h (76–90%); iv) a) RNH_2, Et_2O, EtOH, RT, 3 h; b) K_2CO_3, DMF, 100 °C, 18 h (48–92%); v) Piperazine, MeCN, 80 °C, 18 h (89–99%); vi) LiOH, H_2O, THF, RT, 3 h *or* 1 M NaOH, 100 °C, 18 h (69–91%); vii) 4-(trifluoromethyl)phenyl isothiocyanate, DMF, RT, 18 h (11–48%). **B**) i) 4-(trifluoromethyl)phenyl isocyanate, Cs_2CO_3, MeCN, DCM, 60 °C, 18 h (62%).

Supplementary Figures

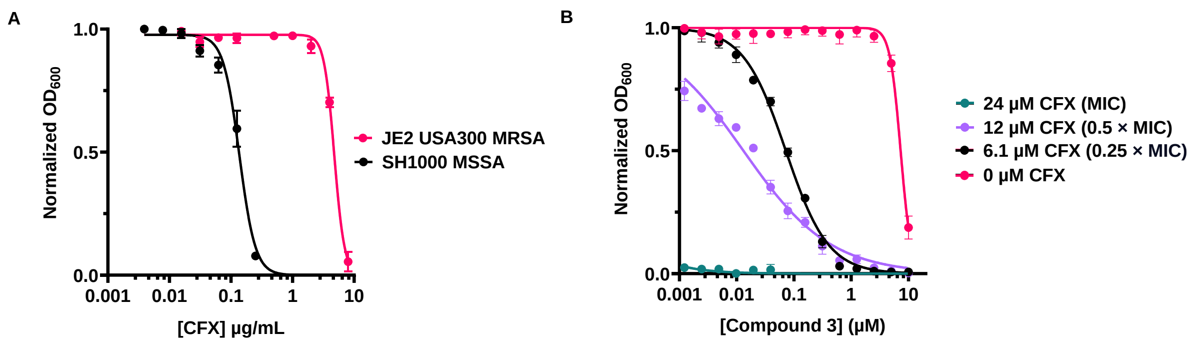

Figure S1. Growth inhibition of *S. aureus* strains with CFX and 3. A) **CFX** minimum inhibitory concentration (MIC) against *S. aureus* JE2 and SH1000 strains used in this study, demonstrating **CFX** MIC = 8 µg/mL (24 µM) for JE2 and 0.25 µg/mL (0.75 µM) for SH1000. **B)** Growth inhibition of JE2 with titration of **3** and co-treatment with fixed concentrations of **CFX** at MIC or sub-MIC levels. Data normalised to no **CFX** treatment and represent mean ± standard error of the mean (SEM, n=3).

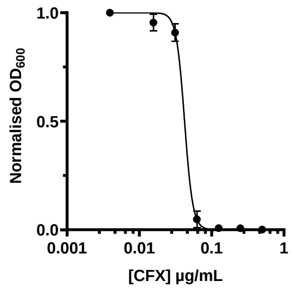

Figure S2. Growth inhibition of *E. coli* (MG1655 K12 S83L-*gyrA*) by CFX. Data normalised to no **CFX** treatment and represent mean ± SEM (n=3).

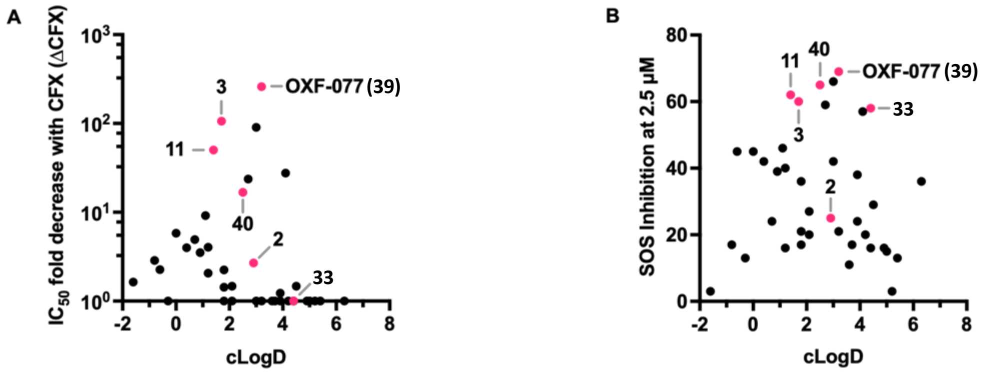

Figure S3. Comparison of cLogD$_{pH\ 7.4}$ with (A) IC_{50} fold decrease with CFX (ΔCFX) and (B) SOS inhibition (%) at 2.5 µM. cLogD$_{pH\ 7.4}$ values were calculated by CDD vault.

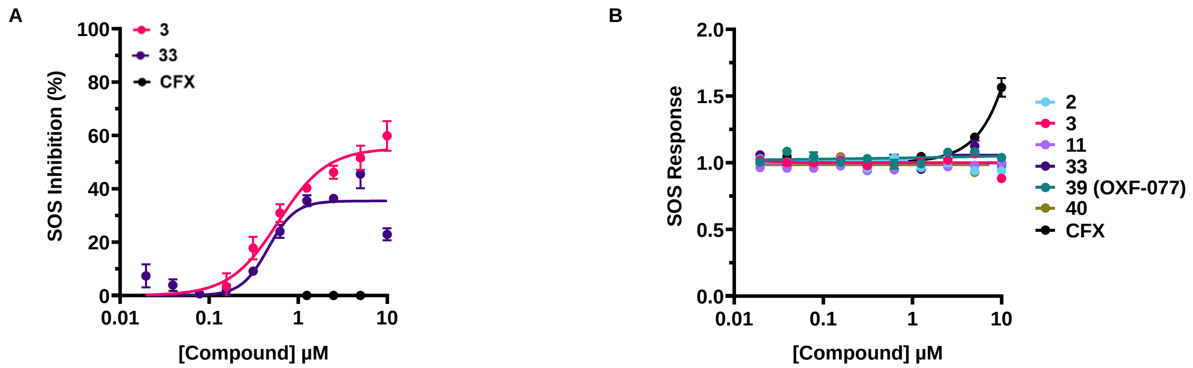

Figure S4. Dose-response analysis of compounds in *precA-gfp* JE2 MRSA SOS reporter assay. A) Inhibition of the SOS response, activated with **CFX** (96 µM), by compound **33** compared to **3** and **CFX**. Data normalised to DMSO control and represent mean ± SEM (n=3). **B)** Measurement of the SOS response when compounds **2**, **3**, **11**, **33**, **OXF-077** (**39**) and **40** are dosed as single agents, without **CFX** activation; SOS response activator **CFX** was also dosed as a single agent as a positive control for SOS activation. Data normalised to no **CFX** control and represent mean ± SEM (n=3).

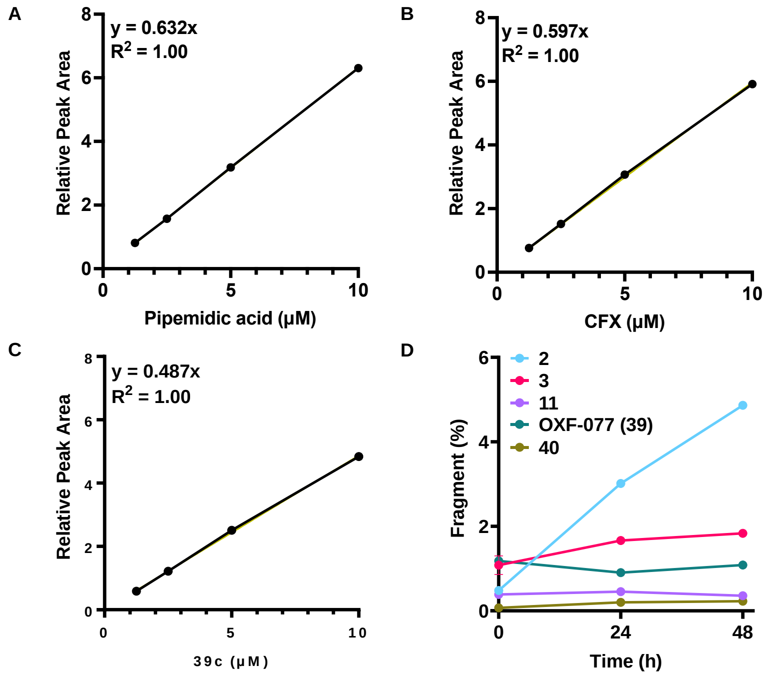

Figure S5. Aqueous stability of compounds. Calibration curves generated for **A) pipemidic acid**, **B) CFX**, and **C) 39c**, measured by HPLC. **D)** Aqueous stability of compounds **2**, **3**, **11**, **OXF-077** (**39**) and **40** (100 µM) in PBS at 37 °C, as determined by HPLC analysis. Data represent mean ± SD (n=3), error bars are present but obscured by data points.

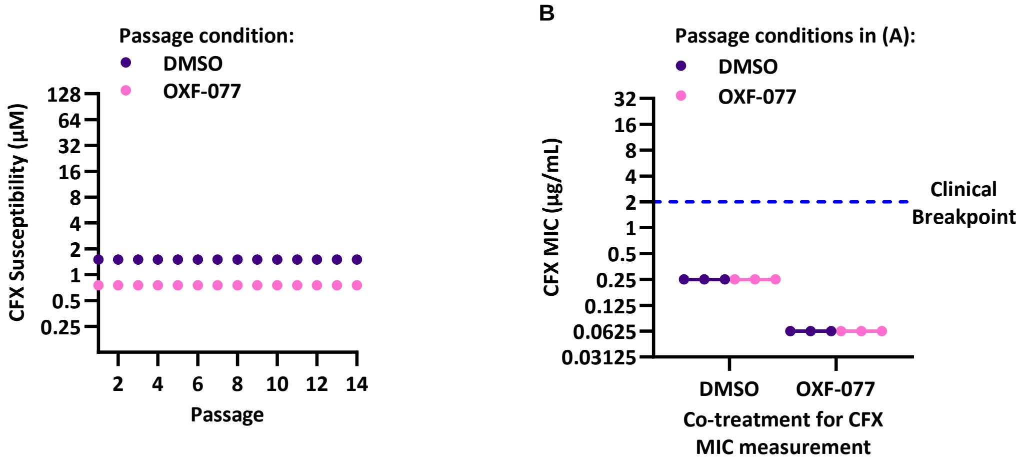

Figure S6: Lack of CFX resistance evolution with OXF-077 treatment as a single agent. **A**) **CFX**-susceptibility determined during serial passage of MSSA with **OXF-077** (5.0 µM) or DMSO alone, without **CFX**. Data represent mean ± SEM (n=3). **B**) **CFX** MIC of strains resulting from the serial passage experiment shown in panel (A), measured using the Clinical and Laboratory Standards Institute (CLSI) method, in the presence of **OXF-077** (5.0 µM) or DMSO (n=3). Blue dotted line indicates the EUCAST clinical breakpoint for **CFX** (2 µg/mL).

Table S1. Compound activity in *E. coli* (MG1655 K12 S83L-*gyrA*). Points of variation in the structure of **3** are colour coded as in Figure 1; phenyl (pink), thiourea (green), piperazine (blue), quinolone (turquoise), carboxylic acid (orange), and cyclopropyl (red). The MIC of **CFX** in *E. coli* (S83L-*gyrA* MG1655 K12) was 0.38 µM, and the first full growth concentration of **CFX** (0.19 µM, Figure S2). ND = not determined, data represent mean ± SEM (n=3).

Compound	Scaffold	R	Compound IC_{50} (µM)	Compound + CFX IC_{50} (µM)
**2 (ML-328)**	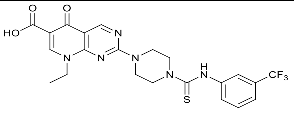	-	ND	ND
**3 (IMP-1700)**	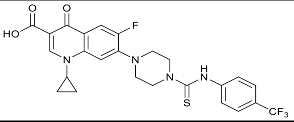	-	>10	4.8 ± 0.19
**4**	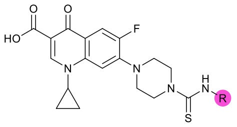	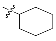	>10	>10
**5**		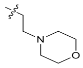	>10	>10
**6**		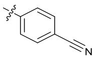	>10	8.1 ± 0.55
**7**		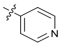	>10	5.7 ± .034
**8**	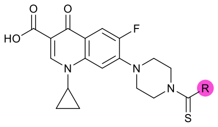	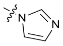	>10	>10
**9**		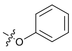	>10	>10
**10**		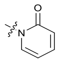	>10	6.9 ± 0.40
**11**	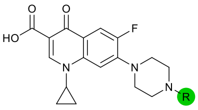	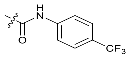	>10	8.5 ± 0.86
**12**		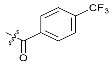	>10	>10
**13**		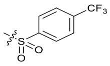	>10	>10
**14**		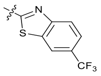	>10	>10
**15**	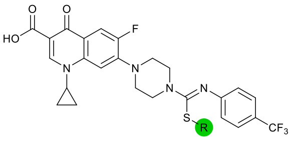	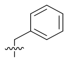	>10	>10
**16**		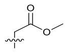	>10	6.2 ± 0.70
**17**		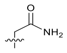	3.1 ± 0.16	0.78 ± 0.023
**18**	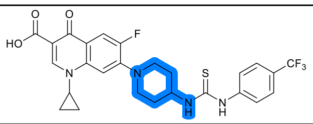	-	>10	>10
**19**	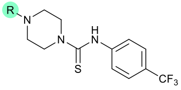	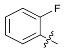	>10	>10
**20**		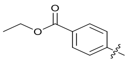	>10	>10
**21**		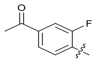	>10	>10

**22**		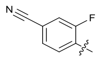	>10	>10
**23**		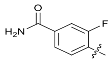	>10	>10
**24**		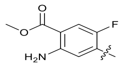	>10	>10
**25**		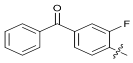	>10	>10
**26**	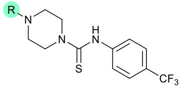	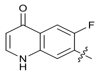	>10	>10
**27**		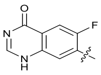	>10	>10
**28**		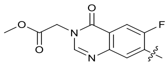	>10	>10
**29**		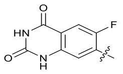	>10	>10
**30**		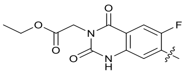	>10	>10
**31**		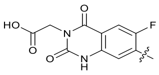	>10	3.2 ± 0.22
**32**		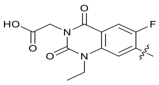	ND	ND
**33**		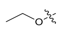	>10	>10
**34**	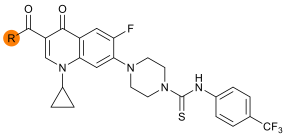	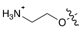	>10	>10
**35**		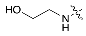	>10	>10
**36**		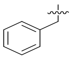	>10	>10
**37**	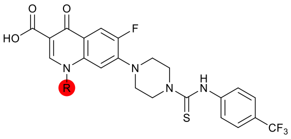	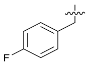	>10	>10
**38**		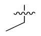	>10	3.1 ± 0.22
**39 (OXF-077)**		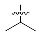	>10	>10
**40**	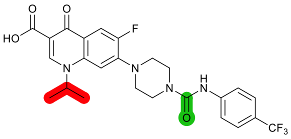	-	ND	ND

Biological Methods

Bacterial strains, culture conditions and compound treatment

Bacterial strains outlined in Table S2 were revived from a frozen stock, as an overnight culture grown on non-selective Muller Hinton Agar (MHA, Sigma-Aldrich, UK) supplemented with associated antibiotics at 37 °C for 17 h. Overnight cultures were grown in Muller Hinton Broth (MHB, Sigma-Aldrich, UK), supplemented with associated antibiotics, to late stationary phase at 37 °C and 180 RPM in a shaking incubator (SI-200, Cole Parmer, UK). Plates were incubated at 37 °C and 180 RPM in a shaking incubator (SI-200, Cole Parmer, UK) with a BreatheEasy seal (Diversified Biotech, USA). Compounds were prepared as DMSO stocks for biological experiments, except **CFX** which was dissolved in 1 µM HCl. All compounds were stored at -20 °C and thawed on the day of use.

Table S2. Bacterial Strains used in this work.

Bacterial Strain	Description	Resistance markers (Concentration)	Reference
Staphylococcus aureus USA300 JE2	Derivative of CA-MRSA USA300 LAC, cured of plasmids	-	Fey *et al.* 2013[2]
Staphylococcus aureus USA300 JE2 pCN34 *pRecA-GFP*	JE2 containing pCN34 with *gfp* under the control of the *recA* promoter	Kanamycin (155 µM)	Clarke *et al.* 2019[3]
Staphylococcus aureus SH1000	*rsbU*⁺ derivative of the laboratory strain 8325-4	-	Horsburgh *et al.* 2002[4]
Escherichia coli MG1655 K12 *S83L-gyrA*	*S83L-gyrA* derivative of the laboratory strain MG1655 K12.	-	Orritt *et al.* 2022[5]

Software analysis and plate reading

OD_{600} and fluorescence intensity readings were recorded in a CLARIOstar Plus microplate reader (BMG, UK). Measurements were background corrected against a non-inoculum control of MHB and normalised to DMSO control. GraphPad Prism 10.0.0 (131) (Dotmatics, USA) was used to generate log dose-response curves and calculate mean IC_{50} value and SEM.

Minimum Inhibitory Concentration Assay

Antibiotic susceptibility testing was determined in 384-well plates (Greiner Bio-One, UK) by MIC broth microdilution according to CLSI methods M07-A11.[6] Two-fold serial dilutions of the compounds in triplicate were performed in MHB using a Biomek i7 liquid handling platform (Beckman Coulter, USA) with a final volume of 25 µL and a no compound growth control. A direct colony suspension was made by dispersing singular, well isolated bacterial colonies from the overnight revive plates in 3 mL sterile Phosphate-Buffered Saline to achieve a turbidity of 0.5 McFarland standard (Oxoid, UK), approximately 10^8 CFU/ml. The inoculum was vortexed and then further diluted 1:100 in MHB to

achieve a final inoculum of 10^6 CFU/mL. Inoculum (25 µL) containing a fixed concentration of a second compound (or DMSO) was added to each well to achieve a final CFU/mL of 5 × 10^5, excluding no-inoculum sterility control which had only MHB added (final volume 50 µL, 1% (*v/v*) DMSO). Plates were incubated overnight for 18 h after which the OD_{600} was recorded.

SOS Reporter Assay

SOS response activation and inhibition was determined in 384-well plates (Greiner Bio-One, UK) using an SOS reporter strain (pCN34 *pRecA-GFP* USA300 JE2). Compounds in DMSO (serially diluted using a standard broth microdilution protocol where stated) were added to 25 µL of MHB in triplicate using a Biomek i7 liquid handling platform (Beckman Coulter, USA). Overnight cultures of pCN34 *pRecA-GFP* USA300 JE2 were diluted 8-fold and supplemented with **CFX** (where stated), and 25 µL added to each well (excluding no-inoculum control) to achieve 4 × 10^7 CFU (final volume 50 µL, 1% (*v/v*) DMSO). Plates were incubated for 6 h after which GFP fluorescence (Ex 375, Em 425) and OD_{600} were measured, and background corrected GFP/OD_{600} reported.

Aqueous Stability

Compounds (100 µM) were incubated at 37 °C in PBS spiked with methyl *p*-tolyl sulfone (1 mM). Samples (90 µL) were taken at 0, 24, and 48 h and were flash frozen. Samples were thawed prior to analysis. HPLC analysis was conducted using an SPD-20A UV detector (Shimadzu) set to 280 nm and an ACE Equivalence 3, C18, 150 × 4.6 mm column (Avantor).

To quantify fragmentation, calibration curves for the corresponding fragments were prepared using a four-point, 2-fold, serial dilution in PBS spiked with methyl *p*-tolyl sulfone (1 mM). The peak areas were then normalised to the methyl *p*-tolyl sulfone peak area and plotted against concentration. Samples were then analysed, and the corresponding fragment peak area was normalised to the methyl *p*-tolyl sulfone peak area, and then quantified using the corresponding calibration curve.

Serial Passage for CFX Susceptibility

Serial passage was performed in 96-well plates (Corning, UK) with **CFX** serially diluted using a standard broth microdilution protocol and either **OXF-077** (**39**) (5 µM) or DMSO (0.5% *v/v*) (99 µL). An overnight culture of SH1000 was diluted 2-fold and 1 µL used to inoculate each well (final volume 100 µL). Plates were incubated for 24 h after which the OD_{600} was recorded. The first full-growth concentration well of each isolate in triplicate was diluted 2-fold and 1 µL used to inoculate a new 96-well plate with the

same 2-fold serial dilution of **CFX** and either **OXF-077** (**39**) (5 µM) or DMSO (0.5% *v/v*) as previously described. Controls without **CFX** were also serially passaged using an identical method, and the **CFX** susceptibility recorded after each passage. This process was repeated for 14 consecutive days. **CFX** susceptibility was recorded as the first full-growth concentration.

Chemical Synthesis

General Information

Materials were purchased from commercial suppliers and used as received. Analytical thin-layer chromatography (TLC) was performed on 0.25 mm silica gel 60 F254 pre-coated plates 0.25 mm (Merck, UK) and visualized under ultraviolet light (254 and 365 nm). Purification by column chromatography was carried out using a CombiFlash R_f automated column system with RediSep silver disposable flash columns (Teledyne, USA).

^1H nuclear magnetic resonance (NMR) and ^{13}C NMR spectra were recorded at room temperature at 400 MHz and 101 MHz respectively (Bruker, USA). Chemical shifts are reported as parts per million (δ) using trimethylsilane (TMS) and the peak of the residual solvent proton signals as internal reference. Coupling constants (*J*) are reported in hertz (Hz) and averaged for interacting protons. Low-resolution mass spectroscopy (LRMS) and high-resolution mass spectroscopy (HRMS) was collected on a BioAccord (Waters, USA). HPLC analysis was conducted using an SPD-20A UV detector (Shimadzu) with 254 nm and 280 nm detection, and an ACE Equivalence 3, C18, 150 × 4.6 mm column (Avantor).

Synthetic procedures

General Procedure A: Isothiocyanate formation

The respective amine (1.0 eq) and NaH (60% suspension in mineral oil, 2.0 eq) were added to a solution of 1,1′-thiocarbonyldiimidazole (1.0 eq) in MeCN. The reaction was stirred at RT for 30 min. NH_4Cl (20 mL) was added and the product extracted with DCM (3 × 30 mL), washed with H_2O (30 mL), dried, and the solvent removed *in vacuo*. There were no further purification steps.

General Procedure B: Isothiocyanate coupling in DMF

The respective amine (1.0 eq) was added to a solution of isothiocyanate (1.5 eq) in anhydrous DMF. The reaction was stirred at RT for 18 h. The solvent was removed *in vacuo*, and the residue purified by flash silica column chromatography.

General Procedure C: Isothiocyanate coupling with Cs_2CO_3

The respective amine (1.0 eq) was added to a solution of isothiocyanate (1.3 eq), and Cs_2CO_3 (1.0 eq) in 1:1 anhydrous MeCN/DCM. The reaction was stirred at 60 °C for 18 h. NH_4Cl (20 mL) was added, the product extracted with DCM (3 × 30 mL), washed with H_2O (30 mL), dried, and the solvent removed *in vacuo*. The residue was purified by flash silica column chromatography.

General Procedure D: Boc deprotection

Boc-protected compound (1.0 eq) was added to 4 M HCl in dioxane (5 mL) and DCM (5 mL). The reaction was stirred overnight and then the solvent removed *in vacuo*. The residue was dissolved in 1:1 DCM/MeOH (20 mL) and added to an Isolute® SCX column. The column was washed with DCM (10 mL). The free amine was eluted for the column using 7 N NH_3 in MeOH (20 mL). The solvent was removed *in vacuo*, and the residue washed with DCM (5 mL).

General Procedure E: S_NAR Amine coupling

The respective amine (4.0 eq) was added to the required difluoro heterocyclic compound (1.0 eq) in anhydrous MeCN. The reaction mixture was refluxed at 80 °C for 18 h. The solvent was removed *in vacuo* and the residue was purified via flash silica column chromatography.

General procedure F: Quinolone *N*-modification and intramolecular cyclisation

The respective amine (2.0 eq) was added to **1a** (1.0 eq) in 2:1 Et_2O/EtOH. The reaction was stirred at RT for 3 h. The solvent was removed *in vacuo* and the residue dissolved in DMF, followed by the addition of K_2CO_3 (4.0 eq) and heating at 100 °C for 18 h. The solvent was removed *in vacuo*, and the residue purified by flash silica column chromatography.

Ethyl (*Z*)-3-(dimethylamino)-2-(2,4,5-trifluorobenzoyl)acrylate (1a)

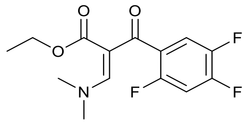

2,4,5-Trifluorobenzoyle chloride (1.1 mL, 7.7 mmol, 1.5 eq) was added to a solution of ethyl-3-(diethylamino)acrylate (1.0 g, 5.1 mmol, 1.0 eq) and Et_3N (2.1 mL, 15 mmol, 3.0 eq) in anhydrous toluene (20 mL). The reaction was heated at 80 °C for 20 h. The reaction mixture was then cooled, quenched with H_2O (30 mL), and extracted with EtOAc (3 × 30 mL). The organic extracts were combined, washed with H_2O (30 mL) and brine (30 mL), dried over Na_2SO_4, filtered, and concentrated *in vacuo*. Purification by flash silica column chromatography (5–100% EtOAc/petroleum ether) gave **1a** as a yellow oil (1.0 g, 65%). R_f = 0.15 (SiO_2; Petroleum ether:EtOAc, 60:40); **^{1}H NMR** (400 MHz, $CDCl_3$) δ: 7.78 (s, 1H), 7.51–7.40 (m, 1H), 6.87 (ddd, *J* = 15.9, 9.8, 6.2 Hz, 1H), 4.02 (q, *J* = 7.1 Hz, 2H), 3.31 (br s, 3H), 2.87 (br s, 3H), 1.01 (t, *J* = 7.1 Hz, 3H); **LRMS** *m/z* (ESI^+) 302 ($[M+H]^+$). These data are in agreement with the literature.[7]

Ethyl 1-cyclopropyl-6,7-difluoro-4-oxo-1,4-dihydroquinoline-3-carboxylate (1b)

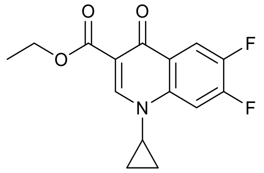

Prepared following *general procedure F from* **1a** (220 mg, 0.70 mmol) and cyclopropyl amine (0.97 mL, 1.40 mmol). Purification by flash silica column chromatography (60–75% EtOAc/petroleum ether) gave **1b** as white solid (120 mg, 58%). R_f = 0.70 (SiO_2; Petroleum ether:EtOAc, 50:50); **^{1}H NMR** (400 MHz, $CDCl_3$) δ 8.54 (s, 1H), 8.20 (dd, *J* = 10.4, 8.7 Hz, 1H), 7.71 (dd, *J* = 11.3, 6.4 Hz, 1H), 4.37 (q, *J* = 7.1 Hz, 2H), 3.44 (tt, *J* = 7.2, 3.9 Hz, 1H), 1.43 – 1.34 (m, 3H), 1.37 – 1.31 (m, 2H), 1.28 – 1.13 (m, 2H); **^{13}C NMR** (101 MHz, $CDCl_3$) δ 172.5, 165.1, 151.9, 148.7, 147.3, 137.3, 115.2, 115.2, 115.0, 115.0, 110.7, 105.4, 105.2, 60.9, 34.6, 31.4, 29.5, 14.2, 8.0; **LRMS** *m/z* (ESI^-) 294.09 $[M+H]^+$. These data are in agreement with the literature.[7]

1-Cyclopropyl-6-fluoro-4-oxo-7-(piperazin-1-yl)-1,4-dihydroquinoline-3-carboxylic acid (1)

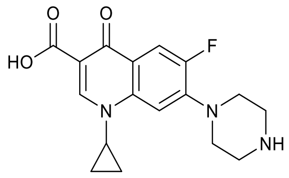

Step 1: Piperazine (370 mg, 4.30 mmol, 4.0 eq) was added to a suspension of **1b** (315 mg, 1.1 mmol, 1.0 eq) in dry MeCN (15 mL). The reaction was refluxed at 80 °C for 18 h. This intermediate (**1c**) was used in the next step without further purification.

Step 2: To **1c** was added 1 M NaOH (2.1 mL, 2.0 eq), and stirred for 2 h at 80 °C. The reaction mixture was then cooled, neutralised with 1 M HCl (3 mL), and allowed to precipitate at −20 °C. The white precipitate was filtered and washed with H_2O (20 mL). Excess H_2O was removed by azeotropic distillation with toluene (30 mL) to give **1** as a white solid (342 mg, 96%). R_f = 0.08 (SiO_2; DCM:MeOH, 80:20); **^{1}H NMR** (400 MHz, Acetic acid-*d*$_4$) δ 8.86 (s, 1H), 7.98 (d, J = 13.0 Hz, 1H), 7.66 (d, *J* = 7.2 Hz, 1H), 3.79 (tt, *J* = 7.3, 4.0 Hz, 1H), 3.71 (dd, *J* = 7.0, 3.4 Hz, 4H), 3.60 (dd, *J* = 6.7, 3.6 Hz, 4H), 1.45 (t, *J* = 6.6 Hz, 2H), 1.29 (dd, *J* = 6.6, 4.0 Hz, 2H); **HRMS** *m/z* (ESI$^+$) found 332.1419, $C_{17}H_{19}FN_3O_3$ ($[M+H]^+$) requires 332.1405. These data are in good agreement with the literature.[7]

8-Ethyl-5-oxo-2-(4-((3-(trifluoromethyl)phenyl)carbamothioyl)piperazin-1-yl)-5,8-dihydropyrido[2,3-*d*]pyrimidine-6-carboxylic acid (2)

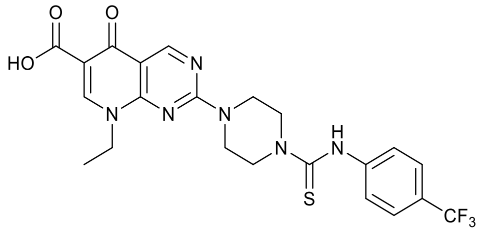

8-Ethyl-5-oxo-2-(piperazin-1-yl)-5,8-dihydropyrido[2,3-d]pyrimidine-6-carboxylic acid (200 mg, 0.66 mmol) and 3-(trifluoromethyl)phenyl isocyanate (270 mg, 1.3 mmol) were suspended in MeCN (3 mL) and stirred for 18 h at RT. MeCN was removed *in vacuo* and the resultant solid slurried in Et_2O (20 mL) and collected by filtration to give **2** as a white solid (270 mg, 80%). R_f = 0.68 (SiO_2; DCM:MeOH, 90:10); **^{1}H NMR** (400 MHz, DMSO) δ 14.79 (s, 1H), 9.64 (s, 1H), 9.26 (s, 1H), 8.99 (s, 1H), 7.75 - 7.45 (m, 4H), 4.44 (q, *J* = 7.0 Hz, 2H), 4.15 - 4.04 (m, 8H), 1.39 (t, *J* = 7.0 Hz, 3H); **LRMS** m/z (ESI$^+$) 507 ($[M+H]^+$); **HRMS** m/z (ESI$^+$) found 507.1426, $C_{22}H_{22}F_3N_6O_3S$ ($[M+H]^+$), requires 507.1421; **HPLC** Retention time 11.5 min, 99.8% (280 nm). These data are in agreement with the literature.[8]

1-Cyclopropyl-6-fluoro-4-oxo-7-(4-((4-(trifluoromethyl)phenyl)carbamothioyl) piperazin-1-yl)-1,4-dihydroquinoline-3-carboxylic acid (3)

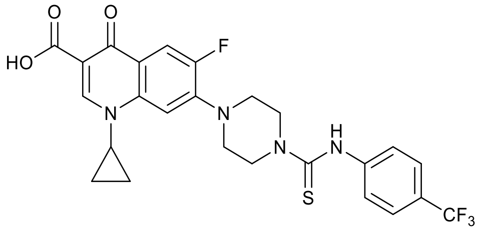

1 (200 mg, 0.60 mmol) and 4-(trifluoromethyl)phenyl isothiocyanate (243 mg, 1.2 mmol) were stirred in anhydrous MeCN (5 mL) for 18 h at RT. NH_4Cl (10 mL) was added and the suspension was stirred for 10 min. The product was filtered and washed with H_2O (10 mL) and Et_2O (10 mL) to give **3** as a white solid (302 mg, 92%). R_f = 0.78 (SiO_2; DCM:MeOH, 90:10); **^{1}H NMR** (400 MHz, DMSO-*d$_6$*) δ: 15.20 (s, 1H), 9.82 (s, 1H), 8.67 (s, 1H), 7.93 (d, *J* = 13.0 Hz, 1H), 7.67 (d, *J* = 8.7 Hz, 2H), 7.61 (d, *J* = 8.7 Hz, 2H), 7.57 (s, 1H), 4.19 (t, *J* = 4.0 Hz, 4H), 3.83 (m, 1H), 3.51 (t, *J* = 4.0 Hz, 4H), 1.36–1.31 (m, 2H), 1.22–1.18 (m, 2H); **HRMS** *m/z* (ESI$^+$) found 535.1437, $C_{25}H_{23}F_4N_4O_3S$ ([M+H])$^+$ requires 535.1422; **HPLC** Retention time 11.9 min, 99.2% (280 nm). These data are in good agreement with the literature.[9]

7-(4-(Cyclohexylcarbamothioyl)piperazin-1-yl)-1-cyclopropyl-6-fluoro-4-oxo-1,4-dihydroquinoline-3-carboxylic acid (4)

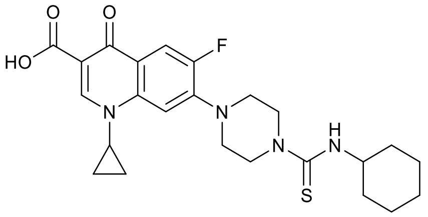

Prepared following *general procedure B* from **1** (150 mg, 0.45 mmol) and cyclohexyl isothiocyanate. Purification by flash silica column chromatography DCM:MeOH (0–10%) gave **4** as a white solid (84 mg, 40%). R_f = 0.75 (SiO_2; DCM:MeOH, 9:1); **^{1}H NMR** (400 MHz, $CDCl_3$: CD_3OD [2:1]) δ: 8.81 (s, 1H), 8.02 (d, *J* = 13.1 Hz, 1H), 7.44 (d, *J* = 7.1 Hz, 1H), 4.10 (t, *J* = 5.0 Hz, 4H), 3.70–3.62 (m, 1H), 3.48 (t, *J* = 5.0 Hz, 4H), 3.42–3.38 (m, 1H), 2.08 (d, *J* = 12.0 Hz, 2H), 1.84–1.63 (m, 3H), 1.49–1.34 (m, 4H), 1.32–1.15 (m, 5H); **LRMS** *m/z* (ESI$^+$) 473 ([M+H]$^+$). **HRMS** *m/z* (ESI$^+$) found 495.1849, $C_{24}H_{29}FN_4O_3SNa$ ([M+Na])$^+$ requires 495.1837.

1-Cyclopropyl-6-fluoro-7-(4-((2-morpholinoethyl)carbamothioyl)piperazin-1-yl)-4-oxo-1,4-dihydroquinoline-3-carboxylic acid (5)

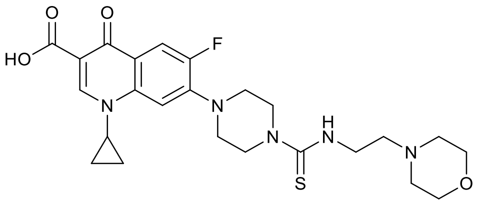

Prepared following *general procedure C* from **1** (150 mg, 0.45 mmol) and 2-(4-morpholino)ethyl isothiocyanate. Purification by flash silica column chromatography (0–10% MeOH/DCM [+1% Et_3N]) gave **5** as an off-white solid (35 mg, 15%). R_f = 0.50 (SiO_2; DCM:MeOH:Et_3N, 9:1:0.1); **^{1}H NMR** (400 MHz, $CDCl_3$: CD_3OD [2:1]) δ: 8.63 (s, 1H), 7.96 (d, *J* = 13.4 Hz, 1H), 7.36 (d, *J* = 7.1 Hz, 1H), 4.06 (t, *J* = 5.2 Hz, 4H), 3.79 (t, *J* = 6.6 Hz, 2H), 3.72 (t, *J* = 4.7 Hz, 4H), 3.52–3.42 (m, 1H), 3.39–3.33 (m, 4H), 2.64 (t, *J* = 6.5 Hz, 2H), 2.54 (t, *J* = 4.7 Hz, 4H), 1.35–1.26 (m, 2H), 1.18–1.11 (m, 2H); **LRMS** *m/z* (ESI^+) 504 ($[M+H]^+$); **HRMS** *m/z* (ESI^+) found 504.2088, $C_{24}H_{31}FN_5O_4S$ ($[M+H]^+$) requires 504.2075.

7-(4-((4-Cyanophenyl)carbamothioyl)piperazin-1-yl)-1-cyclopropyl-6-fluoro-4-oxo-1,4-dihydroquinoline-3-carboxylic acid (6)

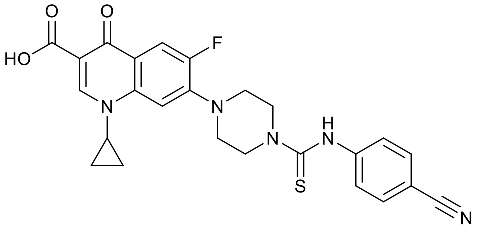

Prepared following *general procedure C* from **1** (230 mg, 0.69 mmol) and 4-cyano phenyl isothiocyanate, using $NaHCO_3$ instead of Cs_2CO_3. Purification by flash silica column chromatography (0–20% MeOH/DCM [+1% Et_3N]) gave **6** as a pale-yellow solid (32 mg, 10%). R_f = 0.53 (SiO_2; DCM:MeOH, 9:1); **^{1}H NMR** (400 MHz, $CDCl_3$:CD_3OD [3:1]) δ: 8.78 (s, 1H), 8.00 (d, *J* = 13.0 Hz, 1H), 7.64–7.54 (m, 2H), 7.52–7.46 (m, 2H), 7.43 (d, *J* = 7.2 Hz, 1H), 4.19 (t, *J* = 5.0 Hz, 4H), 3.65–3.58 (m, 1H), 3.48 (d, *J* = 5.3 Hz, 4H), 1.41 (d, *J* = 6.8 Hz, 2H), 1.24–1.15 (m, 2H); **LRMS** *m/z* (ESI^-) 490 ($[M-H]^-$); **HRMS** *m/z* (ESI^-) found 490.1360, $C_{25}H_{21}FN_5O_3S$ ($[M-H]^-$) requires 490.1355.

1-Cyclopropyl-6-fluoro-4-oxo-7-(4-(pyridin-4-ylcarbamothioyl)piperazin-1-yl)-1,4-dihydroquinoline-3-carboxylic acid (7)

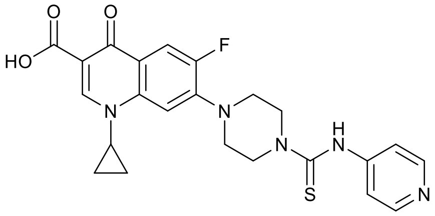

Step 1: 4-Isothiocyanatopyridine was prepared following *general procedure A* from 4-aminopyridine (96 mg, 1.0 mmol). This intermediate (**7a**) was used in the next step without further purification.

Step 2: Prepared following *general procedure C* from intermediate **7a** and **1**. Purification by flash silica column chromatography (0–20% MeOH/DCM [+1% Et_3N]) gave **7** as an orange solid (8 mg, 4%). R_f = 0.20 (SiO_2; DCM:MeOH:Et_3N, 90:9:1); **^{1}H NMR** (400 MHz, $CDCl_3$:CD_3OD [3:1]) δ: 8.71 (s, 1H), 8.33 (d, *J* = 5.7 Hz, 2H), 7.99 (d, *J* = 13.2 Hz, 1H), 7.49–7.41 (m, 3H), 4.20 (t, *J* = 5.1 Hz, 4H), 3.65–3.51 (m, 1H), 3.49–3.42 (m, 4H), 1.37 (d, *J* = 6.9 Hz, 2H), 1.25–1.12 (m, 2H); **LRMS** *m/z* (ESI^-) 466 ($[M{-}H]^-$); **HRMS** *m/z* (ESI^-) found 466.1338, $C_{23}H_{21}N_5O_3S$ ($[M{-}H]^-$) requires 466.1355.

7-(4-(1*H* -Imidazole-1-carbonothioyl)piperazin-1-yl)-1-cyclopropyl-6- fluoro-4-oxo-1,4-dihydroquinoline-3-carboxylic acid (8)

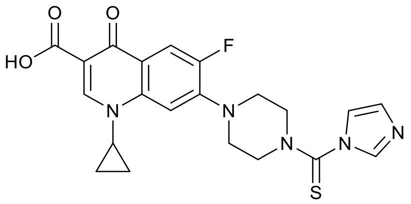

1 (150 mg, 0.45 mmol, 1.0 eq) was added to a solution of 1,1′-thiocarbonyldiimidazole (90 mg, 0.50 mmol, 1.1 eq), and $NaHCO_3$ (45 mg, 0.50 mmol, 1.1 eq) in anhydrous DCM:MeCN 1:1 (10 mL). The reaction was stirred at RT for 18 h. NH_4Cl (12.5 mL) was added, the product extracted with DCM (3 × 30 mL), washed with H_2O (3 × 30 mL), brine (30 mL), dried, and the solvent removed *in vacuo*. Purification by flash silica column chromatography (0–10% MeOH/DCM [+1% Et_3N]) gave **8** as an off-white solid (163 mg, 82%). R_f = 0.75 (SiO_2; DCM:MeOH, 9:1); **^{1}H NMR** (400 MHz, $CDCl_3$:CD_3OD [4:1]) δ: 8.75 (s, 1H), 8.03–7.93 (m, 2H), 7.45 (d, *J* = 7.1 Hz, 1H), 7.32 (t, *J* = 1.4 Hz, 1H), 7.07 (t, *J* = 1.3 Hz, 1H), 4.20–4.09 (m, 4H), 3.69–3.57 (m, 1H), 3.52 (t, *J* = 5.1 Hz, 4H), 1.46–1.37 (m, 2H), 1.25–1.11 (m, 3, 2H); **LRMS** *m/z* (ESI-) 440 ($[M{-}H]^-$); **HRMS** *m/z* (ESI^-) found 440.1207, $C_{21}H_{19}FN_5O_3S$ ($[M{-}H]^-$) requires 440.1198.

1-Cyclopropyl-6-fluoro-4-oxo-7-(4-(phenoxycarbonothioyl)piperazin-1-yl)-1,4-dihydroquinoline-3-carboxylic acid (9)

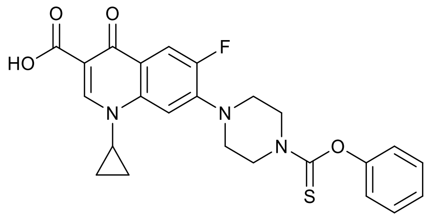

1 (170 mg, 0.50 mmol, 1.0 eq) was added to a solution of *O*-phenyl chlorothioformate (69 µL, 0.50 mmol, 1.0 eq), and $NaHCO_3$ (46 mg, 0.50 mmol, 1 eq) in anhydrous MeCN (10 mL). The reaction was stirred at RT for 1h. NH_4Cl (20 mL) was added, the product extracted with DCM (3 × 30 mL) and EtOAc (30 mL), dried, and the solvent removed *in vacuo*. The resulting residue was purified by flash silica column chromatography (0–20% MeOH/DCM [+1% Et_3N]) to give **9** as a yellow solid (178 mg, 84%). R_f = 0.48 (SiO_2; DCM:MeOH, 9:1); **^{1}H NMR** (400 MHz, $CDCl_3$:MeOD [4:1]) δ: 8.78 (s, 1H), 8.01 (d, *J* = 12.9 Hz, 1H), 7.47 (d, *J* = 7.1 Hz, 1H), 7.44–7.33 (m, 2H), 7.29–7.21 (m, 1H), 7.09–7.01 (m, 2H), 4.39 (t, *J* = 5.2 Hz, 2H), 4.22 (t, *J* = 5.1 Hz, 2H), 3.62 (tt, *J* = 7.2, 3.9 Hz, 1H), 3.55–3.46 (m, 4H), 1.42 (d, *J* = 6.8 Hz, 2H), 1.29–1.16 (m, 2H); **LRMS** *m/z* (ESI^-) 466 ($[M-H]^-$); **HRMS** *m/z* (ESI^-) found 466.1275, $C_{24}H_{21}FN_3O_4S$ ($[M-H]^-$) requires 466.1242.

1-Cyclopropyl-6-fluoro-4-oxo-7-(4-(2-oxo-1,2-dihydropyridine-1-carbonothioyl) piperazin-1-yl)-1,4-dihydroquinoline-3-carboxylic acid (10)

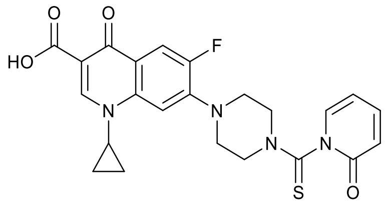

1 (150 mg, 0.45 mmol, 1 eq) was added to a solution of 1,1-thiocarbonyldi-2(1*H*)-pyridone (110 mg, 0.45 mmol, 1 eq), and $NaHCO_3$ (46 mg, 0.50 mmol, 1.1 eq) in anhydrous MeCN (10 mL). The mixture was stirred overnight at RT. NH_4Cl (20 mL) was added, and the product was extracted with DCM (3 × 30 mL) and EtOAc (30 mL), dried, and the solvent was removed *in vacuo*. Purification by flash silica column chromatography (0–10% MeOH/DCM) gave **10** as a clear crystalline solid (156 mg, 74%). R_f = 0.65 (SiO_2; DCM:MeOH, 9:1); **^{1}H NMR** (400 MHz, DMSO-d_6) δ: 15.18 (s, 1H), 8.67 (s, 1H), 7.94 (d, *J* = 13.2 Hz, 1H), 7.69 (ddd, *J* = 7.0, 2.1, 0.8 Hz, 1H), 7.61–7.49 (m, 2H), 6.44 (dt, *J* = 9.4, 1.0 Hz, 1H), 6.39 (td, *J* = 6.7, 1.2 Hz, 1H), 4.46 (ddd, *J* = 10.0, 6.0, 3.1 Hz, 1H), 4.38 (ddd, *J* = 13.5, 6.7, 4.1 Hz, 1H), 3.81

(tt, *J* = 7.3, 4.1 Hz, 1H), 3.73–3.59 (m, 4H), 3.49 (t, *J* = 5.2 Hz, 2H), 1.37–1.27 (m, 2H), 1.21–1.12 (m, 2H); **LRMS** *m/z* (ESI$^+$) 469 ([M+H]$^+$); **HRMS** *m/z* (ESI+) found 469.1161, $C_{23}H_{21}FN_4O_4SNa$ ([M+Na]$^+$) requires 491.1160.

1-Cyclopropyl-6-fluoro-4-oxo-7-(4-((4-(trifluoromethyl)phenyl)carbamoyl)piperazin-1-yl)-1,4-dihydroquinoline-3-carboxylic acid (11)

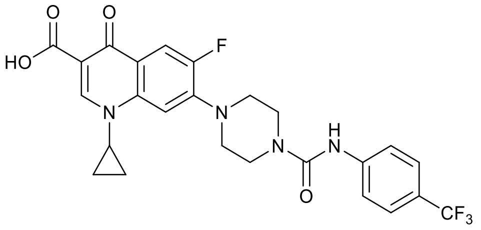

4-(trifluoromethyl)phenyl isocyanate (88 mg, 0.47 mmol) was added to a suspension of **1** (200 mg, 0.36 mmol) and Cs_2CO_3 (120 mg, 0.36 mmol) in 1:1 anhydrous MeCN/DCM. The reaction was stirred at 60 °C overnight. NH_4Cl (20 mL) was added, the product extracted with DCM (3 × 30 mL), washed with water (30 mL), dried, and the solvent removed *in vacuo*. Purification by flash column chromatography (0–10% MeOH/DCM) gave **11** as a colourless solid (150 mg, 80%). R_f = 0.51 (SiO_2; DCM:MeOH, 9:1); **^{1}H NMR** (400 MHz, DMSO-*d$_6$*) δ: 15.21 (br s, 1H), 9.09 (s, 1H), 8.68 (s, 1H), 7.95 (d, *J* = 13.1 Hz, 1H), 7.71 (d, *J* = 8.5 Hz, 2H), 7.63–7.58 (m, 4H), 3.87–3.83 (m, 1H), 3.75–3.69 (m, 4H), 1.35–1.28 (m, 2H), 1.22–1.16 (m, 2H)[1]; **LRMS** *m/z* (ESI$^+$) 519 ([M+H]$^+$); **HRMS** *m/z* (ESI$^+$) found 519.1627, $C_{25}H_{23}F_4N_4O_4$ ([M+H]$^+$) requires 519.1627; **HPLC** Retention time 11.5 min, 99.4% (280 nm).

1-Cyclopropyl-6-fluoro-4-oxo-7-(4-(4-(trifluoromethyl)benzoyl)piperazin-1-yl)-1,4-dihydroquinoline-3-carboxylic acid (12)

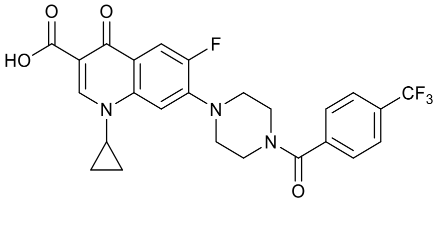

4-(Trifluoromethyl)benzoic acid (93 mg, 0.49 mmol, 1.0 eq) was dissolved in anhydrous DMF (5 mL) at 0 °C. Afterwards, Et_3N (205 µl, 1.5 mmol, 3.1 eq) and HATU (166 mg, 0.49 mmol, 1.0 eq) were added

[1] Second piperazine 4H obscured by water peak.

and the reaction mixture stirred at 0 °C for 30 min. Then, **1** (221 mg, 0.64 mmol, 1.3 eq) was added and the reaction stirred at RT for 16 h. The reaction mixture was filtered, and saturated NH_4Cl was added to the supernatant until a precipitate was formed. The precipitate was collected by filtration. Purification by flash silica column chromatography (0–2% MeOH/DCM) gave **12** as a white solid (100 mg, 41%). R_f = 0.90 (SiO_2; DCM:MeOH, 9:1); **^{1}H NMR** (400 MHz, $CDCl_3$) δ: 14.84 (s, 1H), 8.64 (s, 1H), 7.88 (d, *J* = 12.7 Hz, 1H), 7.71 (d, *J* = 7.8 Hz, 2H), 7.57 (d, *J* = 7.8 Hz, 2H), 7.34 (d, *J* = 7.0 Hz, 1H), 4.18–3.20 (m, 9H), 1.48–1.28 (m, 2H), 1.27–1.08 (m, 2H); **LRMS** *m/z* (ESI^+) 504 ($[M+H]^+$); **HRMS** *m/z* (ESI^+) found 504.1562, $C_{25}H_{21}F_4N_3O_4$ ($[M+H]^+$) requires 504.1541.

1-Cyclopropyl-6-fluoro-4-oxo-7-(4-((4-(trifluoromethyl)phenyl)sulfonyl)piperazin-1-yl)-1,4-dihydroquinoline-3-carboxylic acid (13)

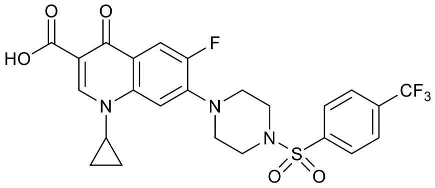

1 (150 mg, 0.45 mmol, 1.0 eq) was added to a suspension of 4-(trifluoromethyl)benzenesulfonyl chloride (110 mg, 0.45 mmol, 1.0 eq) and Cs_2CO_3 (147 mg, 0.45 mmol, 1.0 eq) in anhydrous DCM (10 mL). The reaction was heated under reflux for 18 h. NH_4Cl (30 mL) was added, and the product extracted with DCM (3 × 30 mL), washed with H_2O (30 mL), dried, and the solvent removed *in vacuo*. The residue was purified by flash silica column chromatography (0–10% MeOH/DCM) to give **13** as a pale-yellow solid (194 mg, 80%). R_f = 0.55 (SiO_2; DCM:MeOH, 9:1); **^{1}H NMR** (400 MHz, $CDCl_3$:CD_3OD [4:1]) δ: 8.73 (s, 1H), 7.97–7.90 (m, 3H), 7.85 (d, *J* = 8.3 Hz, 2H), 7.40 (d, *J* = 7.1 Hz, 1H), 3.59 (tt, *J* = 7.2, 4.0 Hz, 1H), 3.40 (dd, *J* = 6.3, 3.7 Hz, 4H), 3.27 (dd, *J* = 6.2, 3.4 Hz, 4H), 1.44–1.35 (m, 2H), 1.25–1.13 (m, 2H); **^{13}C NMR** (101 MHz, $CDCl_3$:CD_3OD [4:1]) 147.81, 128.20, 126.54, 112.24, 105.59, 49.09, 45.76, 35.46, 8.20; **LRMS** *m/z* (ESI^+) 540 ($[M+H]^+$); **HRMS** *m/z* (ESI^+) found 540.1204, $C_{24}H_{22}F_4N_3O_5S$ ($[M+H]^+$) requires 540.1211.

1-Cyclopropyl-6-fluoro-4-oxo-7-(4-(6-(trifluoromethyl)benzo[*d*]thiazol-2 -yl)piperazin-1-yl)-1,4-dihydroquinoline-3-carboxylic acid (14)

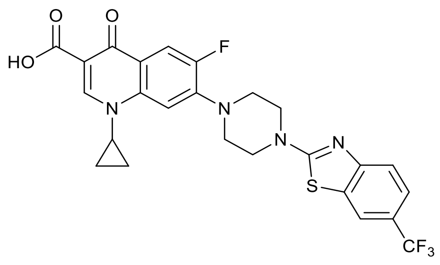

Step 1: 2-Iodo-1-isothiocyanato-4-(trifluoromethyl)benzene was prepared following *general procedure A* from 4-amino-3-iodobenzotrifluoride (290 mg, 1.0 mmol). This intermediate (**14a**) was used in the next step without further purification.

Step 2: Prepared following *general procedure C* from intermediate **14a** and **1**. Purification by flash silica column chromatography (0–10% MeOH/DCM) gave **14** as a pale-yellow solid (172 mg, 43%). **^{1}H NMR** (400 MHz, DMSO-d_6) δ: 15.18 (s, 1H), 8.68 (s, 1H), 8.29 (s, 1H), 7.96 (d, *J* = 13.0 Hz, 1H), 7.64 (d, *J* = 7.4 Hz, 1H), 7.62–7.60 (m, 2H), 3.90–3.86 (m, 4H), 3.83 (dt, *J* = 7.0, 3.2 Hz, 1H), 3.56–3.48 (m, 4H), 1.32 (dd, *J* = 7.5, 5.4 Hz, 2H), 1.27–1.15 (m, 2H); **LRMS** *m/z* (ESI$^+$) 533 ([M+H]$^+$); **HRMS** *m/z* (ESI$^+$) found 533.1277, $C_{25}H_{21}FN_4O_3S$ ([M+H]$^+$) requires 533.1265.

7-(4-((Benzylthio)((4-(trifluoromethyl)phenyl)imino)methyl)piperazin-1-yl)-1-cyclopropyl-6-fluoro-4-oxo-1,4-dihydroquinoline-3-carboxylic acid (15)

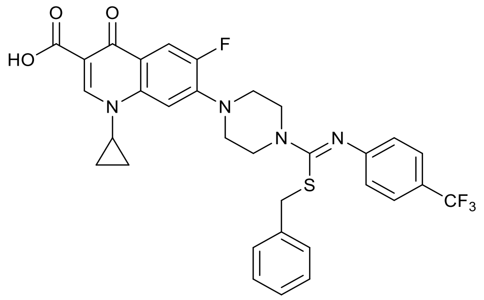

3 (70 mg, 0.13 mmol) and K_2CO_3 (18 mg, 0.13 mmol) were stirred at RT in anhydrous DMF (2 mL) and benzyl bromide (11 mg, 0.18 mmol) was added. After 16 h, NH_4Cl (10 mL) was added, and the suspension was stirred for 10 min. The product was extracted with DCM (3 × 10 mL), dried over Mg_2SO_4, and purified by flash column chromatography DCM:MeOH (0–5%) to give **15** as a white solid (39 mg, 48%). R_f = 0.83 (SiO_2; DCM:MeOH, 9:1); **^{1}H NMR** (400 MHz, $CDCl_3$) δ 14.95 (s, 1H), 8.77 (s, 1H), 8.04 (d, *J* = 12.9 Hz, 1H), 7.52 (d, *J* = 8.3 Hz, 2H), 7.36–7.11 (m, 6H), 6.91 (d, *J* = 8.3 Hz, 2H), 3.85 (t, *J* = 4.0 Hz, 4H), 3.63 (s, 2H), 3.55 (sep, *J* = 4.0 Hz, 1H), 3.27 (t, *J* = 4.0 Hz, 4H), 1.44–1.39 (m, 2H), 1.26–1.20 (m, 2H); **LRMS** *m/z* (ESI$^+$) 625.2 ([M+H]$^+$); **HRMS** *m/z* (ESI$^+$) found 625.1907, $C_{32}H_{28}F_4N_4O_3S$ ([M+H]$^+$), requires 625.1891.

1-Cyclopropyl-6-fluoro-7-(4-(((2-methoxy-2-oxoethyl)thio)((4-(trifluoromethyl)phenyl)imino)methyl)piperazin-1-yl)-4-oxo-1,4-dihydroquinoline-3-carboxylic acid (16)

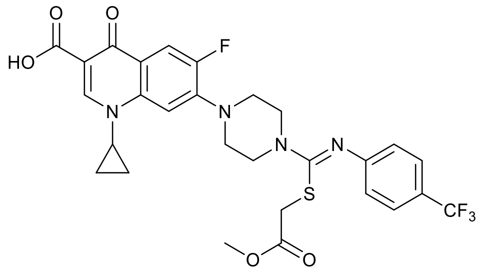

Na_2CO_3 (25 mg, 0.19 mmol), and methyl 2-bromoacetate (29 mg, 0.19 mmol) were added to a solution of **3** (100 mg 0.19 mmol) in dry DMF (2 mL). The reaction was stirred at RT for 16 h. The reaction mixture was poured into NH_4Cl (15 mL), and the precipitated was filtered, washed with H_2O (20 mL), and dried over Mg_2SO_4. Purification by flash silica column chromatography (1–2% MeOH/DCM) gave **16** as an off-white solid (40 mg, 35%). R_f = 0.70 (SiO_2; DCM:MeOH, 90.10); **^{1}H NMR** (400 MHz, $CDCl_3$) δ 14.91 (s, 1H), 8.69 (s, 1H), 7.95 (d, *J* = 13.0 Hz, 1H), 7.54–7.49 (m, 2H), 7.37 (d, *J* = 7.0 Hz, 1H), 7.01–6.96 (m, 2H), 3.91 (t, *J* = 5.0 Hz, 4H), 3.70 (s, 3H), 3.60–3.52 (m, 1H), 3.42 (t, *J* = 5.0 Hz, 4H), 3.25 (s, 2H), 1.43–1.36 (m, 2H), 1.24–1.17 (m, 2H); **HRMS** *m/z* (ESI$^+$) found 607.1633, $C_{28}H_{27}F_4N_4O_5S$ ($[M+H]^+$) requires 607.1633.

7-(4-(((2-Amino-2-oxoethyl)thio)((4-(trifluoromethyl)phenyl)imino)methyl)piperazin-1-yl)-1-cyclopropyl-6-fluoro-4-oxo-1,4-dihydroquinoline-3-carboxylic acid (17)

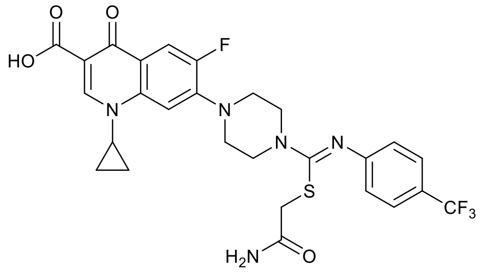

To a suspension of **3** (100 mg, 0.19 mmol, 1.0 eq) in anhydrous DMF (10 mL) was added 2-bromoacetamide (26 mg, 0.19 mmol, 1.0 eq), and K_2CO_3 (26 mg, 0.19 mmol, 1.0 eq). The reaction mixture was stirred for 4 h at RT and then NH_4Cl (15 mL) was added. The product was extracted with DCM (3 × 30mL), washed with H_2O (20 mL), dried over Mg_2SO_4, and the solvent removed *in vacuo*. The residue was purified by flash silica column chromatography (0–10% MeOH/DCM), to give **17** as a white

solid (35 mg, 32%). R_f = 0.5 (SiO_2; DCM:MeOH, 9:1); **^{1}H NMR** (400 MHz, DMSO-d_6) δ: 15.21 (s, 1H), 8.68 (s, 1H), 7.95 (d, *J* = 13.2 Hz, 1H), 7.78–7.52 (m, 3H), 7.46 (s, 1H), 7.15–7.10 (m, 1H), 7.04–6.96 (m, 2H), 3.87–3.82 (m, 1H), 3.80 (t, *J* = 5.0 Hz, 4H), 3.45 (t, *J* = 4.7 Hz, 4H), 3.30 (s, 2H), 1.40–1.28 (m, 2H), 1.28–1.15 (m, 2H); **HRMS** *m/z* (ESI^-) found 590.1473, $C_{27}H_{24}F_4N_5O_4S$ ($[M-H]^-$) requires 590.1480.

1-cyclopropyl-6,7-difluoro-4-oxo-1,4-dihydroquinoline-3-carboxylic acid (18a)

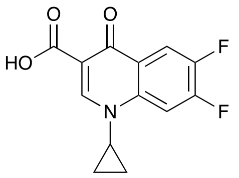

A suspension of **1b** (16 mg, 0.054 mmol) in 1M NaOH (1 mL) and THF (2 mL) was heated to 50 °C for 18 h. The reaction mixture was cooled and neutralised with 1 M HCl. The THF was removed *in vacuo*, and the precipitate was filtered, washed with Et_2O (5 mL) and **18a** collected as a yellow solid (12 mg, 83%) R_f = 0.82 (SiO_2; DCM:MeOH, 9:1); **^{1}H NMR** (400 MHz, DMSO-d_6) δ: 8.71 (s, 1H), 8.31–8.20 (m, 2H), 1.33–1.26 (m, 2H), 1.17–1.13 (m, 2H).[2] **LRMS** *m/z* (ESI^+) 266 ($[M+H]^+$); **HRMS** *m/z* (ESI^+) found 266.0636, $C_{13}H_{10}F_2NO_3$ ($[M+H]^+$) requires 266.0623.

7-(4-((*tert*-Butoxycarbonyl)amino)piperidin-1-yl)-1-cyclopropyl-6-fluoro-4-oxo-1,4-dihydroquinoline-3-carboxylic acid (18b)

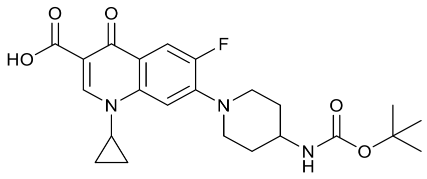

Prepared following *general procedure E* from 4-*N*-Boc-amino-piperidine (800 mg, 4.0 mmol, 4.0 eq) and **18a** (265 mg, 1.0 mmol, 1.0 eq). Purification by flash silica column chromatography (0–10% MeOH/EtOAc) gave **18b** as an off-white solid (435 mg, 98%). **^{1}H NMR** (400 MHz, $CDCl_3$) δ: 15.01 (s, 1H), 8.76 (s, 1H), 8.00 (d, *J* = 13.0 Hz, 1H), 7.36 (d, *J* = 7.1 Hz, 1H), 4.54 (m, 1H), 3.71 (dd, *J* = 12.0, 4.8 Hz, 3H), 3.54 (tt, *J* = 7.1, 4.0 Hz, 1H), 3.00 (m, 2H), 2.15 (m, 2H), 1.66 (m, 2H), 1.47 (s, 9H), 1.39 (m, 2H), 1.23 (m, 2H).

[2] Tertiary C-H on cyclopropyl obscured by water peak.

1-(3-Carboxy-1-cyclopropyl-6-fluoro-4-oxo-1,4-dihydroquinolin-7-yl)piperidin-4-aminium chloride (18c)

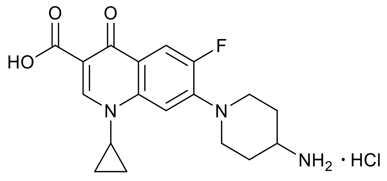

Prepared following *general procedure D* from **18b** (400 mg, 0.90 mmol), to give **18c** as a yellow solid (275 mg, 89%). **^{1}H NMR** (400 MHz, DMSO-d_6) δ: 8.57 (s, 1H), 8.28 (m, 3H), 7.91 (d, *J* = 13.2 Hz, 1H), 7.58 (d, *J* = 7.6 Hz, 1H), 3.87 - 3.71 (m, 3H), 3.28 (m, 1H), 3.09 - 2.90 (m, 2H), 2.14 - 2.06 (m, 2H), 1.77 (m, 2H), 1.37 - 1.15 (m, 4H).

1-Cyclopropyl-6-fluoro-4-oxo-7-(4-(3-(4-(trifluoromethyl)phenyl)thioureido)piperidin-1-yl)-1,4-dihydroquinoline-3-carboxylic acid (18)

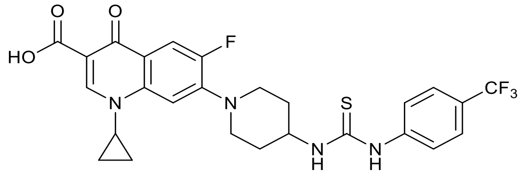

Prepared following *general procedure C* from **18c** (139 mg, 0.37 mmol). Purification by flash silica column chromatography (0–10% MeOH/EtOAc) gave **18** as pale-yellow solid (98 mg, 49%). R_f = 0.70 (SiO_2; DCM:MeOH, 9:1); **^{1}H NMR** (400 MHz, DMSO-d_6) δ: 15.22 (s, 1H), 9.79 (s, 1H), 8.65 (s, 1H), 8.14 (d, *J* = 7.3 Hz, 1H), 7.88 (d, *J* = 13.2 Hz, 1H), 7.75 (d, *J* = 8.6 Hz, 3H), 7.65 (d, *J* = 8.6 Hz, 3H), 7.59 (d, *J* = 7.6 Hz, 1H), 4.40 (s, 1H), 3.85–3.77 (m, 1H), 3.76–3.68 (m, 2H), 3.11 (t, *J* = 11.7 Hz, 2H), 2.22–2.11 (m, 2H), 1.73 (q, *J* = 11.6 Hz, 2H), 1.39–1.28 (m, 2H), 1.19 (t, *J* = 3.0 Hz, 2H); **HRMS** *m/z* (ESI$^+$) found 549.1586, $C_{26}H_{25}F_4N_4O_3S$ ([M+H]$^+$) requires 549.1578.

4-(2-Fluorophenyl)-*N*-(4-(trifluoromethyl)phenyl)piperazine-1-carbothioamide (19)

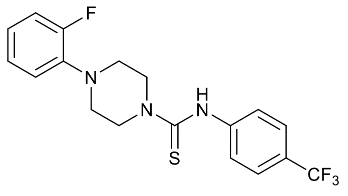

Prepared following *general procedure C* from 1-(2-fluorophenyl)piperazine (410 mg, 1.4 mmol) and 4-(trifluoromethyl)phenyl isothiocyanate. Purification by flash silica column chromatography (0–70% EtOAc/petroleum ether) gave **19** as a white solid (204 mg, 96%). R_f = 0.75 (SiO_2; Petroleum ether:EtOAc, 1:1); **^{1}H NMR** (400 MHz, $CDCl_3$) δ: 7.59 (d, *J* = 8.4 Hz, 2H), 7.33 (s, 1H), 7.25 (d, *J* = 8.5 Hz, 2H), 7.13–6.90 (m, 4H), 4.07–4.00 (m, 4H), 3.21–3.15 (m, 4H); **LRMS** *m/z* (ESI+) 384 ($[M+H]^+$), *m/z* (ESI^-) 382 ($[M-H]^-$); **HRMS** *m/z* (ESI^-) found 382.1008, $C_{18}H_{16}F_4N_3S$ ($[M-H]^-$) requires 382.1007.

Ethyl 4-(4-((4-(trifluoromethyl)phenyl)carbamothioyl)piperazin-1-yl)benzoate (20)

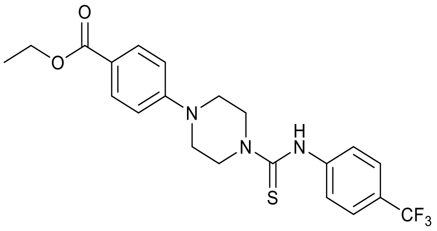

Prepared following *general procedure C* from 4-(piperazin-1-yl)-benzoic acid ethyl ester (230 mg, 1.0 mmol) and 4-(trifluoromethyl)phenyl isothiocyanate. Purification by flash silica column chromatography (0–70% EtOAc/petroleum ether) gave **20** as a colourless solid (296 mg, 68%). R_f = 0.60 (SiO_2; Petroleum ether:EtOAc, 1:1); **^{1}H NMR** (400 MHz, $CDCl_3$) δ: 7.95 (d, *J* = 9.0 Hz, 2H), 7.60 (d, *J* = 8.4 Hz, 2H), 7.33 (s, 1H), 7.31 (d, *J* = 8.4 Hz, 2H), 6.81 (d, *J* = 9.0 Hz, 2H), 4.33 (q, *J* = 7.1 Hz, 2H), 4.12–4.02 (m, 4H), 3.55–3.46 (m, 4H), 1.37 (t, *J* = 7.1 Hz, 3H); **^{13}C NMR** (101 MHz, $CDCl_3$) δ: 131.19, 126.54, 122.55, 113.24, 60.39, 48.42, 46.43, 14.52; **LRMS** *m/z* (ESI+) 438 ($[M+H]^+$), *m/z* (ESI−) 436 ($[M-H]^-$); **HRMS** *m/z* (ESI^-) found 436.1310, $C_{21}H_{21}F_3N_3O_2S$ ($[M-H]^-$) requires 436.1301.

1-(3-Fluoro-4-(piperazin-1-yl)phenyl)ethan-1-one (21a)

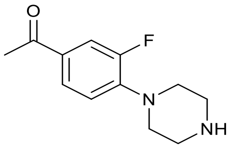

Prepared following *general procedure E* from 3,4-difluoroacetophenone (230 mg, 1.5 mmol) and piperazine, to give **21a** as a white solid (319 mg, 96%). **^{1}H NMR** (400 MHz, DMSO-d_6) δ: 7.71 (dd, *J* = 8.5, 2.1 Hz, 1H), 7.62 (dd, *J* = 14.5, 2.0 Hz, 1H), 7.06 (t, *J* = 8.7 Hz, 1H), 3.13–3.01 (m, 4H), 2.83 (dd, *J* = 4.5, 2.3 Hz, 4H), 2.50 (s, 3H). These data are in agreement with the literature.[10]

4-(4-Acetyl-2-fluorophenyl)-*N*-(4-(trifluoromethyl)phenyl)piperazine-1-carbothioamide (21)

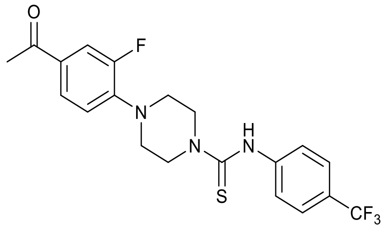

Prepared following *general procedure C* from **21a** (234 mg, 1.4 mmol) and 4-(trifluoromethyl)phenyl isothiocyanate. Purification by flash silica column chromatography (0–70% EtOAc/petroleum ether) gave **21** as a white solid (383 mg, 59%). R_f = 0.60 (SiO_2; petroleum ether:EtOAc, 1:1); **^{1}H NMR** (400 MHz, DMSO-d_6) δ: 9.68 (s, 1H), 7.73 (dd, *J* = 8.4, 2.1 Hz, 1H), 7.70–7.60 (m, 3H), 7.55 (d, *J* = 8.5 Hz, 2H), 7.12 (t, *J* = 8.7 Hz, 1H), 4.08 (t, *J* = 5.1 Hz, 4H), 3.35–3.27 (m, 4H), 2.50 (s, 3H); **^{13}C NMR** (101 MHz, DMSO-d_6) δ: 126.20, 125.54, 124.54, 118.56, 116.23, 115.90, 49.42, 48.42, 40.11, 26.82; **LRMS** *m/z* (ESI$^-$) 424 ([M−H]$^-$); **HRMS** *m/z* (ESI-) found 424.1130, $C_{20}H_{18}F_4N_3OS$ ([M−H]$^-$) requires 424.1112.

3-Fluoro-4-(piperazin-1-yl)benzonitrile (22a)

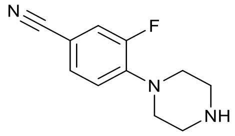

Prepared following *general procedure E* from 3,4-difluorobenzonitrile (230 mg, 1.5 mmol) and piperazine, to give **22a** as an off-white solid (278 mg, 91%). **^{1}H NMR** (400 MHz, DMSO-d_6) δ: 7.66 (dd, *J* = 13.6, 1.9 Hz, 1H), 7.54 (dd, *J* = 8.5, 2.0 Hz, 1H), 7.09 (t, *J* = 8.8 Hz, 1H), 3.10–3.03 (m, 4H), 2.85–2.78 (m, 4H). These data are in agreement with the literature.[11]

4-(4-Cyano-2-fluorophenyl)-*N* -(4-(trifluoromethyl)phenyl)piperazine-1-carbothioamide (22)

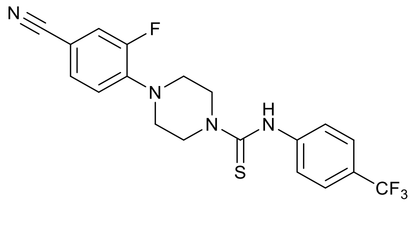

Prepared following *general procedure C* from **22a** (278 mg, 1.4 mmol) and 4-(trifluoromethyl)phenyl isothiocyanate. Purification by flash silica column chromatography (0–100% EtOAc/petroleum ether) gave **22** as a white solid (323 mg, 56%). R_f = 0.55 (SiO_2; petroleum ether:EtOAc, 1:1); **^{1}H NMR** (400 MHz,

DMSO-d_6) δ: 9.69 (s, 1H), 7.70 (dd, *J* = 13.3, 2.0 Hz, 1H), 7.64 (d, *J* = 8.6 Hz, 2H), 7.61–7.52 (m, 3H), 7.15 (t, *J* = 8.8 Hz, 1H), 4.11–4.04 (m, 4H), 3.33 (t, *J* = 5.1 Hz, 4H); **LRMS** *m/z* (ESI^-) 407 ($[M-H]^-$); **HRMS** *m/z* (ESI-) found 407.0984, $C_{19}H_{15}F_4N_4S$ ($[M-H]^-$) requires 407.0959.

3-Fluoro-4-(piperazin-1-yl)benzamide (23a)

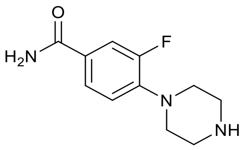

Prepared following *general procedure E* from 3,4-difluorobenzamide (240 mg, 1.5 mmol) and piperazine, to give **23a** as a white solid (240 mg, 71%). **^{1}H NMR** (400 MHz, DMSO-d_6) δ: 7.95 (s, 1H), 7.69–7.57 (m, 2H), 7.55 (s, 1H), 7.03 (t, *J* = 8.7 Hz, 1H), 3.03 (dd, *J* = 6.1, 3.5 Hz, 4H), 2.89 (d, *J* = 5.9 Hz, 4H).

3-Fluoro-4-(4-((4-(trifluoromethyl)phenyl)carbamothioyl)piperazin-1-yl) benzamide (23)

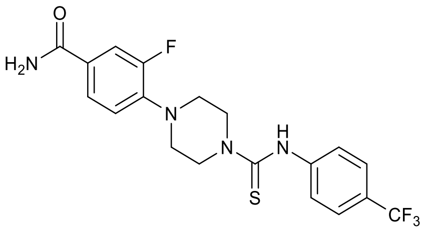

Prepared following *general procedure C* from **23a** (278 mg, 1.4 mmol) and 4-(trifluoromethyl)phenyl isothiocyanate. Purification by flash silica column chromatography (0–10% MeOH/DCM) gave **23** as a white solid (38 mg, 8%). R_f = 0.65 (SiO_2; DCM:MeOH, 9:1); **^{1}H NMR** (400 MHz, DMSO-d_6) δ: 9.69 (s, 1H), 7.90 (s, 1H), 7.73–7.63 (m, 4H), 7.55 (d, *J* = 8.5 Hz, 2H), 7.32 (s, 1H), 7.17–7.06 (m, 1H), 4.08 (t, *J* = 4.9 Hz, 4H), 3.22 (t, *J* = 5.0 Hz, 4H); **LRMS** *m/z* (ESI-) 425 ($[M-H]^-$); **HRMS** *m/z* (ESI-) found 425.1068, $C_{19}H_{17}F_4N_4OS$ ($[M-H]^-$) requires 425.1065.

Methyl 2-amino-5-fluoro-4-(piperazin-1-yl)benzoate (24a)

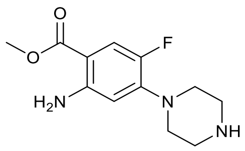

Prepared following *general procedure E* from methyl 2-amino-4,5-difluorobenzoate (200 mg, 1.1 mmol) and piperazine. Purification by flash silica column chromatography (5–15% MeOH/DCM [+1% Et_3N]) gave **24a** as a white solid (206 mg, 74%). R_f = 0.27 (SiO_2; DCM:MeOH:Et_3N, 80:19:1); **^{1}H NMR** (400 MHz, DMSO-d_6) δ: 7.28 (d, J = 14.7 Hz, 1H), 6.49 (s, 2H), 6.28 (d, J = 8.0 Hz, 1H), 3.73 (s, 3H), 3.01–2.94 (m, 4H), 2.85–2.79 (m, 4H); **HRMS** *m/z* (ESI$^+$) found 254.1297, $C_{12}H_{17}FN_3O_2$ ([M+H]$^+$) requires 254.1299.

Methyl 2-amino-5-fluoro-4-(4-((4-(trifluoromethyl)phenyl)carbamothioyl)piperazin-1-yl)benzoate (24)

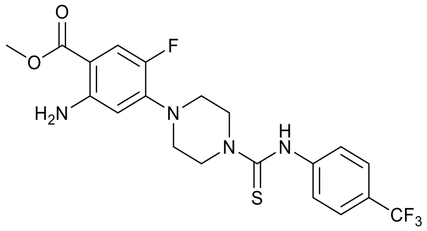

Prepared following *general procedure B* from **24a** (150 mg, 0.59 mmol) and 4-(trifluoromethyl)phenyl isothiocyanate. Purification by flash silica column chromatography (2–10% MeOH / DCM) gave **24** as a white solid (161 mg, 60%). R_f = 0.31 (SiO_2; DCM:MeOH, 98:2); **^{1}H NMR** (400 MHz, DMSO-d_6) δ: 9.69 (s, 1H), 7.66 (d, J = 8.6 Hz, 2H), 7.58 (d, J = 8.2 Hz, 2H), 7.34 (d, J = 14.5 Hz, 1H), 6.53 (s, 2H), 6.33 (d, J = 7.9 Hz, 1H), 4.07 (t, J = 5.0 Hz, 4H), 3.75 (s, 3H), 3.21 (t, J = 4.9 Hz, 4H); **HRMS** *m/z* (ESI$^+$) found 457.1323, $C_{20}H_{21}F_4N_4O_2S$ ([M+H]$^+$) requires 457.1316.

(3-Fluoro-4-(piperazin-1-yl)phenyl)(phenyl)methanone (25a)

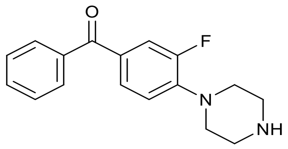

Prepared following *general procedure E* from 3,4-difluorobenzophenone (330 mg, 1.5 mmol) and piperazine, to give **25a** as a white solid (405 mg, 95%). **^{1}H NMR** (400 MHz, DMSO-d_6) δ: 7.71–7.63 (m, 3H), 7.59–7.44 (m, 4H), 7.10 (t, J = 8.8 Hz, 1H), 3.14–3.08 (m, 4H), 2.88–2.82 (m, 4H).

4-(4-Benzoyl-2-fluorophenyl)-*N* -(4-(trifluoromethyl)phenyl)piperazine-1-carbothioamide (25)

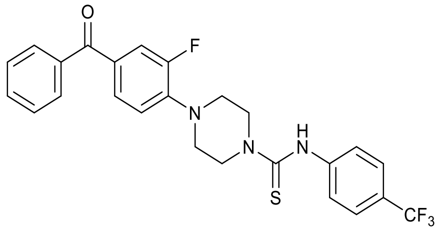

Prepared following *general procedure C* from **25a** (410 mg, 1.4 mmol) and 4-(trifluoromethyl)phenyl isothiocyanate. Purification by flash silica column chromatography (0–70% EtOAc/petroleum ether) gave **25** as a white solid (637 mg, 89%). R_f = 0.55 (SiO_2; Petroleum ether:EtOAc, 1:1); **^{1}H NMR** (400 MHz, DMSO-d_6) δ: 9.70 (s, 1H), 7.73–7.61 (m, 5H), 7.61–7.49 (m, 6H), 7.17 (t, *J* = 8.6 Hz, 1H), 4.10 (t, *J* = 5.0 Hz, 4H), 3.39–3.32 (m, 4H); **LRMS** *m/z* (ESI^-) 486 ($[M-H]^-$); **HRMS** *m/z* (ESI^-) found 486.1269, $C_{25}H_{20}F_4N_3OS$ ($[M-H]^-$) requires 486.1258.

6-Fluoro-7-(piperazin-1-yl)quinolin-4(1H)-one (26b)

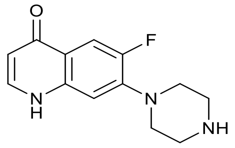

Step 1: 3,4-Difluoroaniline (0.15 mL, 1.6 mmol, 1.0 eq) and 5-methoxymethylene-2,2-dimethyl-[1,3]-dioxane-4,6-dione (292 mg, 1.6 mmol, 1.0 eq) were dissolved in 2-propanol (5 mL), and the solution was stirred at 70 °C for 30 min. The reaction mixture was filtered, and the residue was washed with MeOH and petroleum ether, and dried to obtain a yellow solid.[12] The solid was combined with biphenyl (5.8 g) and diphenyl ether (20 mL) and the suspension was stirred at 220 °C for 1 h. The reaction mixture was filtered and washed with DCM to obtain a pale-yellow solid (218 mg, 57%). This intermediate (**26a**) was used in the next step without further purification.

Step 2: Prepared following *general procedure E* from intermediate **26a** (200 mg, 1.1 mmol) and piperazine. Purification by flash silica column chromatography (5–20% MeOH/DCM [+1% Et_3N]) gave **26b** as a pale-yellow solid (185 mg, 68%). R_f = 0.30 (SiO_2; DCM:MeOH:Et_3N, 75:24:1); **^{1}H NMR** (400 MHz, DMSO-d_6) δ: 7.83 (d, *J* = 7.2 Hz, 1H), 7.62 (d, *J* = 7.6 Hz, 1H), 6.98 (d, *J* = 7.8 Hz, 1H), 5.93 (d, *J* = 7.2 Hz, 1H), 3.07–3.00 (m, 4H), 2.90–2.84 (m, 4H); **HRMS** *m/z* (ESI^+) found 248.1191, $C_{13}H_{15}FN_3O$ ($[M+H]^+$) requires 248.1194.

4-(6-Fluoro-4-oxo-1,4-dihydroquinolin-7-yl)-N-(4-(trifluoromethyl)phenyl)piperazine-1-carbothioamide (26)

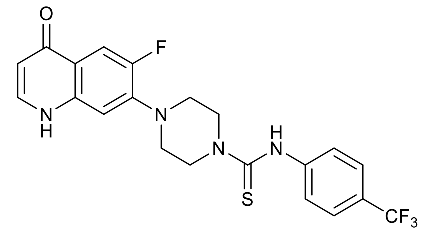

Prepared following *general procedure B* from **26b** (100 mg, 0.40 mmol) and 4-(trifluoromethyl)phenyl isothiocyanate. Purification by flash silica column chromatography (5–20% MeOH / DCM) gave **26** as a pale-yellow solid (137 mg, 75%). R_f = 0.28 (SiO_2; DCM:MeOH, 95:5); **^{1}H NMR** (400 MHz, DMSO-d_6) δ: 12.15 (br s, 1H), 9.77 (s, 1H), 7.98 (d, *J* = 7.3 Hz, 1H), 7.72 (d, *J* = 13.4 Hz, 1H), 7.67 (d, *J* = 8.5 Hz, 2H), 7.59 (d, *J* = 8.4 Hz, 2H), 7.10 (d, *J* = 7.5 Hz, 1H), 6.13 (d, *J* = 7.3 Hz, 1H), 4.14 (t, *J* = 5.1 Hz, 4H), 3.31 (t, *J* = 5.0 Hz, 4H); **^{13}C NMR** (101 MHz, DMSO-d_6) δ: 181.8, 175.5 (d, *J* = 2.2 Hz), 152.5 (d, *J* = 245 Hz), 145.3, 144.0 (d, *J* = 11.3 Hz), 140.0, 138.3, 125.6 (q, *J* = 3.6 Hz), 124.9 (q, *J* = 271 Hz), 124.7, 124.2 (q, *J* = 31.8 Hz), 120.3 (d, *J* = 6.5 Hz), 110.3 (d, *J* = 21.8 Hz), 107.9, 107.0 (d, *J* = 2.3 Hz), 49.6 (d, *J* = 3.8 Hz), 48.4; **HRMS** *m/z* (ESI$^+$) found 451.1210, $C_{21}H_{19}F_4N_4OS$ ($[M+H]^+$) requires 451.1210.

6,7-Difluoroquinazolin-4(1H)-one (27a)

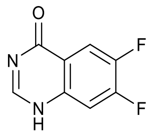

2-Amino-4,5-difluorobenzoic acid (1.0 g, 5.8 mmol), glacial acetic acid (2.5 mL), and formamide (9 mL) were mixed at 125 °C for 24 h. The mixture was cooled to RT and ice-cold H_2O (15 mL) was added. The precipitate was filtered and dried under vacuum to obtain **27a** as an off-white solid (950 mg, 90%). R_f = 0.21 (SiO_2; Petroleum ether:EtOAc, 1:1); **^{1}H NMR** (400 MHz, DMSO-d_6) δ: 12.48 (s, 1H), 8.14 (s, 1H), 8.02 (dd, *J* = 10.5, 8.8 Hz, 1H), 7.74 (dd, *J* = 11.4, 7.3 Hz, 1H). **HRMS** *m/z* (ESI$^+$) found 183.0360, $C_8H_4F_2N_2O$ ($[M+H]^+$) requires 183.0364. These data are in agreement with the literature.[13]

6-Fluoro-7-(piperazin-1-yl)quinazolin-4(1H)-one (27b)

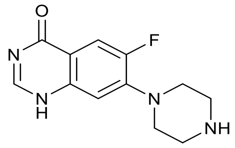

Prepared following *general procedure E* from **27a** (200 mg, 1.1 mmol) and piperazine. Purification by flash silica column chromatography (5–15% MeOH/DCM [+1% Et_3N]) gave **27b** as an off-white solid (273 mg, 89%). R_f = 0.25 (SiO_2; DCM:MeOH:Et_3N, 80:19:1); **^{1}H NMR** (400 MHz, DMSO-d_6) δ: 8.02 (s, 1H), 7.67 (d, J = 13.2 Hz, 1H), 7.10 (d, J = 8.1 Hz, 1H), 3.11 (s, 4H), 2.88 (s, 4H); **HRMS** m/z (ESI$^+$) found 249.1146, $C_{12}H_{14}FN_4O$ ([M+H]$^+$) requires 249.1146.

4-(6-Fluoro-4-oxo-1,4-dihydroquinazolin-7-yl)-N-(4-(trifluoromethyl)phenyl)piperazine-1-carbothioamide (27)

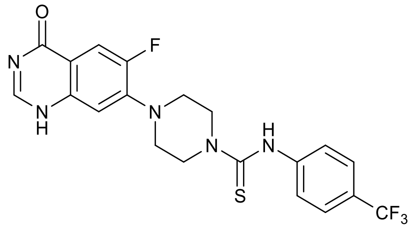

Prepared following *general procedure B* from **27b** (170 mg, 0.69 mmol) and 4-(trifluoromethyl)phenyl isothiocyanate. Purification by flash silica column chromatography (2–15% MeOH/DCM) gave **27** as a white solid (257 mg, 83%). R_f = 0.35 (SiO_2; DCM:MeOH, 95:5); **^{1}H NMR** (400 MHz, DMSO-d_6) δ: 12.19 (s, 1H), 9.79 (s, 1H), 8.04 (s, 1H), 7.72 (d, J = 12.9 Hz, 1H), 7.65 (d, J = 8.4 Hz, 2H), 7.59 (d, J = 8.4 Hz, 2H), 7.17 (d, J = 8.0 Hz, 1H), 4.12 (t, J = 5.0 Hz, 4H), 3.35 (t, J = 5.0 Hz, 4H); **^{13}C NMR** (101 MHz, DMSO-d_6) δ: 181.8, 160.2 (d, J = 2.9 Hz), 153.6 (d, J = 247 Hz), 147.4, 145.7, 145.5 (d, J = 9.8 Hz), 145.4, 125.6 (q, J = 3.8 Hz), 124.9 (q, J = 271 Hz), 124.7, 124.2 (q, J = 32.1 Hz), 116.5 (d, J = 8.2 Hz), 116.0 (d, J = 3.1 Hz), 111.8 (d, J = 23.0 Hz), 49.5 (d, J = 4.3 Hz), 48.4; **HRMS** m/z (ESI$^+$) found 452.1161, $C_{20}H_{18}F_4N_5OS$ ([M+H]$^+$) requires 452.1163.

Methyl 2-(6,7-difluoro-4-oxoquinazolin-3(4H)-yl)acetate (28a)

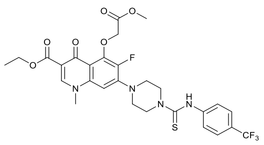

To a suspension of **27a** (182 mg, 1.0 mmol, 1.0 eq) in anhydrous DMF (10 mL), K_2CO_3 (207 mg, 1.5 mmol, 1.5 eq) and methyl 2-bromoacetate (100 µL, 1.1 mmol, 1.1 eq) were added. The reaction was heated at 50 °C for 2 h. The mixture was cooled, 5% w/v LiCl (30 mL) was added and extracted with EtOAc (3 × 25 mL). The organic extracts were combined, washed with H_2O (30 mL) and brine (30 mL), dried over Na_2SO_4, filtered, and concentrated *in vacuo* to give **28a** as a light brown solid (241 mg, 95%). R_f = 0.30 (SiO_2; Petroleum ether:EtOAc, 1:1); **^{1}H NMR** (400 MHz, DMSO-d_6) δ: 8.43 (s, 1H), 8.08 (dd, J = 10.4, 8.6 Hz, 1H), 7.83 (dd, J = 11.3, 7.3 Hz, 1H), 4.86 (s, 2H), 3.72 (s, 3H); **^{13}C NMR** (101 MHz, DMSO-d_6) δ: 168.7, 159.4 (d, J = 2.8 Hz), 154.4 (dd, J = 254, 14.6 Hz), 149.4 (dd, J = 249, 13.9 Hz), 149.3 (d, J = 2.2 Hz), 146.5 (dd, J = 11.4, 2.3 Hz), 119.0 (dd, J = 6.8, 1.8 Hz), 116.0 (d, J = 17.8 Hz), 114.2 (dd, J = 19.0, 2.1 Hz), 53.0, 47.7; **HRMS** *m/z* (ESI$^+$) found 255.0574, $C_{11}H_9F_2N_2O_3$ ([M+H]$^+$) requires 255.0576.

Methyl 2-(6-fluoro-4-oxo-7-(piperazin-1-yl)quinazolin-3(4H)-yl)acetate (28b)

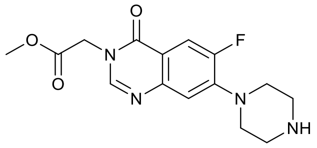

Prepared following *general procedure E* from **28a** (200 mg, 0.79 mmol) and piperazine. Purification by flash silica column chromatography (5–20% MeOH/DCM [+1% Et_3N]) gave **28b** as a white solid (190 mg, 75%). R_f = 0.21 (SiO_2; DCM:MeOH:Et_3N, 85:14:1); **^{1}H NMR** (400 MHz, DMSO-d_6) δ: 8.32 (s, 1H), 7.73 (d, J = 13.1 Hz, 1H), 7.20 (d, J = 8.0 Hz, 1H), 4.82 (s, 2H), 3.71 (s, 3H), 3.33–3.26 (m, 4H), 3.11–3.04 (m, 4H); **HRMS** *m/z* (ESI$^+$) found 321.1359, $C_{15}H_{18}FN_4O_3$ ([M+H]$^+$) requires 321.1357.

Methyl 2-(6-fluoro-4-oxo-7-(4-((4-(trifluoromethyl)phenyl)carbamothioyl)piperazin-1-yl)quinazolin-3(4H)-yl)acetate (28)

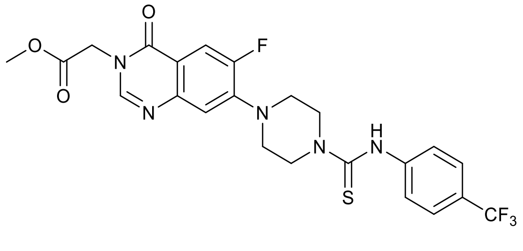

Prepared following *general procedure B* from **28b** (180 mg, 0.56 mmol) and 4-(trifluoromethyl)phenyl isothiocyanate. Purification by flash silica column chromatography (2–10% MeOH/DCM) gave **28** as a

white solid (44 mg, 15%). R_f = 0.30 (SiO_2; DCM:MeOH, 98:2); **^{1}H NMR** (400 MHz, DMSO-d_6) δ: 9.73 (s, 1H), 8.32 (s, 1H), 7.75 (d, *J* = 12.9 Hz, 1H), 7.66 (d, *J* = 8.4 Hz, 2H), 7.58 (d, *J* = 8.4 Hz, 2H), 7.21 (d, *J* = 7.9 Hz, 1H), 4.82 (s, 2H), 4.13 (t, *J* = 4.9 Hz, 4H), 3.71 (s, 3H)[3]; **^{13}C NMR** (101 MHz, DMSO-d_6) δ: 181.8, 168.9, 159.6 (d, *J* = 2.5 Hz), 153.8 (d, *J* = 248 Hz), 148.3, 146.6, 145.8 (d, *J* = 9.9 Hz), 145.3, 125.6 (q, *J* = 3.76 Hz), 125.0 (q, *J* = 271 Hz), 124.7, 124.3 (q, *J* = 31.9 Hz), 115.9 (d, *J* = 3.2 Hz), 115.0 (d, *J* = 8.5 Hz), 112.0 (d, *J* = 23.0 Hz), 52.9, 49.4 (d, *J* = 4.3 Hz), 48.3, 47.5; **HRMS** *m/z* (ESI$^+$) found 524.1373, $C_{23}H_{22}F_4N_5O_3S$ ([M+H]$^+$) requires 524.1374.

6,7-Difluoroquinazoline-2,4(1H,3H)-dione (29a)

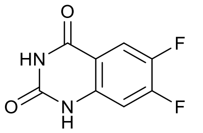

Potassium cyanate (423 mg, 5.2 mmol, 1.3 eq) was added to a solution of 2-amino-4,5-difluorobenzoic acid (700 mg, 4.04 mmol, 1.0 eq) in H_2O/AcOH (18 mL/0.25 mL). The mixture was stirred at RT for 18 h. Then, NaOH (0.092 mol) was slowly added and, after stirring for 10 min, the residue was filtered, resuspended in H_2O and acidified to pH 4 with 4 M HCl. The formed precipitate was filtered, washed with cold H_2O, and dried to give **29a** as an off-white solid (480 mg, 61%). R_f = 0.30 (SiO_2; DCM:MeOH:Et_3N, 90:9:1); **^{1}H NMR** (400 MHz, DMSO-d_6) δ: 11.48 (s, 1H), 11.27 (s, 1H), 7.88–7.78 (m, 1H), 7.15–7.05 (m, 1H). **HRMS** *m/z* (ESI$^+$) found 199.0316, $C_8H_4F_2N_2O_2$ ([M+H]$^+$) requires 199.0314. These data are in agreement with the literature.[14]

6-Fluoro-7-(piperazin-1-yl)quinazoline-2,4(1H,3H)-dione (29b)

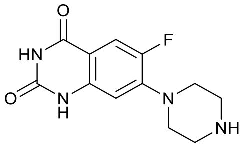

Prepared following *general procedure E* from **29a** (180 mg, 0.91 mmol) and piperazine. Purification by flash silica column chromatography (10–20% MeOH/DCM [+1% Et_3N]) gave **29b** as an off-white solid (145 mg, 60%). R_f = 0.35 (SiO_2; DCM:MeOH:Et_3N, 80:19:1); **^{1}H NMR** (400 MHz, DMSO-d_6) δ: 7.48 (d, *J* = 13.0 Hz, 1H), 6.68 (d, *J* = 7.3 Hz, 1H), 3.19–3.12 (m, 4H), 3.02–2.95 (m, 4H). **HRMS** *m/z* (ESI$^+$) found 265.1021, $C_{12}H_{14}FN_4O_2$ ([M+H]$^+$) requires 265.1022.

[3] Second piperazine 4H obscured by water peak.

4-(6-Fluoro-2,4-dioxo-1,2,3,4-tetrahydroquinazolin-7-yl)-N-(4-(trifluoromethyl)phenyl) piperazine-1-carbothioamide (29)

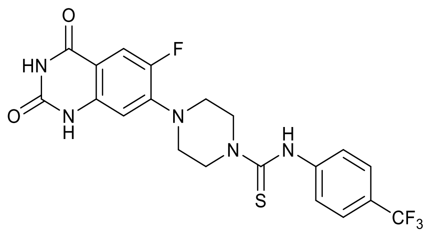

Prepared following *general procedure B* from **29b** (100 mg, 0.38 mmol) and 4-(trifluoromethyl)phenyl isothiocyanate. Purification by flash silica column chromatography (10–25% MeOH / DCM) gave **29** as an off-white solid (115 mg, 65%). R_f = 0.33 (SiO_2; DCM:MeOH, 95:5); **^{1}H NMR** (400 MHz, DMSO-d_6) δ: 11.18 (s, 1H), 11.02 (s, 1H), 9.71 (s, 1H), 7.66 (d, *J* = 8.6 Hz, 2H), 7.58 (d, *J* = 8.5 Hz, 2H), 7.51 (d, *J* = 12.9 Hz, 1H), 6.67 (d, *J* = 7.3 Hz, 1H), 4.10 (t, *J* = 4.8 Hz, 4H), 3.31 (t, *J* = 5.2 Hz, 4H); **^{13}C NMR** (101 MHz, DMSO-d_6) δ: 181.8, 162.4 (d, *J* = 2.5 Hz), 150.9, 150.4 (d, *J* = 241 Hz), 145.7 (d, *J* = 9.82 Hz), 145.3, 139.1, 125.6 (q, *J* = 4.1 Hz), 124.9 (q, *J* = 272 Hz), 124.7, 124.4 (q, *J* = 32.1 Hz), 113.2 (d, *J* = 23.5 Hz), 107.3 (d, *J* = 7.5 Hz), 104.2 (d, *J* = 2.1 Hz), 49.2 (d, *J* = 4.3 Hz), 48.3; **HRMS** *m/z* (ESI$^+$) found 468.1119, $C_{20}H_{18}F_4N_5O_2S$ ($[M+H]^+$) requires 468.1112.

Ethyl 2-(6,7-difluoro-2,4-dioxo-1,4-dihydroquinazolin-3(2H)-yl)acetate (30a)

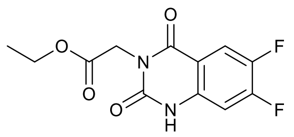

To a suspension of methyl-2-amino-4,5-difluorobenzoate (800 mg, 4.3 mmol, 1.0 eq) in pyridine (5 mL), ethyl isocyanatoacetate (0.74 mL, 6.6 mmol, 1.5 eq) was added dropwise. The reaction mixture was stirred at 50 °C for 5 h, then allowed to cool to RT. The solvent was removed *in vacuo* and the residue resuspended in EtOH. NaOEt 21% *w/w* in EtOH (3.2 mL, 2 eq of NaOEt) was added. After stirring for 1 h at RT, the mixture was slowly neutralized with 2 M HCl at 0 °C. The volatiles were removed *in vacuo* and the resulting solid was collected by filtration, washed with H_2O and EtOH and dried *in vacuo* to obtain **30a** as an orange solid (1.21 g, 91%). R_f = 0.35 (SiO_2; DCM:MeOH, 98:2); **^{1}H NMR** (400 MHz, DMSO-d_6) δ: 11.76 (s, 1H), 7.92 (dd, *J* = 10.2, 8.4 Hz, 1H), 7.17 (dd, *J* = 10.9, 6.6 Hz, 1H), 4.63 (s, 2H), 4.15 (q, *J* = 7.1 Hz, 2H), 1.20 (t, *J* = 7.1 Hz, 3H); **^{13}C NMR** (101 MHz, DMSO-d_6) δ: 168.3, 160.7 (d, *J* = 2.4 Hz), 154.5 (dd, *J* = 255, 14.6 Hz), 150.0, 146.2 (dd, *J* = 244, 13.9 Hz), 137.5 (d,

J = 11.2 Hz), 116.2 (d, J = 19.2 Hz), 110.5 (d, J = 4.2 Hz), 104.8 (d, J = 21.9 Hz), 61.6, 41.9, 14.5; **HRMS** *m/z* (ESI^+) found 285.0677, $C_{12}H_{11}F_2N_2O_4$ ($[M+H]^+$) requires 285.0681.

Ethyl 2-(6-fluoro-2,4-dioxo-7-(piperazin-1-yl)-1,4-dihydroquinazolin-3(2H)-yl)acetate (30b)

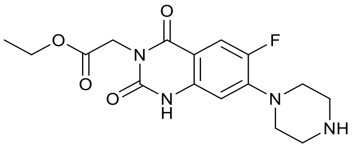

Prepared following *general procedure E* from **30a** (200 mg, 0.81 mmol) and piperazine. Purification by flash silica column chromatography (0–15% MeOH/DCM [+1% Et_3N]) gave **30b** as a white solid (261 mg, 92%). R_f = 0.25 (SiO_2; DCM:MeOH:Et_3N, 90:9:1); **^{1}H NMR** (400 MHz, DMSO-d_6) δ: 7.54 (d, J = 13.0 Hz, 1H), 6.71 (d, J = 7.3 Hz, 1H), 4.61 (s, 2H), 4.13 (q, J = 7.1 Hz, 2H), 3.23–3.17 (m, 4H), 3.04–2.97 (m, 4H), 1.20 (t, J = 7.1 Hz, 3H); **HRMS** *m/z* (ESI^+) found 351.1478, $C_{16}H_{20}FN_4O_4$ ($[M+H]^+$) requires 351.1463.

Ethyl 2-(6-fluoro-2,4-dioxo-7-(4-((4-(trifluoromethyl)phenyl)carbamothioyl)piperazin-1-yl)-1,4-dihydroquinazolin-3(2H)-yl)acetate (30)

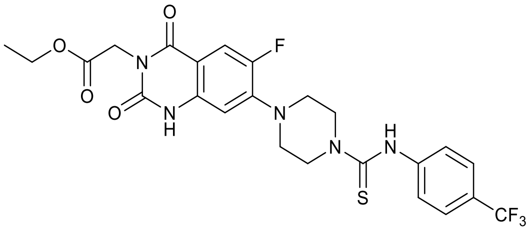

Prepared following *general procedure B* from **30b** (200 mg, 0.57 mmol) and 4-(trifluoromethyl)phenyl isothiocyanate. Purification by flash silica column chromatography (2–15% MeOH / DCM) gave **30** as a white solid (173 mg, 55%). R_f = 0.32 (SiO_2; DCM:MeOH, 95:5); **^{1}H NMR** (400 MHz, DMSO-d_6) δ: 11.52 (br s, 1H), 9.72 (br s, 1H), 7.66 (d, J = 8.3 Hz, 2H), 7.62–7.53 (m, 3H), 6.69 (d, J = 7.2 Hz, 1H), 4.62 (s, 2H), 4.19–4.08 (m, 6H), 1.20 (t, J = 7.1 Hz, 3H)[4]; **^{13}C NMR** (101 MHz, DMSO-d_6) δ: 181.8, 168.5, 161.0 (d, J = 2.4 Hz), 150.5 (d, J = 242 Hz), 150.3, 146.0 (d, J = 9.9 Hz), 145.3, 137.7, 125.6 (q, J = 3.8 Hz), 124.9 (q, J = 271 Hz), 124.7, 124.3 (q, J = 31.7 Hz), 113.7 (d, J = 23.5 Hz), 106.1 (d, J = 7.9 Hz), 104.0, 61.5, 49.1 (d, J = 4.3 Hz), 48.2, 41.7, 14.5; **HRMS** *m/z* (ESI^+) found 554.1476, $C_{24}H_{24}F_4N_5O_4S$ ($[M+H]^+$) requires 554.1480.

[4] Second piperazine 4H obscured by water peak.

2-(6-Fluoro-2,4-dioxo-7-(4-((4-(trifluoromethyl)phenyl)carbamothioyl)piperazin-1-yl)-1,4-dihydroquinazolin-3(2H)-yl)acetic acid (31)

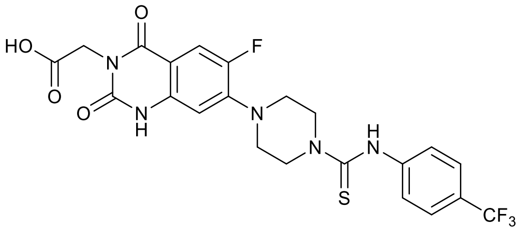

Step 1: LiOH (60 mg, 1.4 mmol, 2.5 eq) was added to a solution of **30b** (200 mg, 0.57 mmol, 1.0 eq) in H_2O (10 mL). After 30 min at RT, the mixture was slowly neutralized to pH 6 with 2 M HCl and filtered under vacuum to afford a white solid (170 mg, 92%). This intermediate (**31a**) was used in the next step without further purification.

Step 2: Prepared following *general procedure B* from intermediate **31a** (180 mg, 0.57 mmol) and 4-(trifluoromethyl)phenyl isothiocyanate. Purification by flash silica column chromatography (0–10% MeOH / DCM [+0.5% formic acid]) gave **31** as a white solid (245 mg, 82%). R_f = 0.22 (SiO_2; DCM:MeOH:formic acid, 94.5:5:0.5); **^{1}H NMR** (400 MHz, DMSO-d_6) δ: 12.89 (br s, 1H), 11.50 (s, 1H), 9.78 (s, 1H), 7.66 (d, *J* = 8.6 Hz, 2H), 7.62–7.54 (m, 3H), 6.72 (d, *J* = 7.3 Hz, 1H), 4.53 (s, 2H), 4.12 (t, *J* = 4.8 Hz, 4H)[5] **^{13}C NMR** (101 MHz, DMSO-d_6 δ:181.8, 169.9, 161.1 (d, *J* = 2.0 Hz), 150.5 (d, *J* = 242 Hz), 150.4, 145.9 (d, *J* = 9.8 Hz), 145.4, 137.7, 125.6 (q, *J* = 3.9 Hz), 124.9 (q, *J* = 271 Hz), 124.7, 124.2 (q, *J* = 31.9 Hz), 113.6 (d, *J* = 23.5 Hz), 106.3 (d, *J* = 8.2 Hz), 104.0, 49.1 (d, *J* = 4.4 Hz), 48.3, 41.8; **HRMS** *m/z* (ESI$^-$) found 524.1022, $C_{22}H_{18}F_4N_5O_4S$ ([M−H]$^-$) requires 524.1021.

Ethyl 2-(1-ethyl-6,7-difluoro-2,4-dioxo-1,4-dihydroquinazolin-3(2H)-yl)acetate (32a)

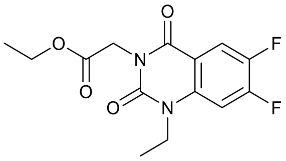

Bromoethane (210 µL, 2.8 mmol, 3.0 eq) was added dropwise to a suspension of **30a** (265 mg, 0.93 mmol, 1.0 eq) and K_2CO_3 (260 mg, 1.9 mmol, 2.0 eq) in DMF (10 mL). The reaction was stirred for

[5] Second piperazine 4H obscured by water peak.

18 h at RT, before being quenched by H_2O (20 mL). The organic components were extracted with EtOAc (3 × 30 mL), washed with H_2O (30 mL) and brine (30 mL), dried over Na_2SO4, filtered and concentrated *in vacuo*, to give **32a** as a pale yellow oil (266 mg, 91%). R_f = 0.71 (SiO_2; Et_2O); **^{1}H NMR** (400 MHz, $CDCl_3$) δ: 8.02 (dd, *J* = 9.6, 8.6 Hz, 1H), 7.04 (dd, *J* = 11.4, 6.1 Hz, 1H), 4.80 (s, 2H), 4.23 (q, *J* = 7.2 Hz, 2H), 4.13 (q, *J* = 7.2 Hz, 2H), 1.35 (t, *J* = 7.2 Hz, 3H), 1.29 (t, *J* = 7.2 Hz, 3H); **LRMS** *m/z* (ESI^+) 335 ($[M+Na]^+$).

Ethyl 2-(1-ethyl-6-fluoro-2,4-dioxo-7-(piperazin-1-yl)-1,4-dihydroquinazolin-3(2H)-yl)acetate (32b)

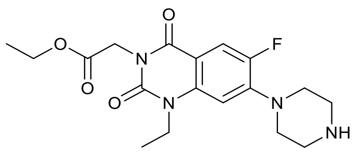

Prepared following *general procedure E* from **32a** (265 mg, 0.85 mmol) and piperazine. Purification by flash silica column chromatography (0–100% EtOAC/12:2:1 EtOAc:EtOH:NH_4OH) gave **32b** as a yellow solid (307 mg, 96%). R_f = 0.11 (SiO_2; DCM:MeOH, 95:5); **^{1}H NMR** (400 MHz, $CDCl_3$) δ: 7.78 (d, *J* = 12.6 Hz, 1H), 6.55 (d, *J* = 6.8 Hz, 1H), 4.79 (s, 2H), 4.22 (q, *J* = 7.2 Hz, 2H), 4.15 (q, *J* = 7.2 Hz, 2H), 3.33–3.25 (m, 4H), 3.17–3.09 (m, 4H), 1.34 (t, *J* = 7.2 Hz, 3H), 1.28 (t, *J* = 7.2 Hz, 3H); **LRMS** *m/z* (ESI^+) 379 ($[M+H]^+$).

2-(1-Ethyl-6-fluoro-2,4-dioxo-7-(4-((4-(trifluoromethyl)phenyl)carbamothioyl)piperazin-1-yl)-1,4-dihydroquinazolin-3(2H)-yl)acetic acid (32)

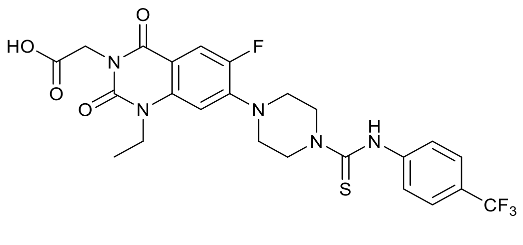

Step 1: LiOH (135 mg, 3.2 mmol, 4.0 eq) was added to a solution of **32b** (305 mg, 0.81 mmol, 1.0 eq) in 2:1 THF/H_2O (9 mL). The reaction was stirred at RT for 3 h, and the THF removed *in vacuo*. The solution was acidified by the addition of 1 M HCl to pH 2 and filtered under vacuum to afford a white solid (241 mg, 85%) . R_f = 0.00 (SiO_2; DCM:MeOH, 90:10); **LRMS** *m/z* (ESI^+) 351 ($[M+H]^+$). This intermediate (**32c**) was used in the next step without further purification.

Step 2: Prepared following *general procedure B* from intermediate **32c** (100 mg, 0.29 mmol) and 4(trifluoromethyl)phenyl isothiocyanate. Purification by flash silica column chromatography (0–10% MeOH/DCM [+0.5% formic acid]) gave **32** as a white solid (102 mg, 65%). R_f = 0.53 (SiO_2; DCM:MeOH:formic acid, 90:9.5:0.5); **^{1}H NMR** (600 MHz, DMSO-d$_6$) δ: 9.73 (s, 1H), 7.68 (d, *J* = 12.7 Hz, 2H), 7.66 (d, *J* = 8.4 Hz, 3H), 7.58 (d, *J* = 8.3 Hz, 2H), 6.82 (d, *J* = 6.9 Hz, 1H), 4.57 (s, 2H), 4.18 (q, *J* = 7.1 Hz, 3H), 4.16–4.10 (t, *J* = 5.2 Hz, 4H), 3.47–3.41 (t, *J* = 5.2 Hz, 4H), 1.23 (t, *J* = 7.1 Hz, 3H); **^{13}C NMR** (151 MHz, DMSO-*d$_6$*) δ: 181.2, 169.3, 159.6, 149.9 (d, *J* = 243 Hz), 149.8, 145.54 (d, *J* = 9.5 Hz), 144.85, 137.09, 125.2 (q, *J* = 3.6 Hz), 124.5 (q, *J* = 271 Hz), 142.4, 123.8 (q, *J* = 31.9 Hz), 114.0 (d, *J* = 23.1 Hz), 107.06 (d, *J* = 6.7 Hz), 103.46, 48.83 (d, *J* = 4.5 Hz), 47.83, 42.35, 12.41; **LRMS** *m/z* (ESI$^+$) 554 ([M+H]$^+$); **HRMS** *m/z* (ESI$^+$) found 554.1500, $C_{25}H_{24}F_4N_5O_4S$ ([M+H]$^+$) requires 554.1480.

Ethyl 1-cyclopropyl-6-fluoro-4-oxo-7-(piperazin-1-yl)-1,4-dihydroquinoline-3-carboxylate (33a)

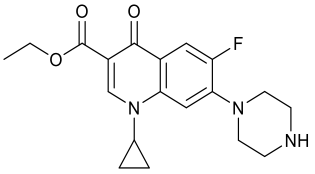

Prepared following *general procedure E* from **1b** (110 mg, 0.36 mmol) and piperazine. Purification by flash silica column chromatography (18–20% MeOH/DCM) gave **33a** as an off-white solid (95 mg, 70%). R_f = 0.30 (SiO_2; DCM:MeOH, 90.10); **^{1}H NMR** (400 MHz, $CDCl_3$) δ 8.52 (s, 1H), 8.03 (d, *J* = 13.3 Hz, 1H), 7.31–7.26 (m, 1H), 4.37 (q, *J* = 7.1 Hz, 2H), 3.43 (tt, *J* = 7.2, 3.9 Hz, 1H), 3.30–3.23 (m, 4H), 3.15 – 3.08 (m, 4H), 1.40 (t, *J* = 7.1 Hz, 3H), 1.39–1.27 (m, 2H), 1.18–1.09 (m, 2H)); **LRMS** *m/z* (ESI$^+$) 360.2 ([M+H]$^+$). These data are in agreement with the literature.[7]

Ethyl 1-cyclopropyl-6-fluoro-4-oxo-7-(4-((4-(trifluoromethyl)phenyl)carbamothioyl)piperazin-1-yl)-1,4-dihydroquinoline-3-carboxylate (33)

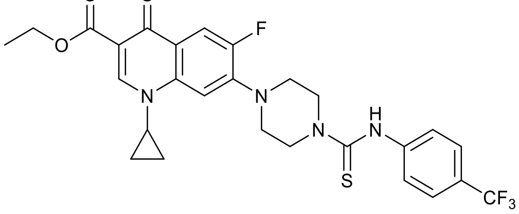

Prepared following *general procedure B* from **33a** (95 mg, 0.26 mmol) and 4-(trifluoromethyl)phenyl isothiocyanate. Purification by flash silica column chromatography (1–2% MeOH/DCM) gave **33** as a

light-yellow solid (80 mg, 54%). R_f = 0.6 (SiO_2; DCM:MeOH, 90.10); **^{1}H NMR** (400 MHz, DMF-d_7) δ 9.97 (s, 1H), 8.54 (s, 1H), 7.87 (d, *J* = 13.5 Hz, 1H), 7.75 (d, *J* = 8.7 Hz, 2H), 7.70 (d, *J* = 8.9 Hz, 2H), 7.60 (d, *J* = 7.4 Hz, 1H), 4.33–4.29 (m, 4H), 4.27 (q, *J* = 7.1 Hz, 2H) 3.75 (tt, *J* = 7.1, 4.0 Hz, 1H), 3.53–3.46 (m, 4H), 1.38 (td, *J* = 7.4, 5.3 Hz, 2H), 1.32 (t, *J* = 7.1 Hz, 3H), 1.27 – 1.21 (m, 2H); **HRMS** *m/z* (ESI^+) found 563.1735, $C_{27}H_{27}F_4N_4O_3S$ ($[M+H]^+$) requires 563.1735; **HPLC** Retention time 11.9 min, 97.7% (280 nm).

2-((*tert*-Butoxycarbonyl)amino)ethyl 1-cyclopropyl-6,7-difluoro-4-oxo- 1,4-dihydroquinoline-3-carboxylate (34a)

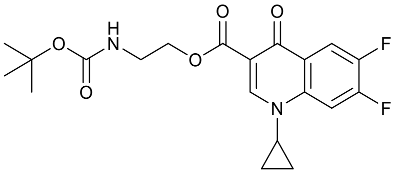

DIAD (300 µL, 1.5 mmol) was added slowly to a 0 °C suspension of **18a** (420 mg, 1.5 mmol, 1.5 eq), triphenyl phosphine (390 mg, 1.5 mmol, 1.5 eq), and *N*-Boc-ethanolamine (230 µL, 1.0 mmol, 1.0 eq) in anhydrous DMF. The reaction was allowed to warm to RT and stirred for 18 h. H_2O (30 mL) was added, the product was extracted with DCM (3 × 30 mL) and EtOAc (2 × 30 mL), dried, and the solvent was removed *in vacuo*. The residue was purified by flash silica column chromatography (0–10% MeOH/DCM), to give **34a** as an off-white solid (504 mg, 79%). **^{1}H NMR** (400 MHz, DMSO-d_6) δ: 8.55 (s, 1H), 8.13 (dd, *J* = 12.1, 6.7 Hz, 1H), 8.09–8.01 (m, 1H), 6.99 (t, *J* = 6.0 Hz, 1H), 4.14 (t, *J* = 5.6 Hz, 2H), 3.67–3.58 (m, 1H), 3.30–3.22 (m, 2H), 1.36 (s, 9H), 1.32–1.24 (m, 2H), 1.24–1.14 (m, 2H).

2-((*tert*-Butoxycarbonyl)amino)ethyl 1-cyclopropyl-6-fluoro-4-oxo-7-(piperazin-1-yl)-1,4-dihydroquinoline-3-carboxylate (34b)

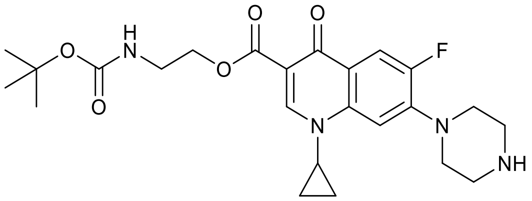

Prepared following *general procedure E* from **34a** (504 mg, 1.2 mmol) and piperazine, using DMF, to give **34b** as an off-white solid (954 mg, 96%). **^{1}H NMR** (400 MHz, DMSO-d_6) 8.48 (s, 1H), 7.73 (d, *J* = 13.5 Hz, 1H), 7.41 (d, *J* = 7.4 Hz, 1H), 6.99 (t, *J* = 5.9 Hz, 1H), 4.13 (t, *J* = 5.6 Hz, 2H), 3.65 (tt, *J* = 7.5, 4.1

Hz, 1H), 3.30–3.21 (m, 2H), 3.15 (dd, *J* = 6.3, 3.4 Hz, 4H), 2.92–2.83 (m, 4H), 1.36 (s, 9H), 1.28–1.21 (m, 2H), 1.10 (dt, *J* = 7.2, 3.9 Hz, 2H).

2-((*tert*-Butoxycarbonyl)amino)ethyl 1-cyclopropyl-6-fluoro-4-oxo-7- (4-((4-(trifluoromethyl)phenyl)carbamothioyl)piperazin-1-yl)-1,4-dihydroquinoline-3-carboxylate (34c)

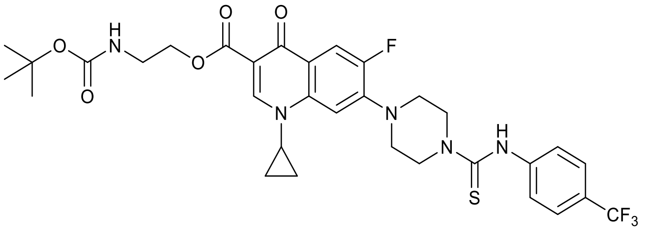

Prepared following *general procedure B* from **34b** (954 mg, 1.2 mmol) and 4-(trifluoromethyl) phenyl isothiocyanate (310 mg, 1.5 mmol). Purification by flash silica column chromatography (0–10% MeOH/DCM) gave **34c** as an off-white solid (500 mg, 49%). ^{1}**H NMR** (400 MHz, DMSO-d_6) δ: 9.73 (s, 1H), 8.50 (s, 1H), 7.79 (d, *J* = 13.2 Hz, 1H), 7.66–7.61 (m, 2H), 7.58 (dd, *J* = 9.2, 1.1 Hz, 2H), 7.48 (d, *J* = 7.4 Hz, 1H), 6.98 (t, *J* = 5.9 Hz, 1H), 4.21–4.08 (m, 6H), 3.66 (tt, *J* = 7.1, 3.9 Hz, 1H), 3.40 (dd, *J* = 7.2, 3.7 Hz, 4H), 3.30–3.21 (m, 2H), 1.37 (s, 9H), 1.26 (dd, *J* = 7.6, 5.6 Hz, 2H), 1.15–1.10 (m, 2H).

2-((1-Cyclopropyl-6-fluoro-4-oxo-7-(4-((4-(trifluoromethyl)phenyl) carbamothioyl) piperazin-1-yl)-1,4-dihydroquinoline-3-carbonyl)oxy) ethan-1-aminium chloride (34)

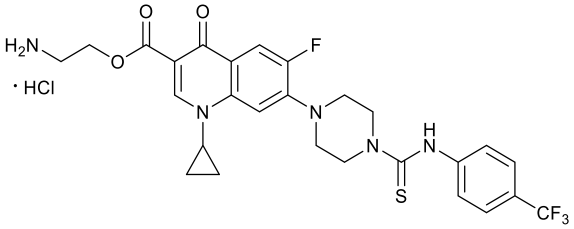

34c (500 mg, 0.73 mmol) was added to 4 M HCl in dioxane (5 mL), and DCM (4 mL) and stirred at RT for 18 h. The solvent was removed *in vacuo* and the residue washed with DCM, to give **34** as a pale-orange solid (304 mg, 53%). ^{1}**H NMR** (400 MHz, DMSO-d_6) δ: 9.88 (s, 1H), 8.61 (s, 1H), 7.82 (d, *J* = 13.3 Hz, 1H), 7.65 (d, *J* = 8.6 Hz, 2H), 7.60 (d, *J* = 8.6 Hz, 2H), 7.51 (d, *J* = 7.4 Hz, 1H), 4.36 (t, *J* = 5.1 Hz, 2H), 4.19–4.12 (m, 4H), 3.73–3.65 (m, 1H), 3.58 (t, *J* = 5.4 Hz, 3H), 2.90–2.80 (m, 2H), 1.32–1.28 (m, 2H),

1.19–1.11 (m, 2H)[6]; **LRMS** *m/z* (ESI+) 578 ([M+H]$^+$); **HRMS** *m/z* (ESI+) found 578.1858, $C_{27}H_{28}F_4N_5O_3S$ ([M+H]$^+$) requires 578.1844.

1-Cyclopropyl-6-fluoro-*N*-(2-hydroxyethyl)-4-oxo-7-(4-((4-(trifluoromethyl) phenyl)carbamothioyl)piperazin-1-yl)-1,4-dihydroquinoline-3-carboxamide (35)

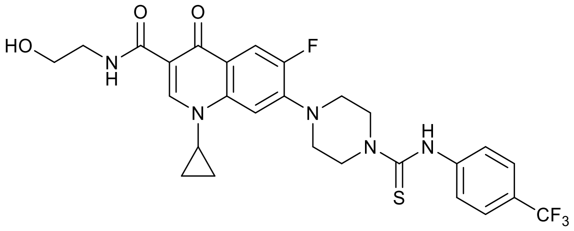

34 (180 mg, 0.29 mmol) was dissolved in MeOH (20 mL) and Et_3N (5 mL) was added. The solution was stirred for 30 min at RT. The solvent was removed *in vacuo* and the residue purified twice by flash silica column chromatography (0–10% MeOH/DCM), to give **35** as an off-white solid (64 mg, 38%). R_f = 0.60 (SiO_2; DCM:MeOH, 9:1); **^{1}H NMR** (400 MHz, DMSO-d_6) δ: 10.00 (t, *J* = 5.6 Hz, 1H), 9.73 (s, 1H), 8.62 (s, 1H), 7.85 (dd, *J* = 13.3, 1.9 Hz, 1H), 7.65 (d, *J* = 8.5 Hz, 2H), 7.57 (d, *J* = 8.4 Hz, 2H), 7.50 (d, *J* = 7.4 Hz, 1H), 4.84 (t, *J* = 5.1 Hz, 1H), 4.15 (t, *J* = 4.9 Hz, 4H), 3.71 (tt, *J* = 7.2, 4.0 Hz, 1H), 3.52 (q, *J* = 5.5 Hz, 2H), 3.38 (q, *J* = 5.5 Hz, 2H), 1.29 (dd, *J* = 7.5, 5.7 Hz, 2H), 1.20–1.05 (m, 2H)[2]; **HRMS** *m/z* (ESI$^-$) found 576.1701, $C_{27}H_{26}F_4N_5O_3S$ ([M−H]$^-$) requires 576.1698.

Ethyl (*z*)-2-(2-amino-4,5-difluorobenzoyl)-3-ethoxyacrylate (36a)

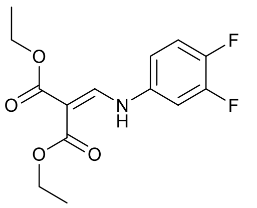

3,4-Difluoroalanine (1.0 g, 7.8 mmol) and diethylethyoxymethelyne malonate (1.7 g, 7.8 mmol) were stirred at 100 °C for 18 h. The mixture was cooled to RT, and the product recrystalized in EtOH to give **36a** as a colourless crystalline solid (2.0 g, 87%). R_f = 0.38 (SiO2; DCM); **^{1}H NMR** (400 MHz, CDCl3) δ: 11.00 (d, *J* = 12 Hz, 1H), 8.38 (d, *J* = 13.4 Hz, 1H), 7.19 (dt, *J* = 9.7, 8.6 Hz, 1H), 7.00 (ddd, *J* = 11.1, 6.6, 2.8 Hz, 1H), 6.87 (dtd, *J* = 8.9, 3.1, 1.6 Hz, 1H), 4.30 (dq, *J* = 21.5, 7.1 Hz, 4H), 1.37 (dt, *J* = 18.6, 7.1 Hz,

[6] Second piperazine 4H obscured by water peak.

6H); **LRMS** m/z (ESI$^+$) 300 ([M+H]$^+$); **HRMS** m/z (ESI$^+$) found 621.1842, $C_{28}H_{30}F_4N_2O_8Na$ ([2M+Na]$^+$) requires 621.1831. These data are in agreement with the literature.[17]

Ethyl 1-benzyl-6,7-difluoro-4-oxo-1,4-dihydroquinoline-3-carboxylate (36c)

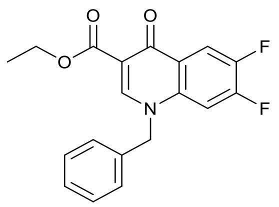

Step 1: **36a** (940 mg, 3.1 mmol) was refluxed in Ph_2O (10 mL) for 1 h. The mixture was cooled in an ice bath and the resultant solid collected by solid filtration and washed with Et_2O (20 mL) to give a white solid. This intermediate (**36b**) was used in the next step without further purification.

Step 2: Intermediate **36b** (200 mg, 0.79 mmol) and K_2CO_3 (140 mg, 1.6 mmol) were suspended in anhydrous DMF (2 mL) and benzyl bromide (170 mg, 1.6 mmol) was added. The reaction was stirred at RT for 16 h after which NH_4Cl (20 mL) was added, and the suspension was stirred for 10 min. The product was extracted with DCM (3 × 10 mL) and dried over Mg_2SO_4 to give **36c** as a yellow solid (140 mg, 76%) which was used without purification. R_f = 0.71 (SiO_2; DCM:MeOH, 95:5); **^{1}H NMR** (400 MHz, DMSO-d_6) δ 8.93 (s, 1H), 8.10 (dd, *J* = 10.7, 8.9 Hz, 1H), 7.84 (dd, *J* = 12.3, 6.6 Hz, 1H), 7.43–7.23 (m, 5H), 5.67 (s, 2H), 4.25 (q, *J* = 7.1 Hz, 2H), 1.32 (t, *J* = 7.1 Hz, 3H); **LRMS** m/z (ESI$^+$) 344 ([M+H]$^+$); **HRMS** m/z (ESI$^+$) found 344.1089, $C_{19}H_{16}F_2NO_3$ ([M+H]$^+$) requires 344.1093. These data are in agreement with the literature.[18]

1-Benzyl-6-fluoro-4-oxo-7-(piperazin-1-yl)-1,4-dihydroquinoline-3-carboxylic acid (36e)

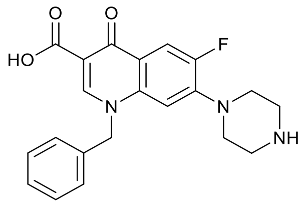

Step 1: **36c** (200 mg, 0.85 mmol) and piperazine (150 mg, 1.70 mmol) were stirred in pyridine (5 mL) at 70 °C for 18 h. The solvent was removed *in vacuo* and the product resuspended in H_2O (10 mL). The product was extracted in DCM (3 × 5 mL), dried over Mg_2SO_4, and concentrated to dryness to give a white solid. This intermediate (**36d**) was used in the next step without further purification.

Step 2: To intermediate **36d** (100 mg, 0.25 mmol) was added 1M NaOH solution (2 mL) and H_2O (5 mL). The solution was stirred for 16 h at 100 °C after which the solution acidified to pH 7 (1M HCl). The resultant solid was filtered and washed with H_2O (10 mL) and dried over Mg_2SO_4 to give **36e** as a white solid, 85 mg (91%). R_f = 0.00 (SiO_2; DCM:MeOH, 80:20); **^{1}H NMR** (400 MHz, DMSO-d_6) δ 9.21 (s, 1H), 7.88 (d, J = 13.4 Hz, 1H), 7.43–7.29 (m, 6H), 7.04 (d, J = 7.2 Hz, 1H), 5.85 (s, 2H), 3.04 (br s, 4H), 2.80 (br s, 4H); **LRMS** m/z (ESI$^+$) 382 ([M+H]$^+$); **HRMS** m/z (ESI$^+$) found 382.1579, $C_{21}H_{21}FN_3O_3$ ([M+H]$^+$) requires 382.1562. These data are in agreement with the literature.[18]

1-Benzyl-6-fluoro-4-oxo-7-(4-((4-(trifluoromethyl)phenyl)carbamothioyl)piperazin-1-yl)-1,4-dihydroquinoline-3-carboxylic acid (36)

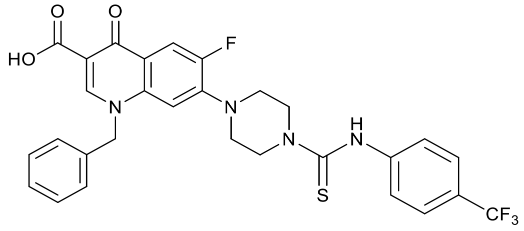

Prepared following *general procedure B* from **36e** (200 mg, 0.36 mmol) and 4-(trifluoromethyl)phenyl isocyanate. Purification by flash column chromatography DCM:MeOH (0-10%) gave **36** as a white solid (41 mg, 38%). R_f = 0.77 (SiO_2; DCM:MeOH, 9:1); **^{1}H NMR** (400 MHz, $CDCl_3$) δ 14.95 (s, 1H), 9.73 (s, 1H), 9.22 (s, 1H), 7.67 (d, J = 12.9 Hz, 1H), 7.58 (d, J = 8.3 Hz, 2H), 7.42 –7.31 (m, 5H), 7.13 (d, J = 8.3 Hz, 2H), 5.87 (s, 2H), 4.08 (br s, 4H)[7]; **LRMS** m/z (ESI$^+$) 585 ([M+H]$^+$); **HRMS** m/z (ESI$^+$) found 585.1591, $C_{29}H_{25}F_4N_4O_3S$ ([M+H]$^+$), requires 585.1578.

Ethyl 6,7-difluoro-1-(4-fluorobenzyl)-4-oxo-1,4-dihydroquinoline-3-carboxylate (37a)

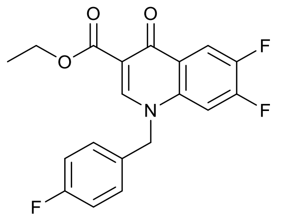

[7] Second piperazine 4H obscured by water peak.

Intermediate **36b** (250 mg, 1.0 mmol, 1.0 eq) and K_2CO_3 (272 mg, 2.0 mmol, 2.0 eq) were suspended in DMF (20 mL) and 4-fluorobenzyl bromide (250 µL, 2.0 mmol, 2.0 eq) was added dropwise. The reaction was stirred for 22 h at RT, before being quenched with H_2O (20 mL). The organic components were extracted with DCM (3 × 30 mL), washed with H_2O (30 mL) and brine (30 mL), dried over Na_2SO_4, and filtered. Purification by flash silica column chromatography (0–10% MeOH/DCM) gave **37a** as an off-white solid (270 mg, 90%). R_f = 0.43 (SiO_2; DCM:MeOH, 95:5); **^{1}H NMR** (400 MHz, $CDCl_3$) δ: 8.57 (s, 1H), 8.30 (dd, *J* = 10.4, 8.8 Hz, 1H), 7.17–7.05 (m, 4H), 5.31 (s, 2H), 4.41 (q, *J* = 7.1 Hz, 2H), 1.41 (t, *J* = 7.1 Hz, 3H); **LRMS** *m/z* (ESI^+) 362 ($[M+H]^+$). These data are in agreement with the literature.[18]

Ethyl 6-fluoro-1-(4-fluorobenzyl)-4-oxo-7-(piperazin-1-yl)-1,4-dihydroquinoline-3-carboxylate (37b)

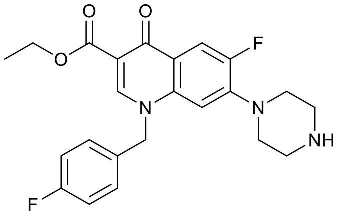

Prepared following *general procedure E* from **37a** (165 mg, 0.46 mmol) and piperazine. Purification by flash silica column chromatography (0–100% EtOAC/12:2:1 EtOAc:EtOH:NH_4OH) gave **37b** as a yellow solid (190 mg, 97%). R_f = 0.19 (SiO_2; EtOAc:EtOH:NH_4OH, 12:2:1); **^{1}H NMR** (400 MHz, $CDCl_3$) δ: 8.49 (s, 1H), 8.00 (d, *J* = 13.3 Hz, 1H), 7.18–7.13 (m, 2H), 7.08–7.02 (m, 2H), 6.53 (d, *J* = 6.9 Hz, 1H), 5.29 (s, 1H), 4.38 (q, *J* = 7.1 Hz, 2H), 3.02–2.95 (m, 8H), 1.40 (t, *J* = 7.1 Hz, 3H); **LRMS** *m/z* (ESI^+) 428 ($[M+H]^+$). These data are in agreement with the literature.[18]

Ethyl 6-fluoro-1-(4-fluorobenzyl)-4-oxo-7-(piperazin-1-yl)-1,4-dihydroquinoline-3-carboxylate (37c)

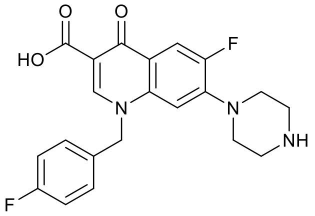

37b (132 mg, 0.31 mmol, 1.0 eq) was suspended in 1 M NaOH (10 mL) and heated at 90 °C for 16 h. The solution was neutralised to pH 7 by the addition of 1 M HCl. Precipitation at 0 °C followed by filtration gave **37c** as an off-white solid (85 mg, 69%). R_f = 0.01 (SiO_2; EtOAc:EtOH:NH_4OH, 12:2:1); **^{1}H NMR** (400 MHz, DMSO-*d_6*) δ: 9.00 (s, 1H), 7.83 (d, *J* = 13.5 Hz, 1H), 7.37–7.30 (m, 2H), 7.24–7.16 (m,

2H), 6.95 (d, *J* = 7.4 Hz, 1H), 5.71 (s, 2H), 3.03–2.96 (m, 4H), 2.82–2.75 (m, 4H); **LRMS** *m/z* (ESI^+) 400 ($[M+H]^+$). These data are in agreement with the literature.[18]

Ethyl 6-fluoro-1-(4-fluorobenzyl)-4-oxo-7-(piperazin-1-yl)-1,4-dihydroquinoline-3-carboxylate (37)

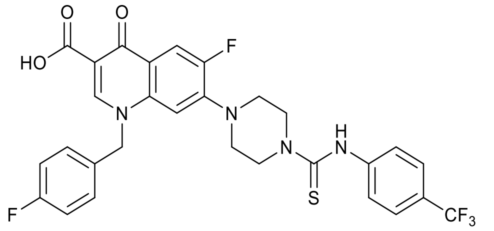

Prepared following *general procedure B* from **37c** (70 mg, 0.18 mmol) and 4-(trifluoromethyl)phenyl isothiocyanate. Purification by flash silica column chromatography (0–15% MeOH/EtOAc) gave **37** as an off-white solid (12 mg, 11%). R_f = 0.19 (SiO_2; DCM:MeOH, 95:5); ^{1}**H NMR** (400 MHz, DMSO-d_6) δ: 15.20 (br s, 1H), 9.88 (br s, 1H), 9.21 (s, 1H), 7.93 (d, *J* = 13.3 Hz, 1H) , 7.65 (d, *J* = 8.6 Hz, 2H), 7.60 (d, *J* = 8.6 Hz, 2H), 7.45–7.38 (m, 2H), 7.26–7.19 (m, 2H), 7.13 (d, *J* = 7.5 Hz, 1H), 5.85 (s, 2H), 4.15–4.05 (m, 4H)[8]; **LRMS** *m/z* (ESI^+) 603 ($[M+H]^+$); **HRMS** *m/z* (ESI^+) found 603.1495, $C_{29}H_{24}F_5N_4O_3S$ ($[M+H]^+$) requires 603.1484.

Ethyl 1-ethyl-6,7-difluoro-4-oxo-1,4-dihydroquinoline-3-carboxylate (38a)

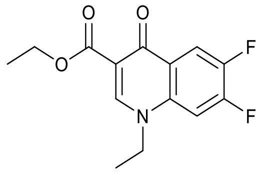

Prepared following *general procedure F* from **1a** (600 mg, 2.0 mmol) and ethylamine. Purification by flash silica column chromatography (60–75% EtOAc/petroleum ether) gave **38a** as yellow solid (270 mg, 48%). R_f = 0.20 (SiO_2; Petroleum ether:EtOAc, 50:50); ^{1}**H NMR** (400 MHz, $CDCl_3$) δ 8.41 (s, 1H), 8.23 (dd, *J* = 10.5, 8.8 Hz, 1H), 7.20 (dd, *J* = 11.2, 6.1 Hz, 1H), 4.32 (q, *J* = 7.1 Hz, 2H), 4.14 (q, *J* = 7.3 Hz, 2H), 1.49 (t, *J* = 7.3 Hz, 3H), 1.34 (t, *J* = 7.1 Hz, 3H). These data are in agreement with the literature.[15]

1-Ethyl-6,7-difluoro-4-oxo-1,4-dihydroquinoline-3-carboxylic acid (38b)

[8] Second piperazine 4H obscured by water peak.

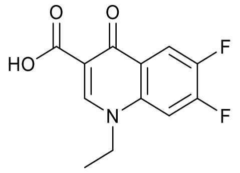

A solution of **38a** (200 mg, 0.71 mmol) in 1 M NaOH (10 mL) was heated to 80 °C for 16 h. The reaction mixture was cooled and acidified with 1 M HCl (pH 2–3), the precipitated solid was filtered, dried and triturated with EtOAc (2 × 10 mL) to afford **38b** as off white solid (160 mg, 89%). R_f = 0.10 (SiO_2; DCM:MeOH, 90.10); **^{1}H NMR** (400 MHz, DMSO-d_6) δ 14.91 (s, 1H), 9.09 (s, 1H), 8.37 – 8.24 (m, 1H), 7.28 (d, *J* = 7.3 Hz, 1H), 4.58 (q, *J* = 7.1 Hz, 2H), 1.40 (t, *J* = 7.1 Hz, 3H). These data are in agreement with the literature.[15]

1-Ethyl-6-fluoro-4-oxo-7-(piperazin-1-yl)-1,4-dihydroquinoline-3-carboxylic acid (38c)

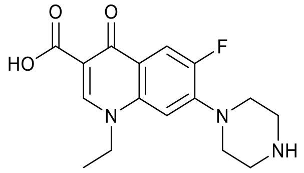

Prepared following *general procedure E* from **38b** (160 mg, 0.63 mmol) and piperazine. Purification by trituration with DCM (2 × 5 mL) gave **38c** as the crude white solid (160 mg, 80%). R_f = 0.00 (SiO_2; DCM:MeOH, 85.15); **^{1}H NMR** (400 MHz, DMSO-d_6) δ 8.94 (s, 1H), 7.91 (d, *J* = 13.4 Hz, 1H), 7.17 (d, *J* = 7.3 Hz, 1H), 4.59 (q, *J* = 7.1 Hz, 2H), 3.30 (t, *J* = 4.8 Hz, 4H), 2.98 (t, *J* = 4.8 Hz, 4H), 1.41 (t, *J* = 7.0 Hz, 3H); **LRMS** *m/z* (ESI$^+$) 320 ([M+H]$^+$), 352.3 (M+ CH_3OH+H]$^+$. These data are in agreement with the literature.[15]

1-Ethyl-6-fluoro-4-oxo-7-(4-((4-(trifluoromethyl)phenyl)carbamothioyl)piperazin-1-yl)-1,4-dihydroquinoline-3-carboxylic acid (38)

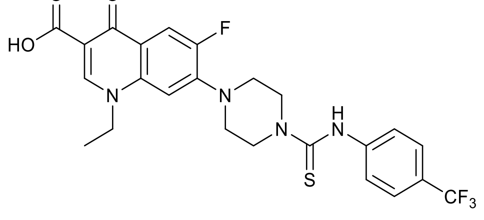

Prepared following *general procedure B* from the crude **38c** (150 mg, 0.47 mmol) and 4-(trifluoromethyl)phenyl isothiocyanate. Purification by flash silica column chromatography (1–2%

MeOH/DCM) gave **38** as a white solid (55 mg, 20%). R_f = 0.40 (SiO_2; DCM:MeOH, 90.10); **^{1}H NMR** (400 MHz, DMSO-d_6) δ 15.35 (s, 1H), 9.75 (s, 1H), 8.97 (s, 1H), 7.96 (d, *J* = 13.2 Hz, 1H), 7.67 (d, *J* = 8.5 Hz, 2H), 7.62 – 7.55 (m, 2H), 7.22 (d, *J* = 7.2 Hz, 1H), 4.61 (q, *J* = 7.1 Hz, 2H), 4.17 (t, *J* = 5.0 Hz, 4H), 3.50 (t, *J* = 5.0 Hz, 4H), 1.44 (t, *J* = 7.1 Hz, 3H); **HRMS** *m/z* (ESI^-) found 521.1284, $C_{24}H_{21}F_4N_4O_3S$ ($[M-H]^-$) requires 521.1276.

Ethyl 6,7-difluoro-1-isopropyl-4-oxo-1,4-dihydroquinoline-3-carboxylate (39a)

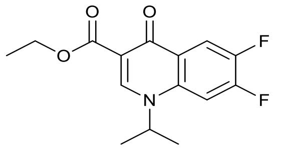

Prepared following *general procedure F from* **1a** (1.0 g, 3.3 mmol) and isopropylamine. Purification by flash silica column chromatography (25–100% EtOAc/petroleum ether) gave **39a** as an off-white solid (890 mg, 92%). R_f = 0.19 (SiO_2; Petroleum ether:EtOAc, 50:50); **^{1}H NMR** (400 MHz, $CDCl_3$) δ: 8.60 (s, 1H), 8.34–8.25 (m, 1H), 7.39 (dd, *J* = 11.9, 6.2 Hz, 1H), 4.72 (sept, *J* = 6.6 Hz, 1H), 4.39 (q, *J* = 7.1 Hz, 2H), 1.60 (d, *J* = 6.7 Hz, 6H), 1.39 (t, *J* = 7.1 Hz, 3H); **LRMS** *m/z* (ESI^+) 296 ($[M+H]^+$). These data are in agreement with the literature.[16]

Ethyl 6-fluoro-1-isopropyl-4-oxo-7-(piperazin-1-yl)-1,4-dihydroquinoline-3-carboxylate (39b)

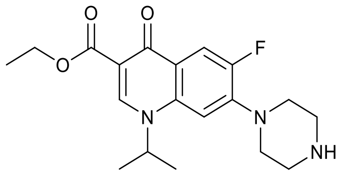

Prepared following *general procedure E* from **39a** (840 mg, 2.9 mmol) and piperazine. Purification by flash silica column chromatography (0–12% MeOH/DCM [+1% Et_3N]) gave **39b** as a pale-yellow oil (1.0 g, 99%). R_f = 0.17 (SiO_2; DCM:MeOH:Et_3N, 94:5:1); **^{1}H NMR** (400 MHz, $CDCl_3$) δ: 8.57 (s, 1H), 8.12 (d, *J* = 13.2 Hz, 1H), 6.87 (d, *J* = 6.8 Hz, 1H), 4.78 (sept, *J* = 6.6 Hz, 1H), 4.39 (q, *J* = 7.1 Hz, 2H), 3.24–3.17 (m, 4H), 3.11–3.05 (m, 4H), 1.60 (d, *J* = 6.6 Hz, 6H), 1.41 (t, *J* = 7.1 Hz, 3H); **LRMS** *m/z* (ESI^+) 362 ($[M+H]^+$).

6-Fluoro-1-isopropyl-4-oxo-7-(piperazin-1-yl)-1,4-dihydroquinoline-3-carboxylic acid (39c)

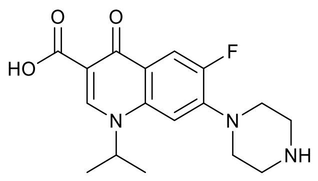

LiOH (470 mg, 11 mmol, 4.0 eq) was added to a solution of **39b** (1.0 g, 2.8 mmol, 1.0 eq) in 2:1 THF/H_2O (30 mL). The reaction was stirred at RT for 3 h, and the THF removed *in vacuo*. Precipitation at 0 °C followed by filtration gave **39c** as an off-white solid (680 mg, 72%). R_f = 0.02 (SiO_2; DCM:MeOH:Et_3N, 94:5:1); **LRMS** *m/z* (ESI^+) 334 ($[M+H]^+$); **HRMS** *m/z* (ESI^+) found 334.1559, $C_{17}H_{21}FN_3O_3$ ($[M+H]^+$) requires 334.1562.

6-Fluoro-1-isopropyl-4-oxo-7-(4-((4-(trifluoromethyl)phenyl)carbamothioyl)piperazin-1-yl)-1,4-dihydroquinoline-3-carboxylic acid (39)

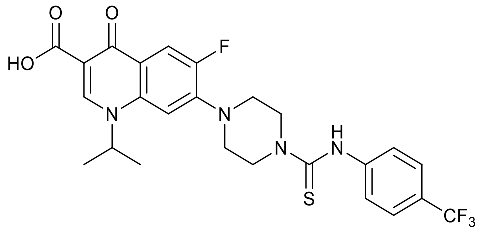

Prepared following *general procedure B* from **39c** (300 mg, 0.88 mmol) and 4-(trifluoromethyl)phenyl isothiocyanate. Purification by flash silica column chromatography (0–10% MeOH/DCM [+0.5% formic acid]) gave **39** as a pale-yellow solid (220 mg, 48%). R_f = 0.26 (SiO_2; DCM:MeOH:formic acid, 94.5:5:0.5); **^{1}H NMR** (600 MHz, DMSO-d_6) δ: 9.72 (s, 1H), 8.79 (s, 1H), 7.97 (d, *J* = 13.1 Hz, 1H), 7.66 (d, *J* = 8.6 Hz, 2H), 7.58 (d, *J* = 8.6 Hz, 2H), 7.37 (d, *J* = 7.1 Hz, 1H), 5.31 (sept, *J* = 6.6 Hz, 1H), 4.21–4.11 (t, *J* = 4.6 Hz, 4H), 3.55–3.44 (t, *J* = 4.6 Hz, 4H), 1.57 (d, *J* = 6.6 Hz, 6H); **^{13}C NMR** (151 MHz, DMSO-d_6) δ: 181.2, 175.8 (d, *J* = 2.6 Hz), 166.1, 152.6 (d, *J* = 294 Hz), 144.9 (d, *J* = 10.5 Hz), 144.8, 143.7, 137.7, 125.2 (q, *J* = 3.8 Hz), 124.4 (q, *J* = 272 Hz), 124.2, 123.8 (q, *J* = 31.9 Hz), 119.4 (d, *J* = 7.6 Hz), 111.2 (d, *J* = 22.8 Hz), 107.1, 105.7 (d, *J* = 3.3 Hz), 52.6, 48.9 (d, *J* = 4.5 Hz), 47.8, 21.4; **LRMS** *m/z* (ESI^+) 537 ($[M+H]^+$); **HRMS** *m/z* (ESI^+) found 537.1589, $C_{25}H_{25}F_4N_4O_3S$ ($[M+H]^+$) requires 537.1578; **HPLC** Retention time 12.1 min (97.9%, 280 nm).

6-Fluoro-1-isopropyl-4-oxo-7-(4-((4-(trifluoromethyl)phenyl)carbamoyl)piperazin-1-yl)-1,4-dihydroquinoline-3-carboxylic acid (40)

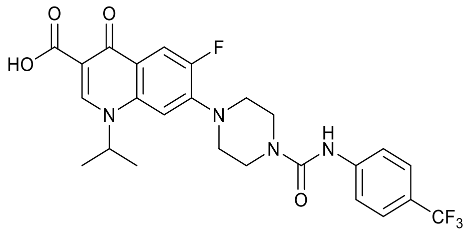

4-(Trifluoromethyl)phenyl isocyanate (110 mg, 0.59 mmol) was added to a suspension of **39c** (250 mg, 0.36 mmol) and Cs_2CO_3 (150 mg, 0.45 mmol) in 1:1 anhydrous MeCN/DCM. The reaction was stirred at 60 °C overnight. NH_4Cl (20 mL) was added, the product extracted with DCM (3 × 30 mL), washed with water (30 mL), dried, and the solvent removed *in vacuo*. Purification by flash silica column chromatography (0–10% MeOH/DCM [+0.5% formic acid]) gave **40** as a white solid (238 mg, 62%). R_f = 0.21 (SiO_2; DCM:MeOH:formic acid, 94.5:5:0.5); **^{1}H NMR** (600 MHz, DMSO-d_6) δ: 9.05 (s, 1H), 8.78 (s, 1H), 7.96 (d, *J* = 13.0 Hz, 1H), 7.72 (d, *J* = 8.6 Hz, 2H), 7.60 (d, *J* = 8.6 Hz, 2H), 7.38 (d, *J* = 7.1 Hz, 1H), 5.30 (sept, *J* = 6.5 Hz, 1H), 3.73–3.69 (t, *J* = 4.8 Hz, 4H), 3.40–3.36 (t, *J* = 4.8 Hz, 4H), 1.57 (d, *J* = 6.5 Hz, 6H); **^{13}C NMR** (151 MHz, DMSO-*d_6*) δ: 175.8 (d, *J* = 2.6 Hz), 166.1, 154.5, 152.9 (d, *J* = 249 Hz), 145.3 (d, *J* = 10.5 Hz), 144.3, 143.7, 137.7, 125.6 (q, *J* = 3.8 Hz), 124.6 (q, *J* = 271 Hz), 121.7 (q, *J* = 32.0 Hz), 119.6 (d, *J* = 7.7 Hz), 118.9, 111.2 (d, *J* = 23.0 Hz), 107.1, 106.1 (d, *J* = 3.1 Hz), 52.7, 49.4 (d, *J* = 4.5 Hz), 43.6, 21.4; **LRMS** *m/z* (ESI^+) 521 ($[M+H]^+$); **HRMS** *m/z* (ESI^+) found 521.1796, $C_{25}H_{25}F_4N_4O_4$ ($[M+H]^+$) requires 521.1806; **HPLC** Retention time 11.7 min (99.8%, 280 nm).

NMR Spectra of Final Compounds

7-(4-(Cyclohexylcarbamothioyl)piperazin-1-yl)-1-cyclopropyl-6-fluoro-4-oxo-1,4-dihydroquinoline-3-carboxylic acid (4)

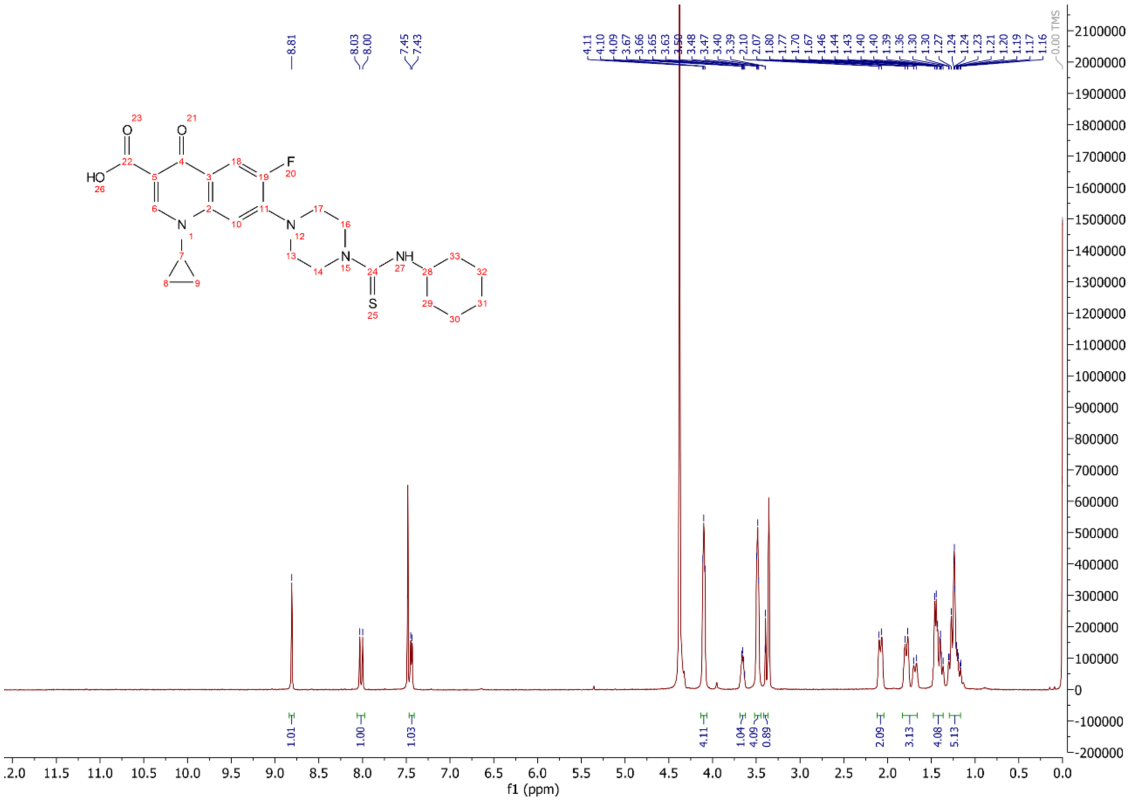

^1H NMR (400 MHz, $CDCl_3$: CD_3OD [2:1]) at 298 K of **4**.

1-Cyclopropyl-6-fluoro-7-(4-((2-morpholinoethyl)carbamothioyl)piperazin-1-yl)-4-oxo-1,4-dihydroquinoline-3-carboxylic acid (5)

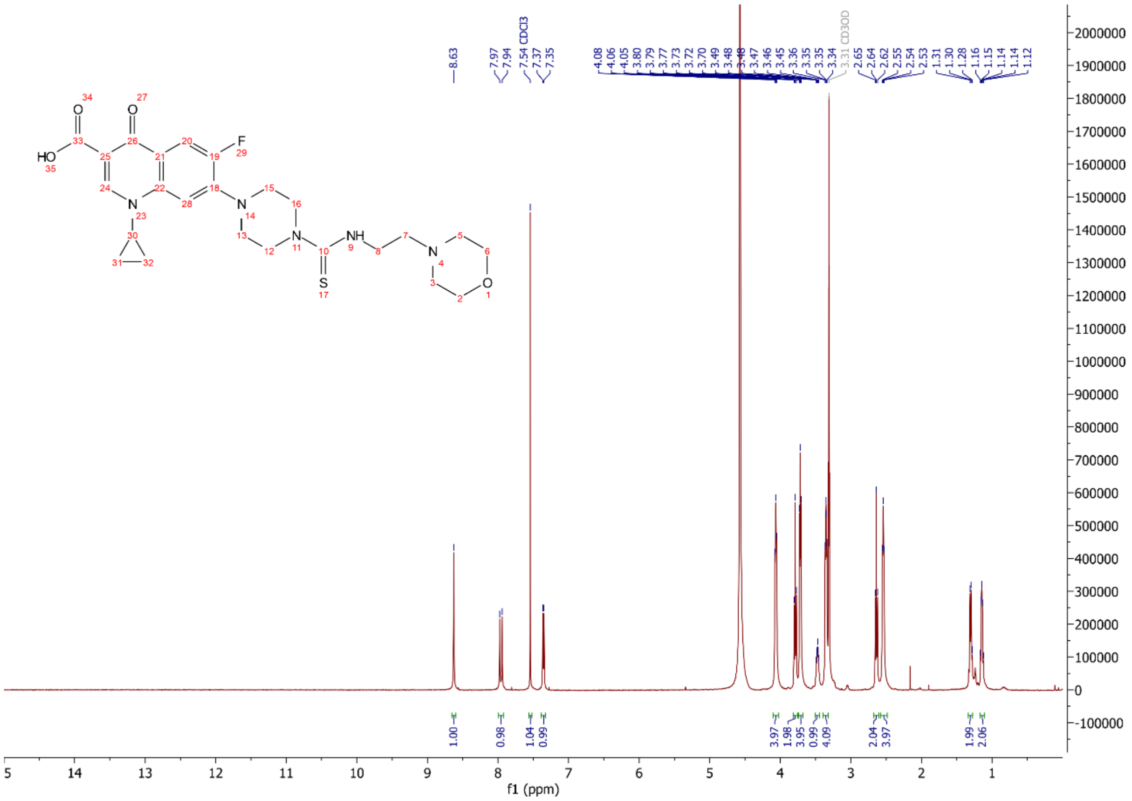

^{1}H NMR (400 MHz, $CDCl_3$: CD_3OD [2:1]) at 298 K of **5**.

7-(4-((4-Cyanophenyl)carbamothioyl)piperazin-1-yl)-1-cyclopropyl-6-fluoro-4-oxo-1,4-dihydroquinoline-3-carboxylic acid (6)

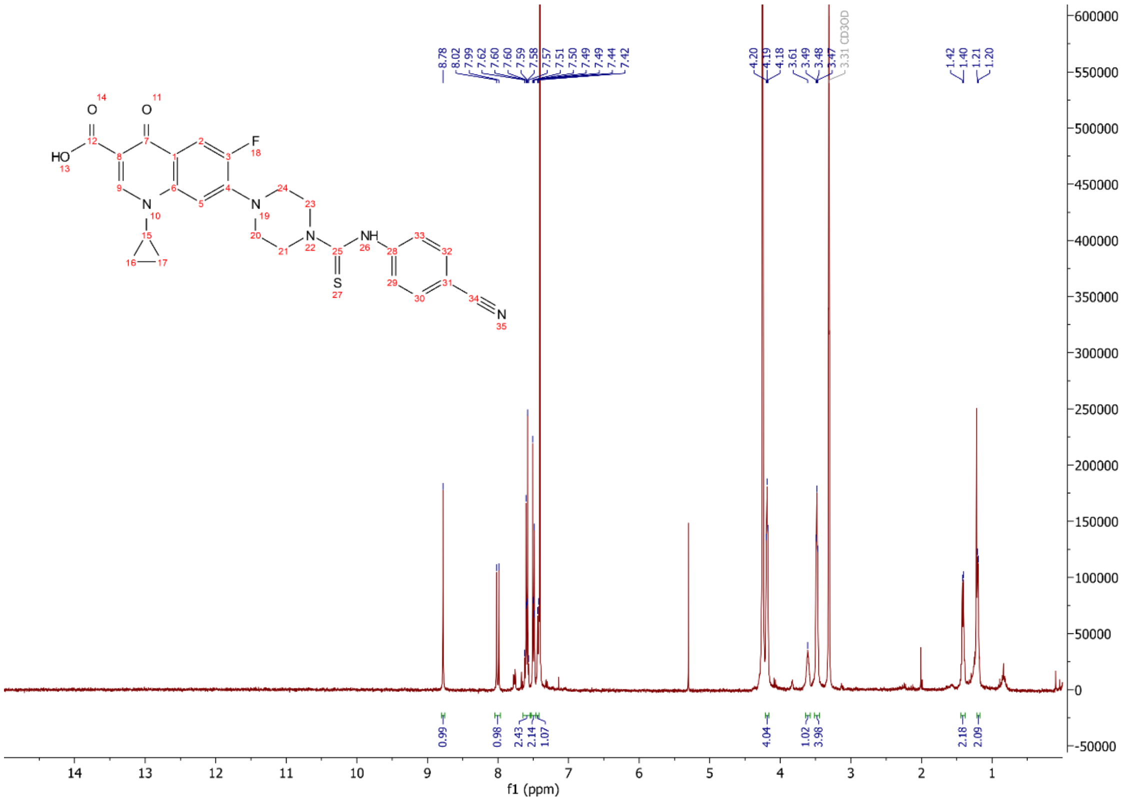

^1H NMR (400 MHz, $CDCl_3$: CD_3OD [3:1]) at 298 K of **6**.

1-Cyclopropyl-6-fluoro-4-oxo-7-(4-(pyridin-4-ylcarbamothioyl)piperazin- 1-yl)-1,4-dihydroquinoline-3-carboxylic acid (7)

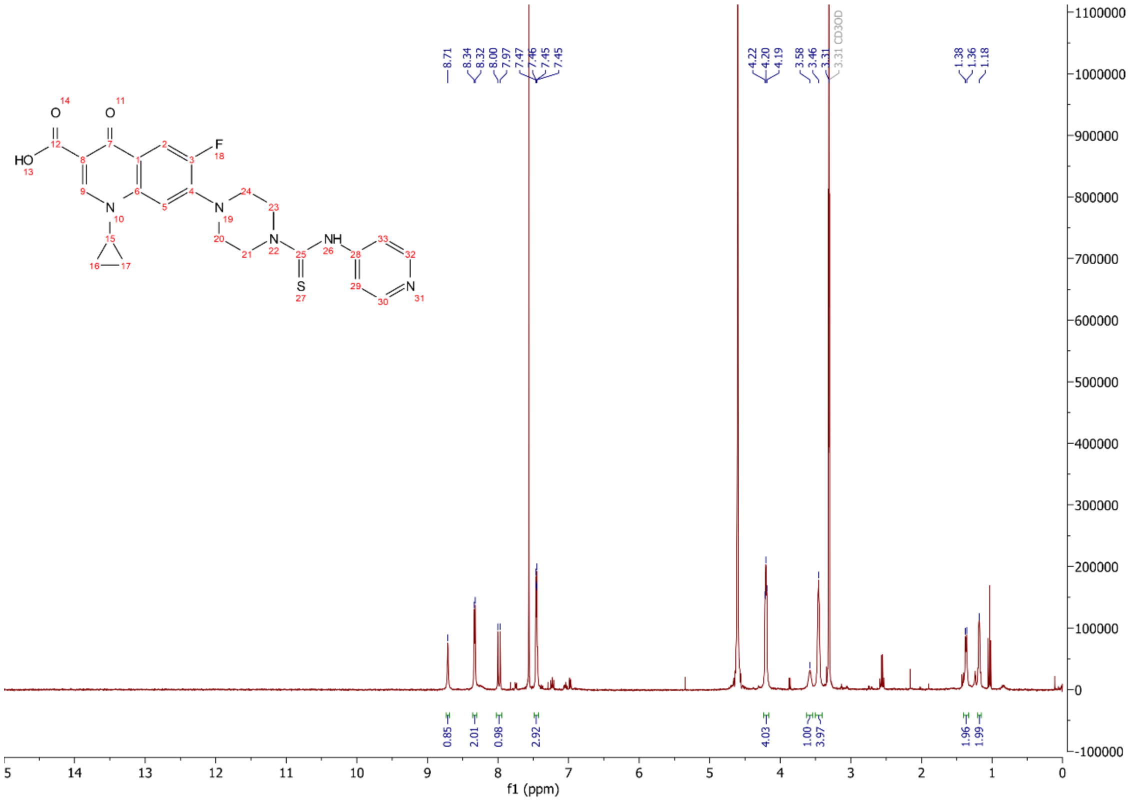

^{1}H NMR (400 MHz, $CDCl_3$: CD_3OD [3:1]) at 298 K of **7**.

7-(4-(1*H* -Imidazole-1-carbonothioyl)piperazin-1-yl)-1-cyclopropyl-6- fluoro-4-oxo-1,4-dihydroquinoline-3-carboxylic acid (8)

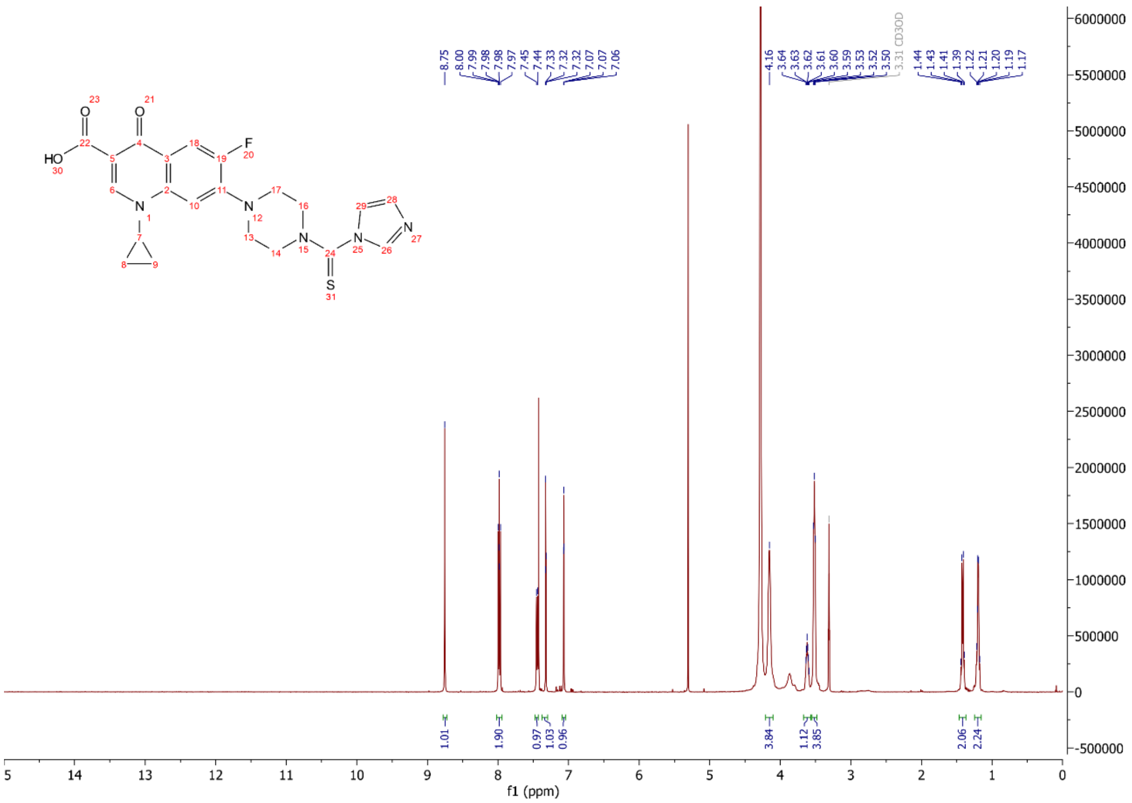

^{1}H NMR (400 MHz, $CDCl_3$: CD_3OD [3:1]) at 298 K of **8**.

1-Cyclopropyl-6-fluoro-4-oxo-7-(4-(phenoxycarbonothioyl)piperazin-1-yl)-1,4-dihydroquinoline-3-carboxylic acid (9)

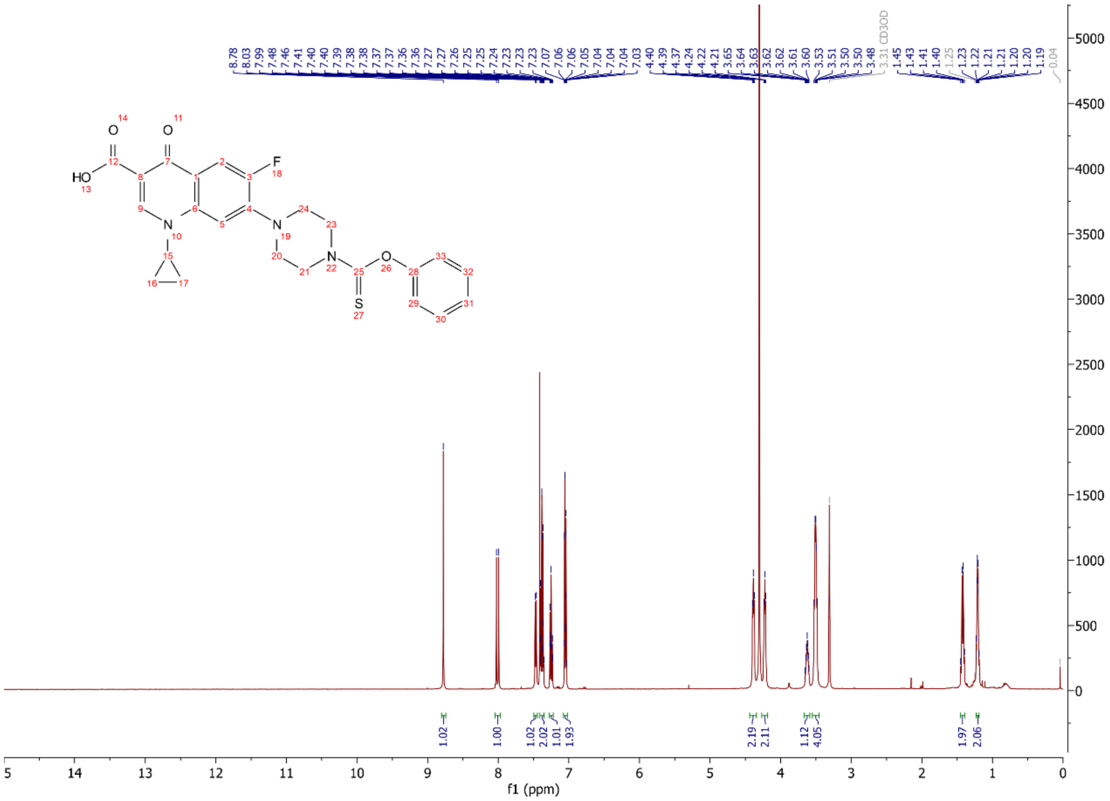

^{1}H NMR (400 MHz, $CDCl_3$: CD_3OD [4:1]) at 298 K of **9**.

1-Cyclopropyl-6-fluoro-4-oxo-7-(4-(2-oxo-1,2-dihydropyridine-1-carbonothioyl) piperazin-1-yl)-1,4-dihydroquinoline-3-carboxylic acid (10)

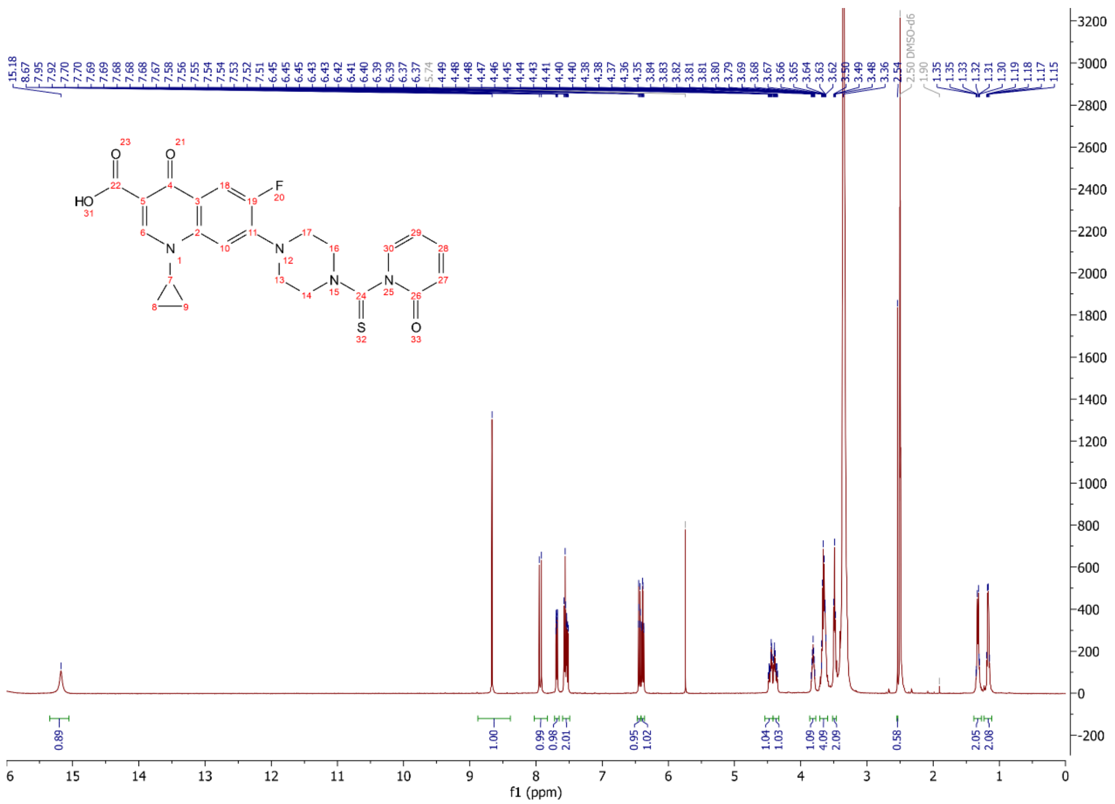

^{1}H NMR (400 MHz, DMSO-d_6) at 298 K of **10**.

1-Cyclopropyl-6-fluoro-4-oxo-7-(4-((4-(trifluoromethyl)phenyl)carbamoyl)piperazin-1-yl)-1,4-dihydroquinoline-3-carboxylic acid (11)

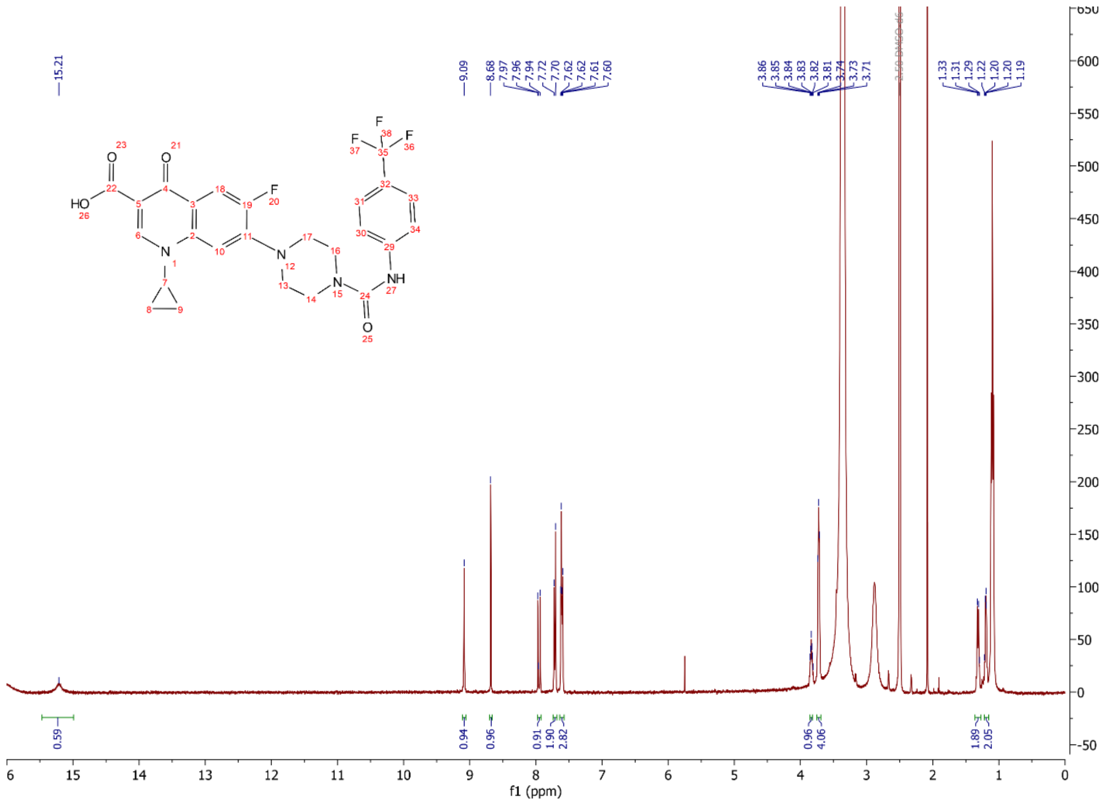

^{1}H NMR (400 MHz, DMSO-d_6) at 298 K of **11**.

1-Cyclopropyl-6-fluoro-4-oxo-7-(4-(4-(trifluoromethyl)benzoyl)piperazin-1-yl)-1,4-dihydroquinoline-3-carboxylic acid (12)

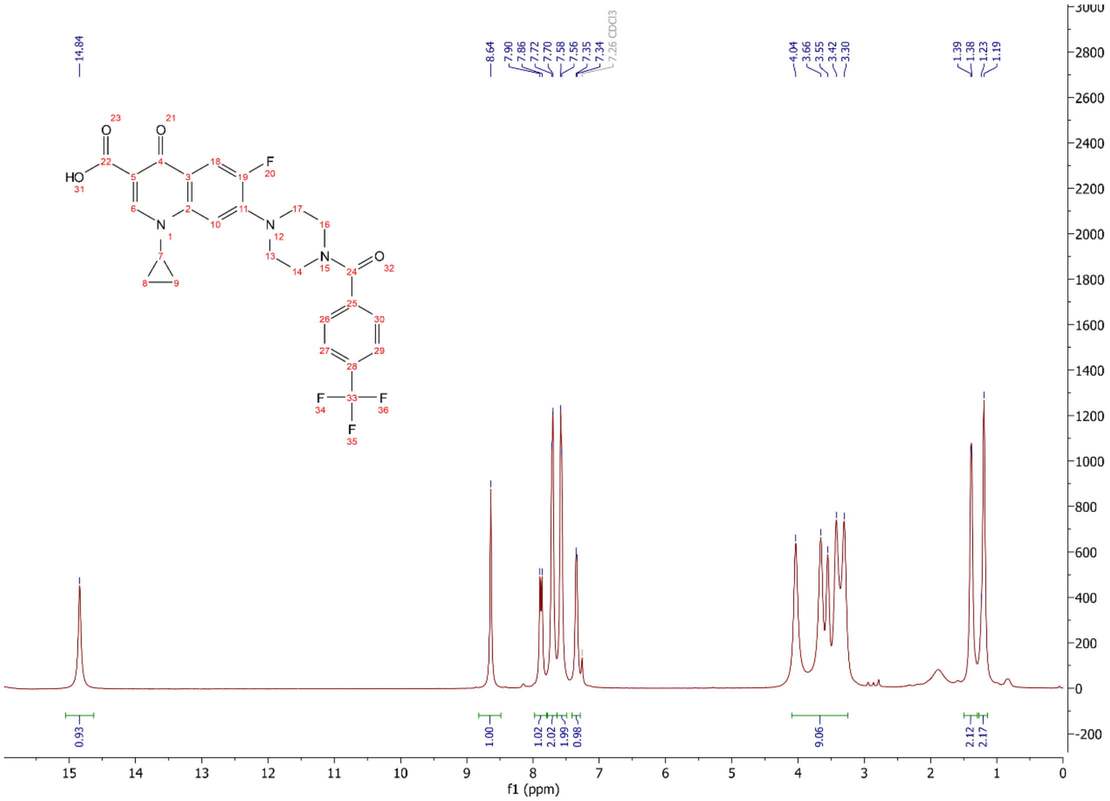

^{1}H NMR (400 MHz, $CDCl_3$) at 298 K of **12**.

1-Cyclopropyl-6-fluoro-4-oxo-7-(4-((4-(trifluoromethyl)phenyl)sulfonyl)piperazin-1-yl)-1,4-dihydroquinoline-3-carboxylic acid (13)

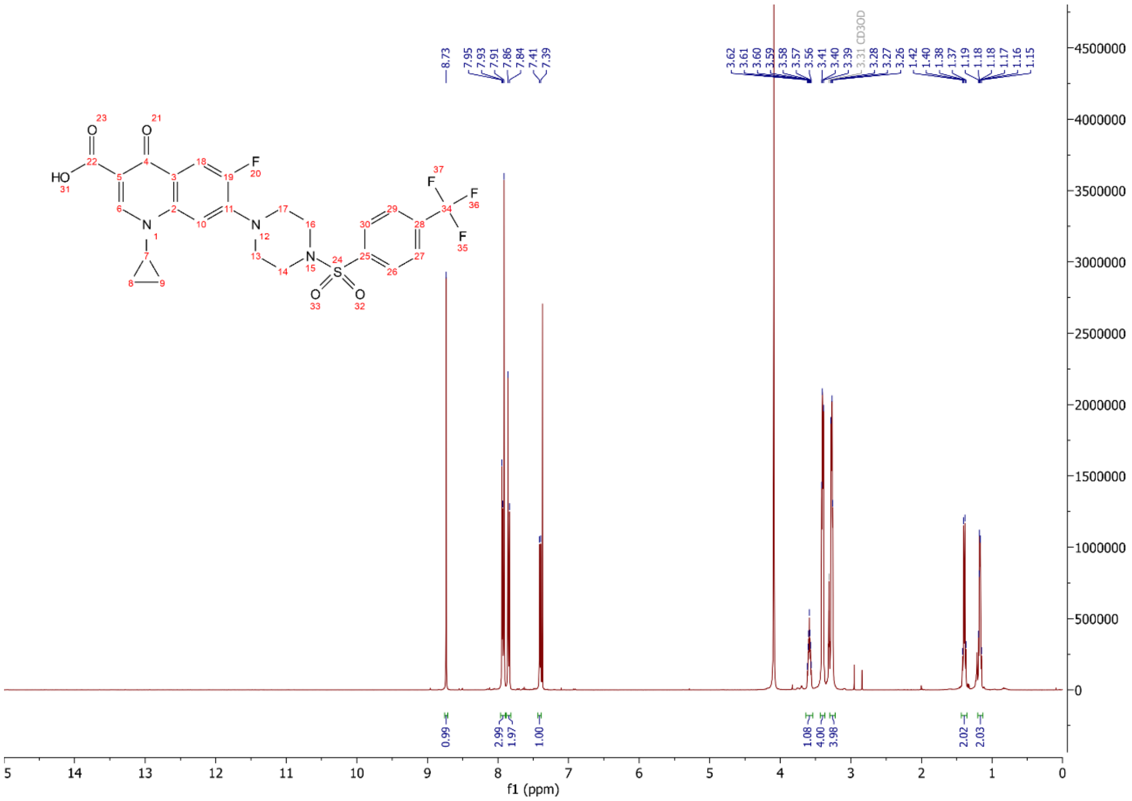

^{1}H NMR (400 MHz, $CDCl_3$: CD_3OD [4:1]) at 298 K of **13**.

1-Cyclopropyl-6-fluoro-4-oxo-7-(4-(6-(trifluoromethyl)benzo[*d*]thiazol-2 -yl)piperazin-1-yl)-1,4-dihydroquinoline-3-carboxylic acid (14)

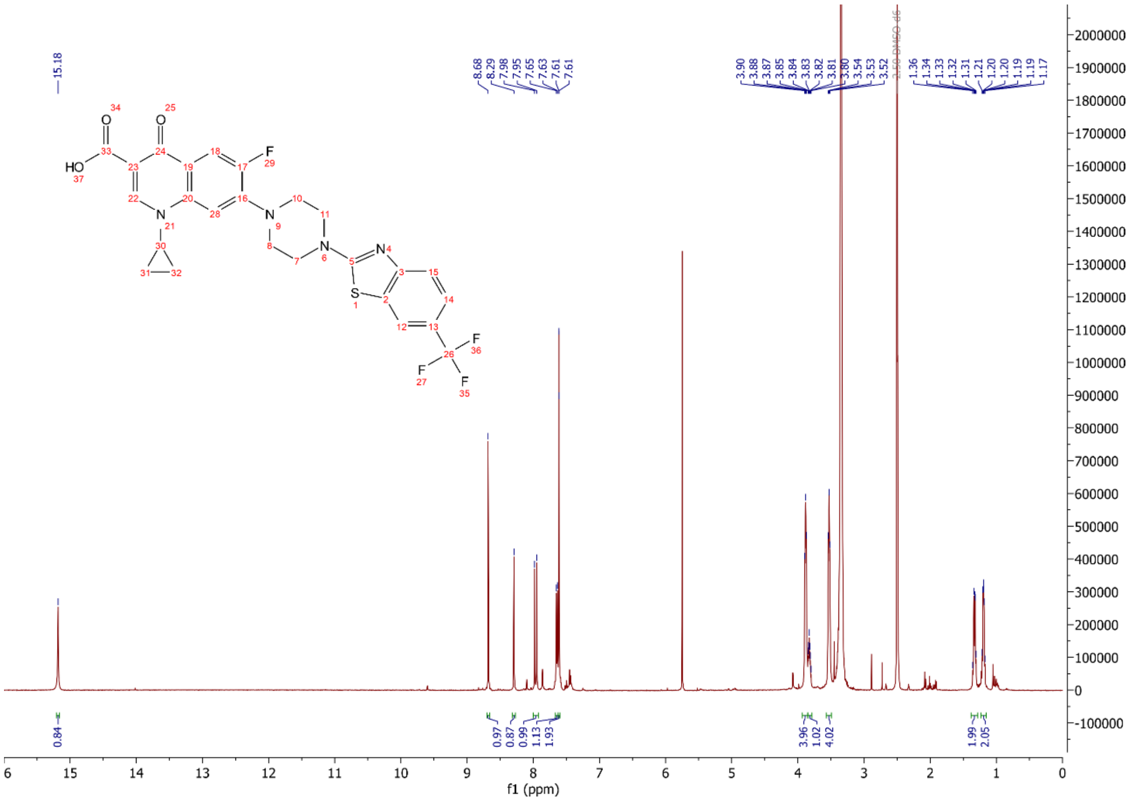

^{1}H NMR (400 MHz, DMSO-d_6) at 298 K of **14**.

7-(4-((Benzylthio)((4-(trifluoromethyl)phenyl)imino)methyl)piperazin-1-yl)-1-cyclopropyl-6-fluoro-4-oxo-1,4-dihydroquinoline-3-carboxylic acid (15)

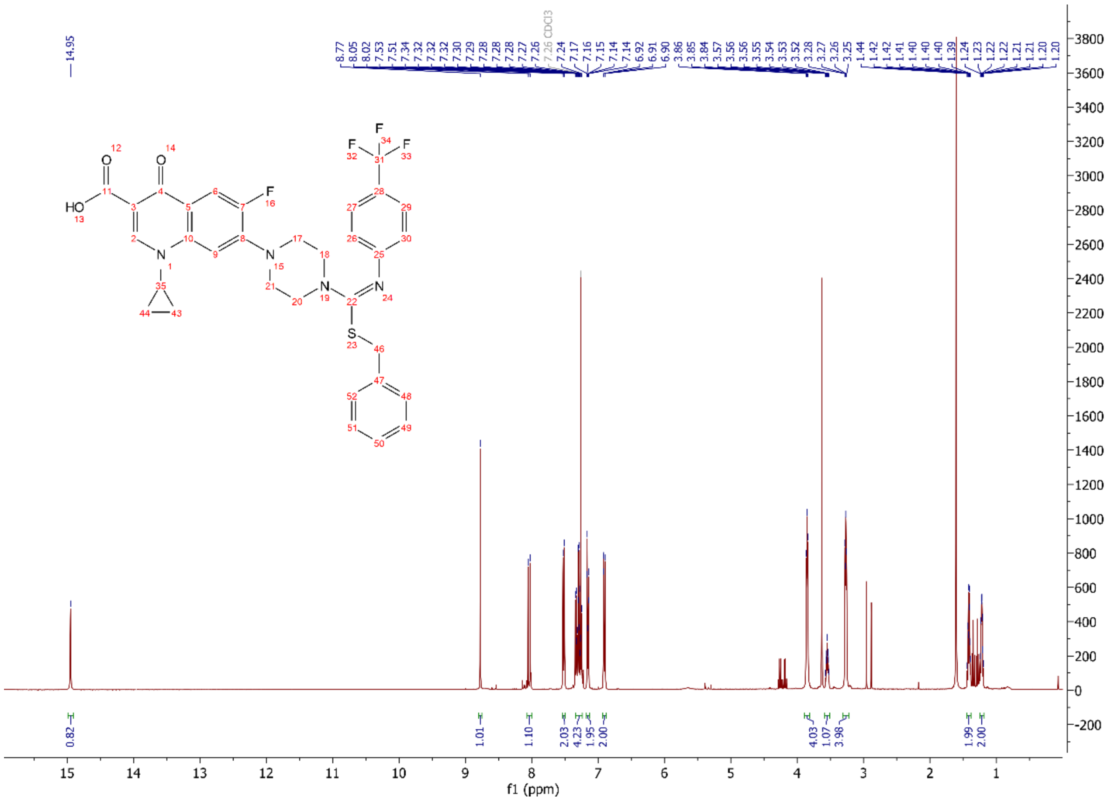

^{1}H NMR (400 MHz, $CDCl_3$) at 298 K of **15**.

1-Cyclopropyl-6-fluoro-7-(4-(((2-methoxy-2-oxoethyl)thio)((4-(trifluoromethyl)phenyl)imino)methyl)piperazin-1-yl)-4-oxo-1,4-dihydroquinoline-3-carboxylic acid (16)

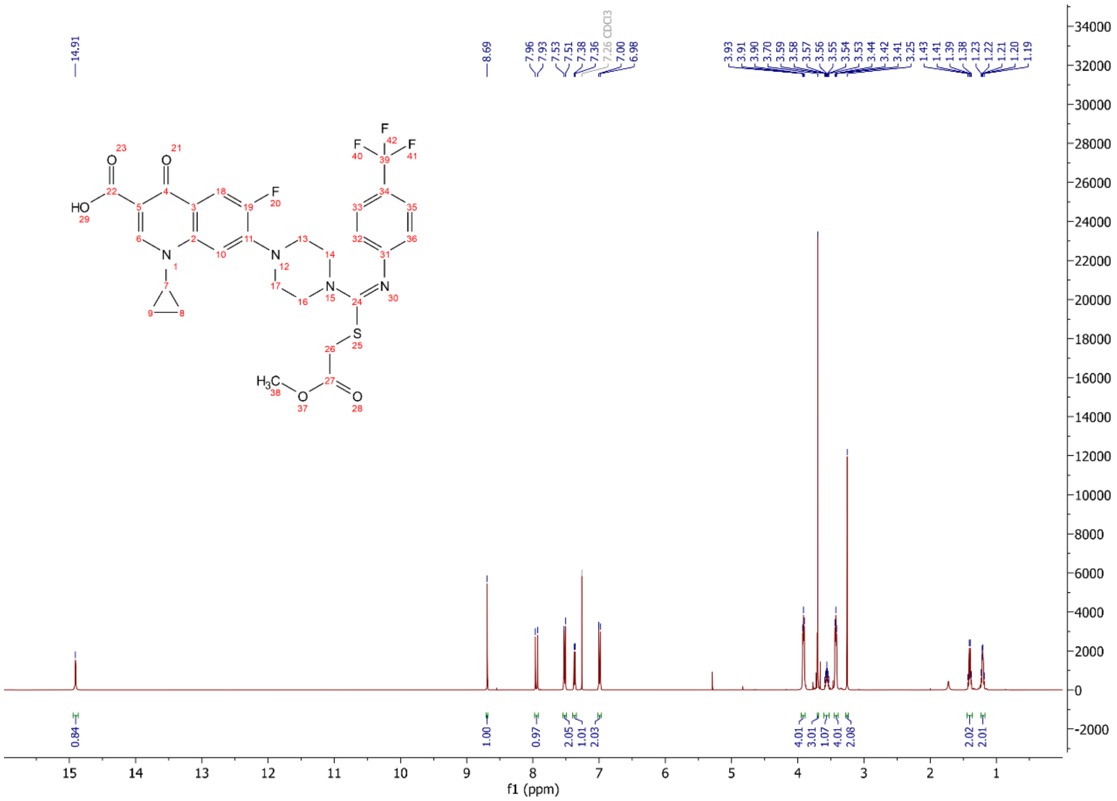

^{1}H NMR (400 MHz, $CDCl_3$) at 298 K of **16**.

7-(4-(((2-Amino-2-oxoethyl)thio)((4-(trifluoromethyl)phenyl)imino)methyl)piperazin-1-yl)-1-cyclopropyl-6-fluoro-4-oxo-1,4-dihydroquinoline-3-carboxylic acid (17)

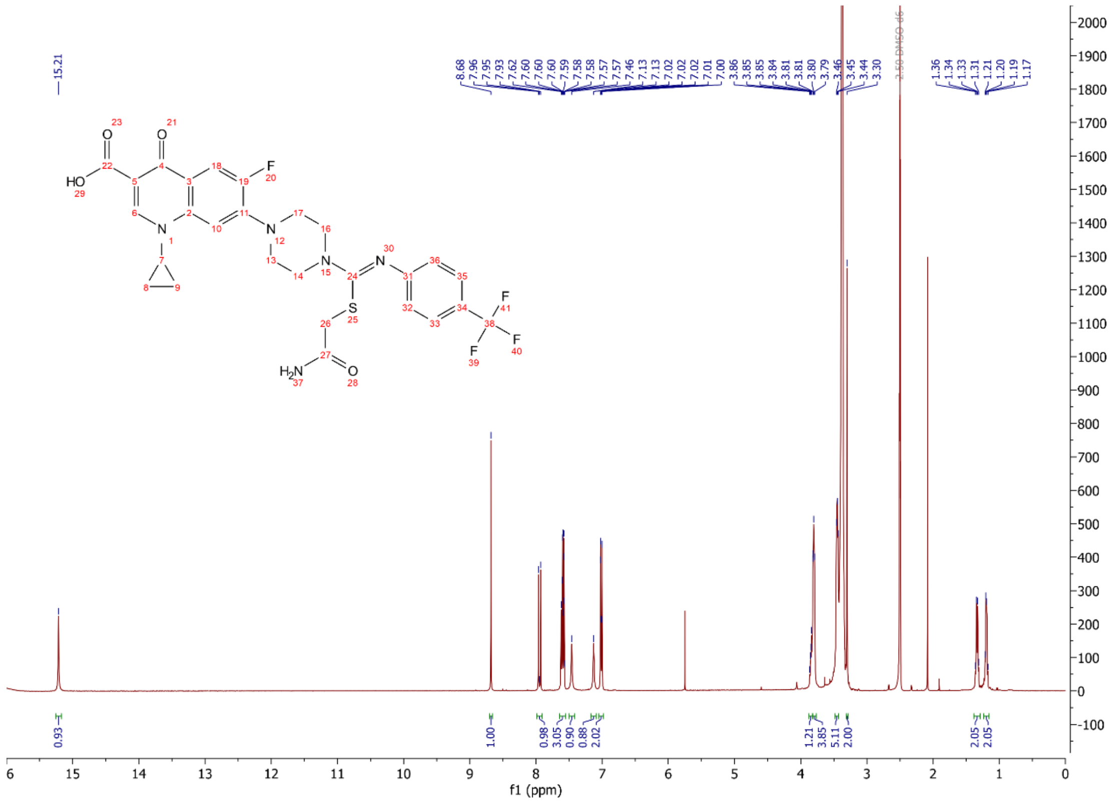

^{1}H NMR (400 MHz, DMSO-d_6) at 298 K of **17**.

1-Cyclopropyl-6-fluoro-4-oxo-7-(4-(3-(4-(trifluoromethyl)phenyl)thioureido)piperidin-1-yl)-1,4-dihydroquinoline-3-carboxylic acid (18)

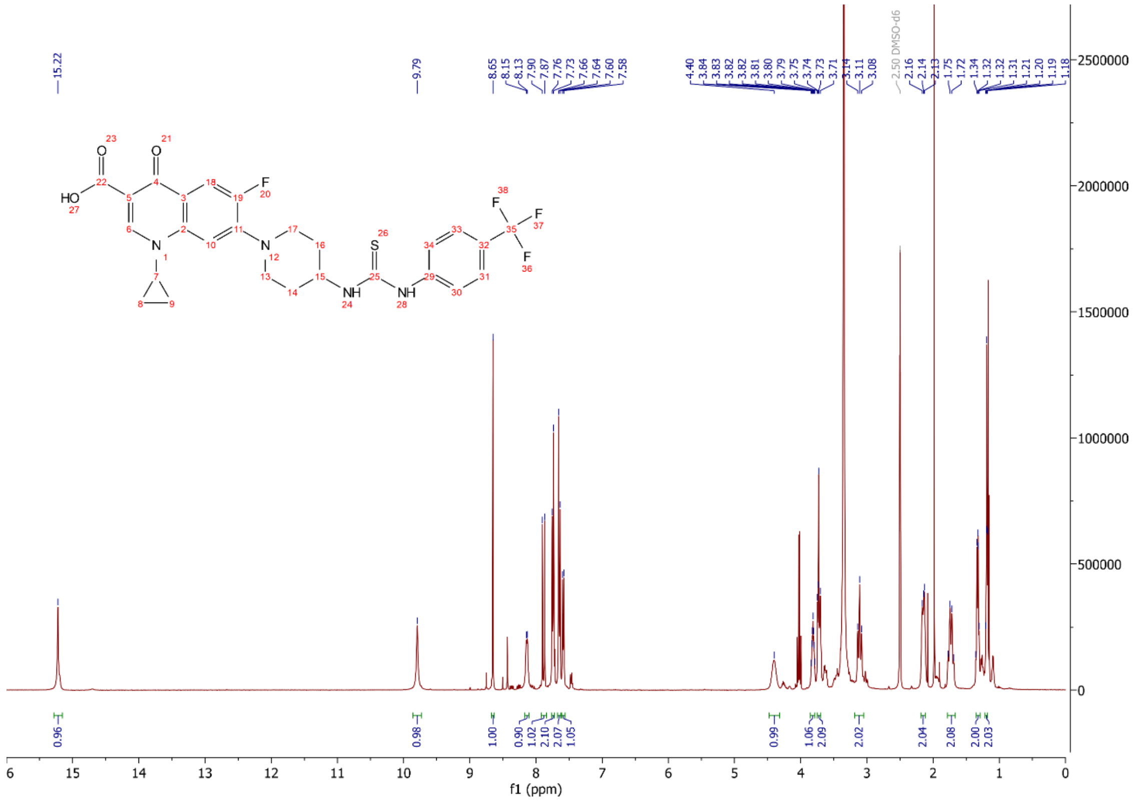

^{1}H NMR (400 MHz, DMSO-d_6) at 298 K of **18**.

4-(2-Fluorophenyl)-*N*-(4-(trifluoromethyl)phenyl)piperazine-1-carbothioamide (19)

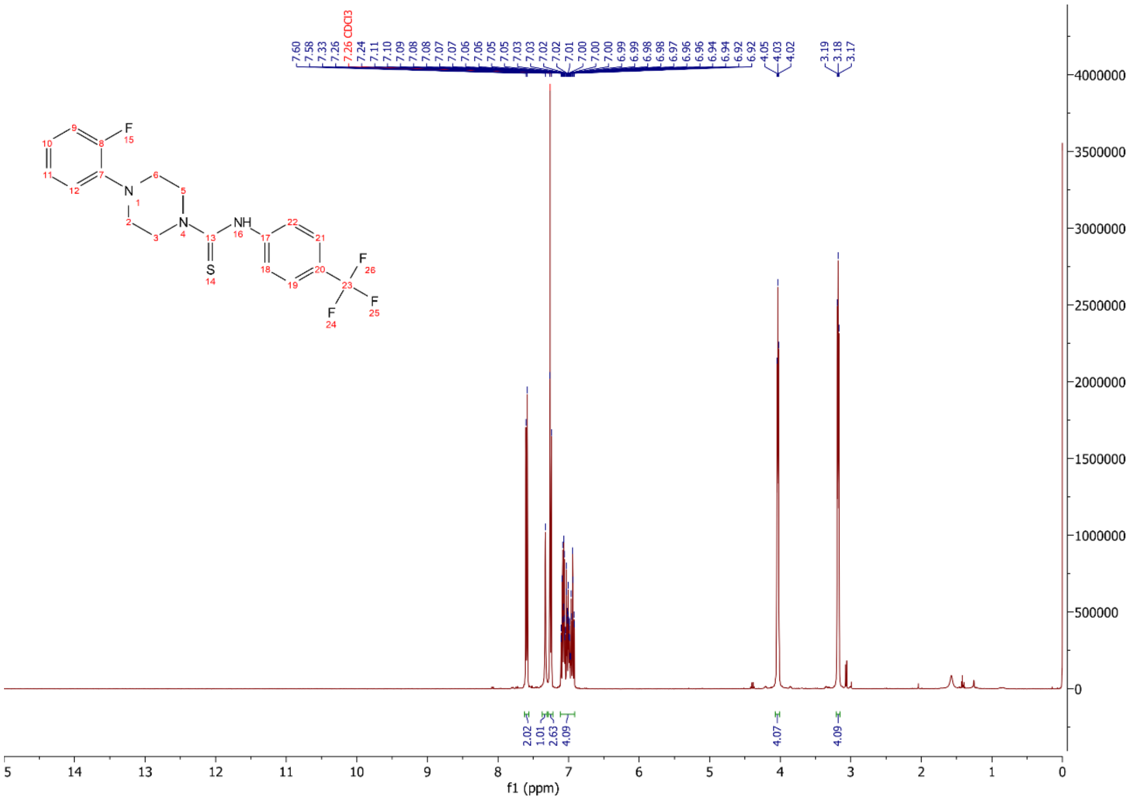

^{1}H NMR (400 MHz, $CDCl_3$) at 298 K of **19**.

Ethyl 4-(4-((4-(trifluoromethyl)phenyl)carbamothioyl)piperazin-1-yl)benzoate (20)

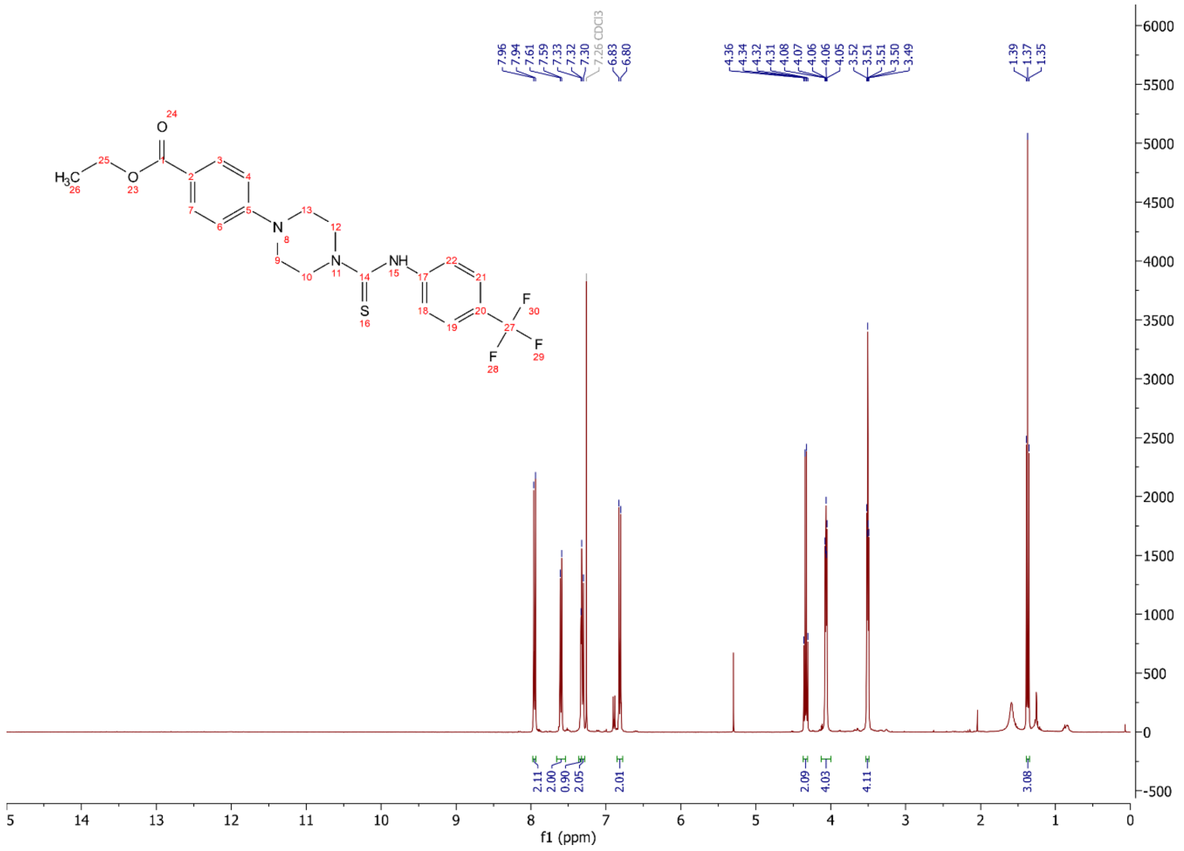

^{1}H NMR (400 MHz, $CDCl_3$) at 298 K of **20**.

4-(4-Acetyl-2-fluorophenyl)-*N*-(4-(trifluoromethyl)phenyl)piperazine-1-carbothioamide (21)

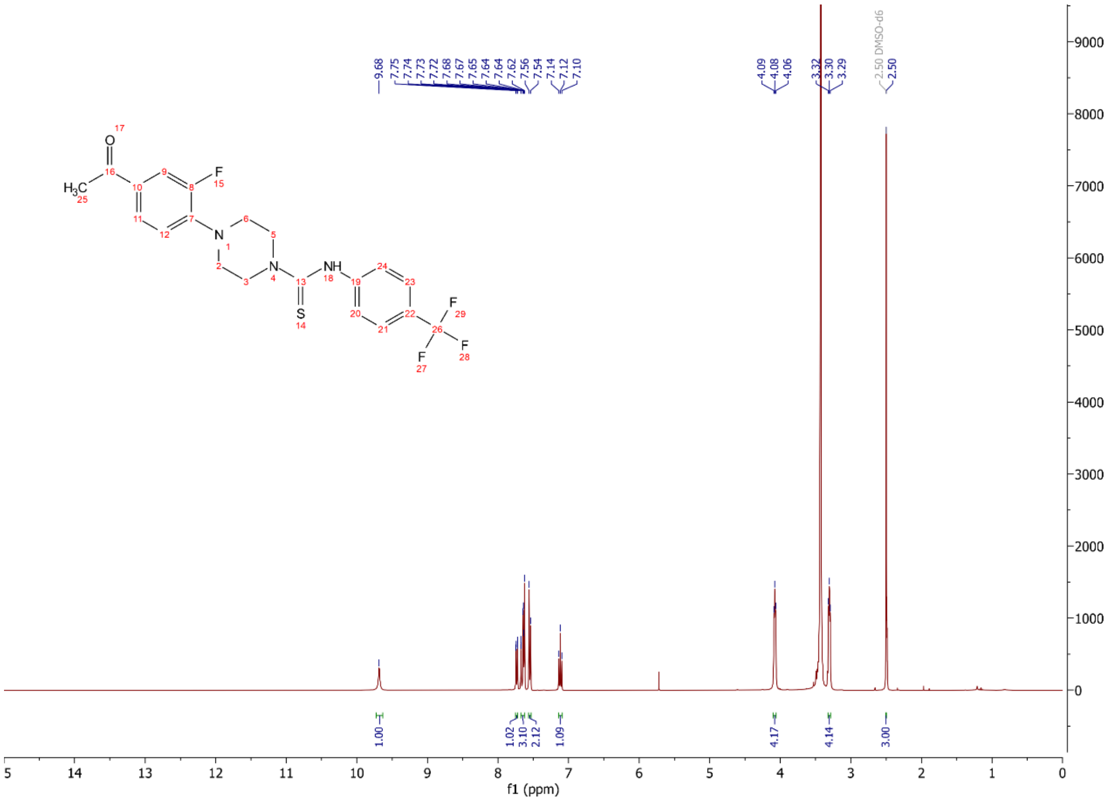

^{1}H NMR (400 MHz, DMSO-d_6) at 298 K of **21**.

4-(4-Cyano-2-fluorophenyl)-*N* -(4-(trifluoromethyl)phenyl)piperazine-1-carbothioamide (22)

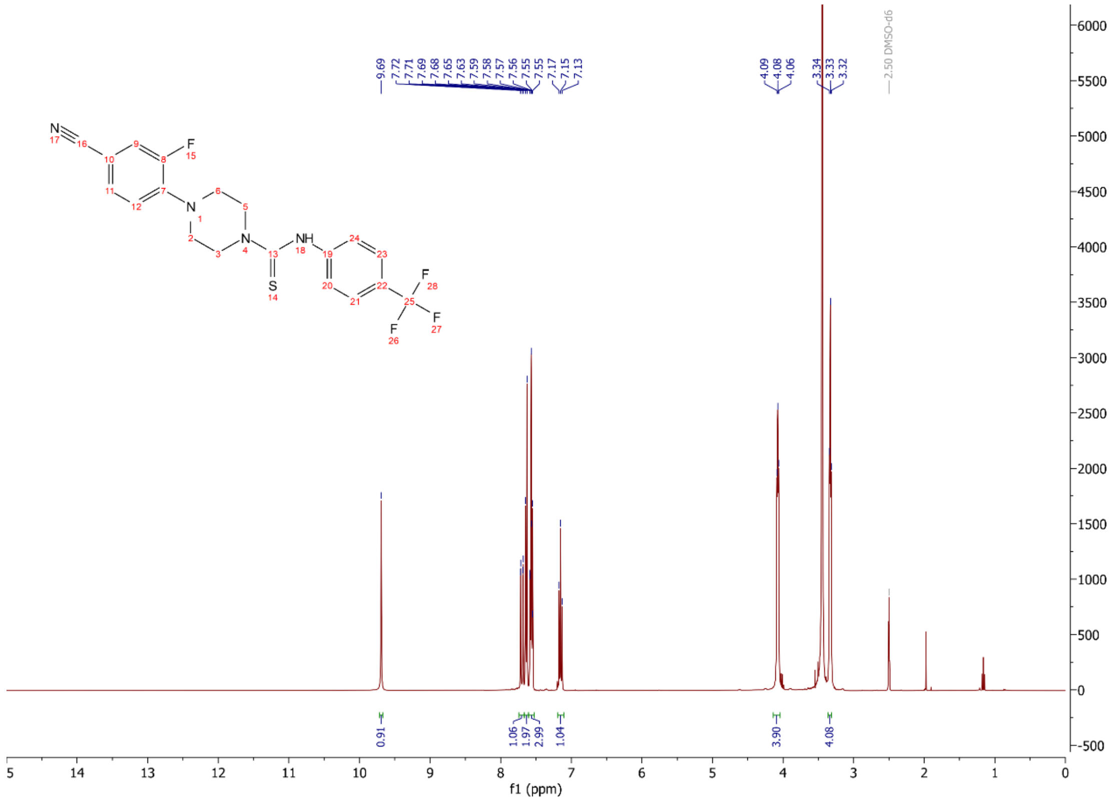

^{1}H NMR (400 MHz, DMSO-d_6) at 298 K of **22**.

3-Fluoro-4-(4-((4-(trifluoromethyl)phenyl)carbamothioyl)piperazin-1-yl) benzamide (23)

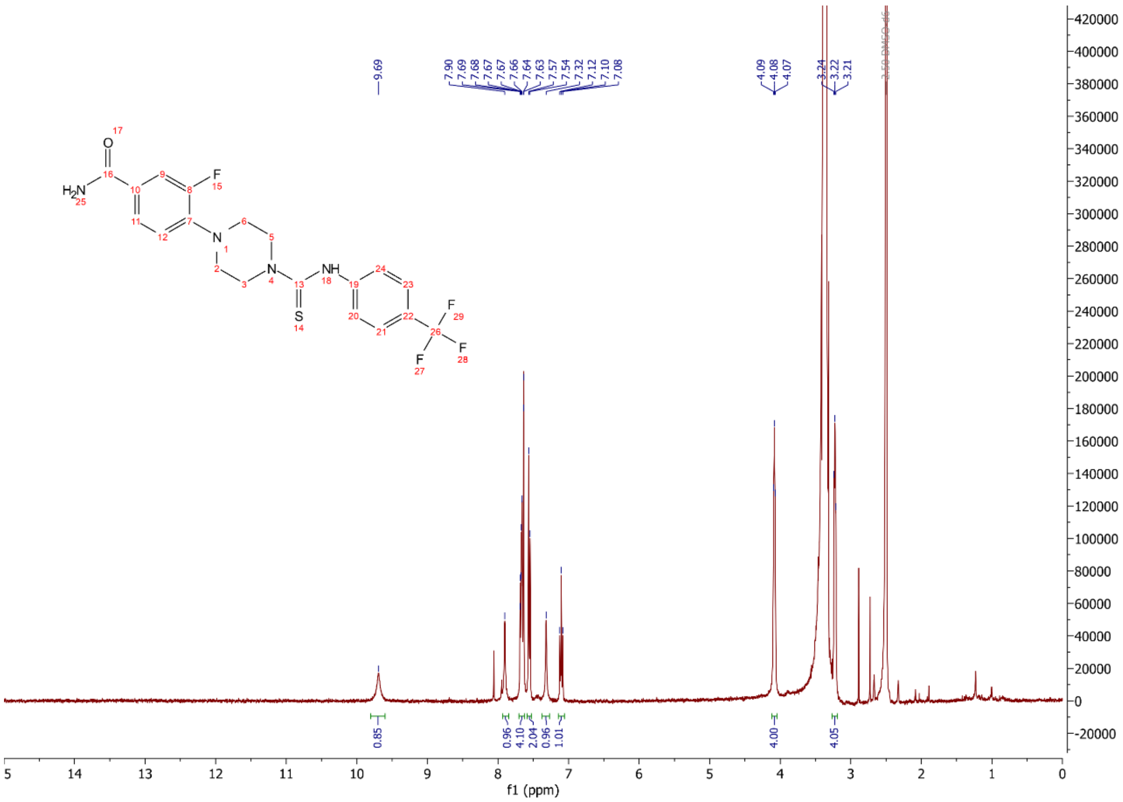

^{1}H NMR (400 MHz, DMSO-d_6) at 298 K of **23**.

Methyl 2-amino-5-fluoro-4-(4-((4-(trifluoromethyl)phenyl)carbamothioyl)piperazin-1-yl)benzoate (24)

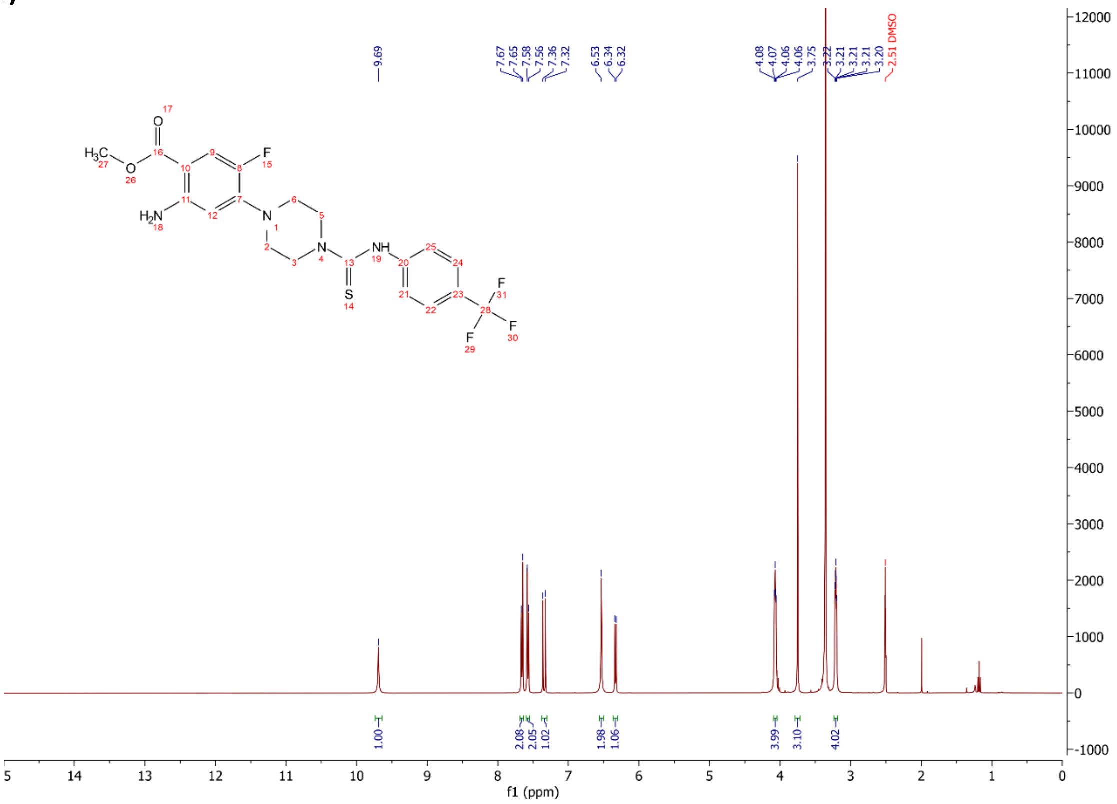

^{1}H NMR (400 MHz, DMSO-d_6) at 298 K of **24**.

4-(4-Benzoyl-2-fluorophenyl)-*N* -(4-(trifluoromethyl)phenyl)piperazine-1-carbothioamide (25)

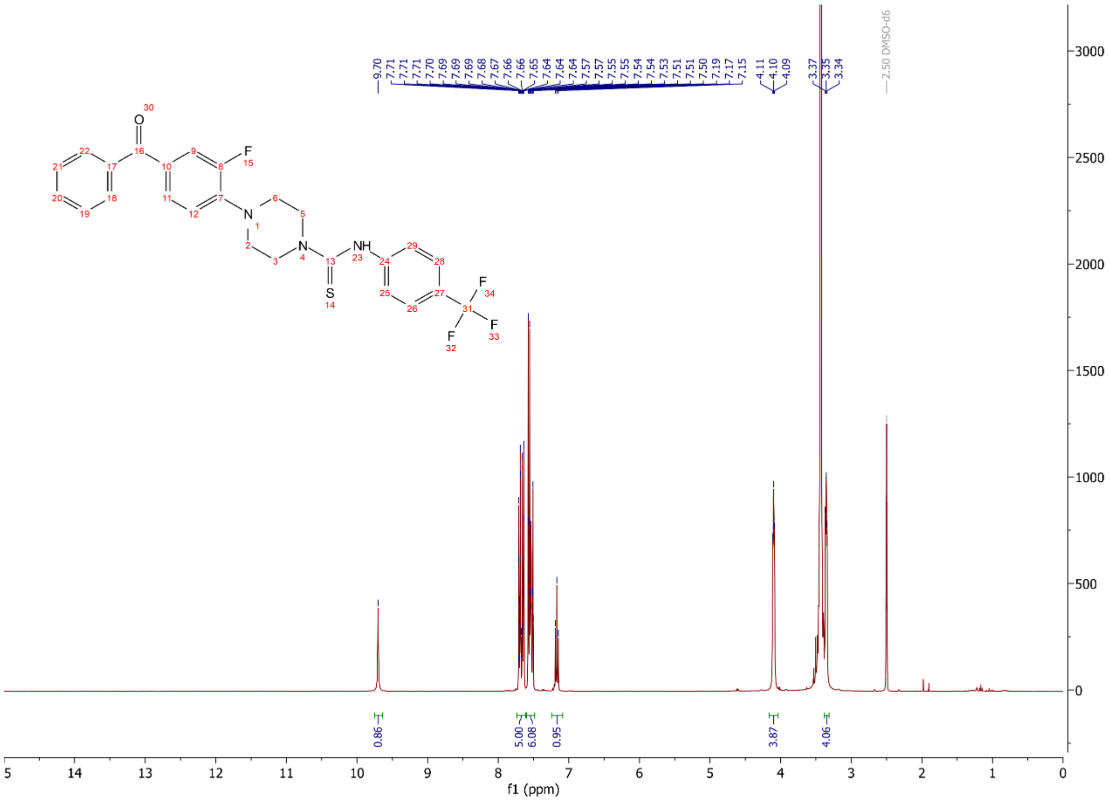

^{1}H NMR (400 MHz, DMSO-d_6) at 298 K of **25**.

4-(6-Fluoro-4-oxo-1,4-dihydroquinolin-7-yl)-N-(4-(trifluoromethyl)phenyl)piperazine-1-carbothioamide (26)

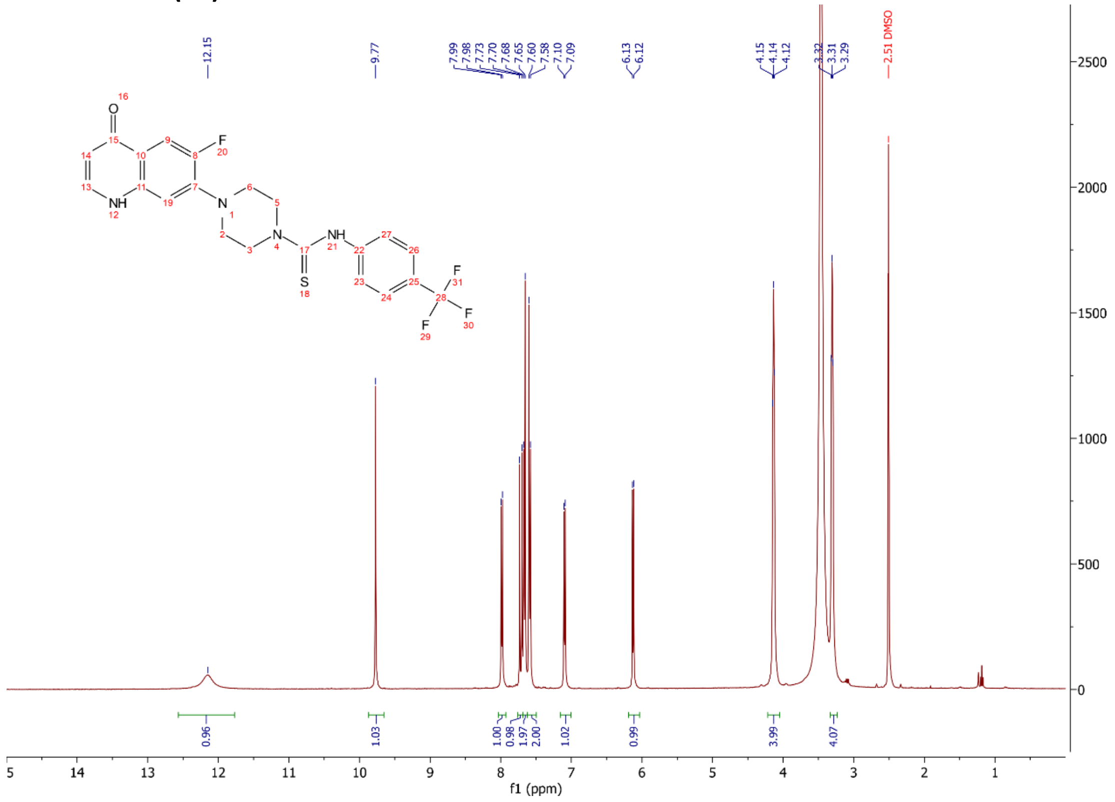

^{1}H NMR (400 MHz, DMSO-d_6) at 298 K of **26**.

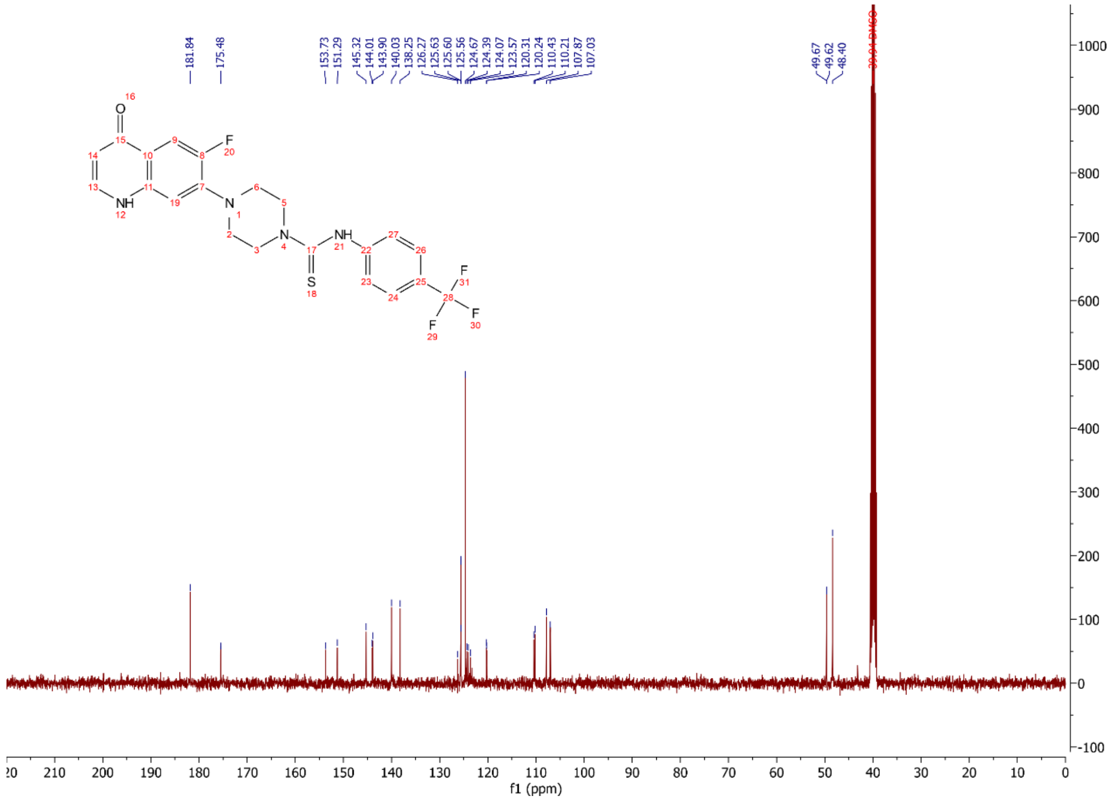

^{13}C NMR (101 MHz, DMSO-d_6) at 298 K of **26**.

4-(6-Fluoro-4-oxo-1,4-dihydroquinazolin-7-yl)-N-(4-(trifluoromethyl)phenyl)piperazine-1-carbothioamide (27)

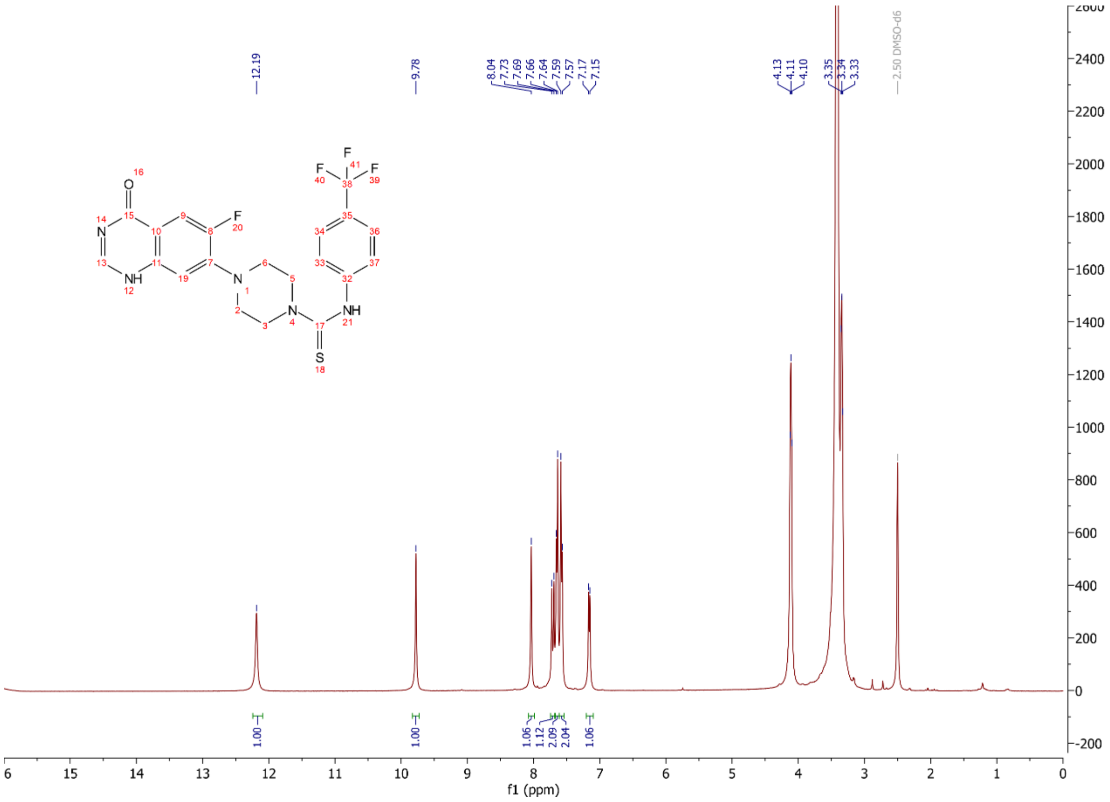

^{1}H NMR (400 MHz, DMSO-d_6) at 298 K of **27**.

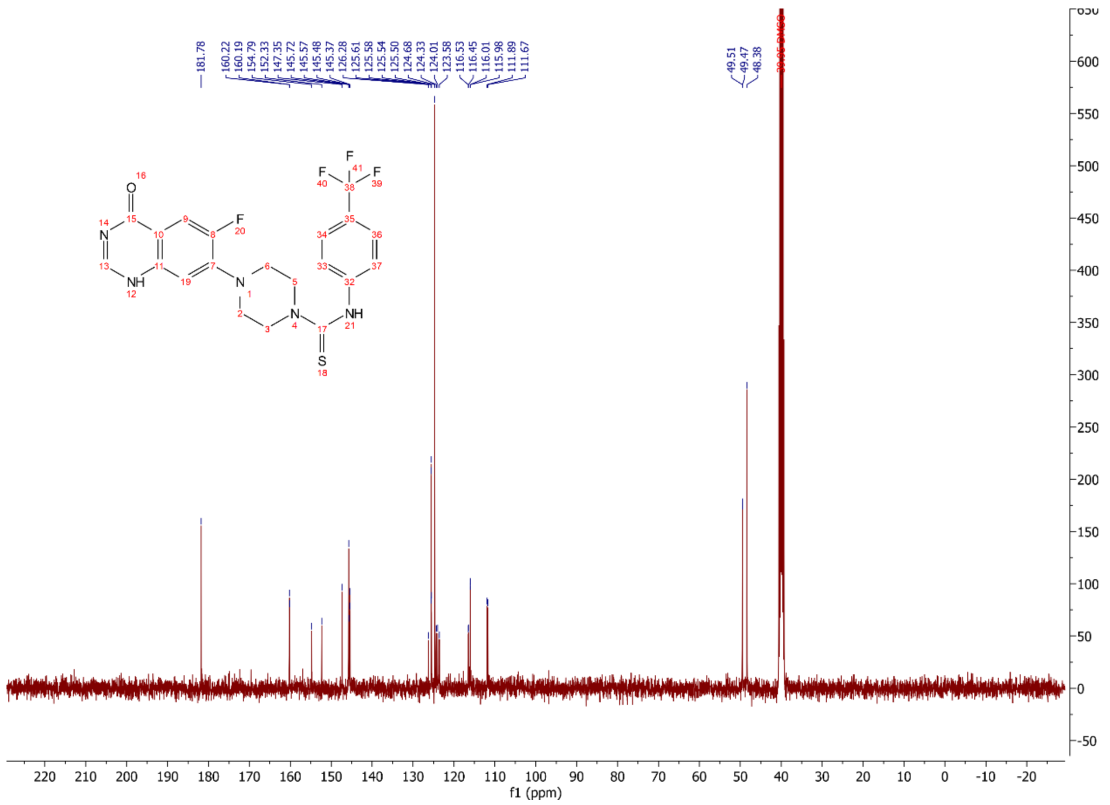

^{13}C NMR (101 MHz, DMSO-d_6) at 298 K of **27**.

Methyl 2-(6-fluoro-4-oxo-7-(4-((4-(trifluoromethyl)phenyl)carbamothioyl)piperazin-1-yl)quinazolin-3(4H)-yl)acetate (28)

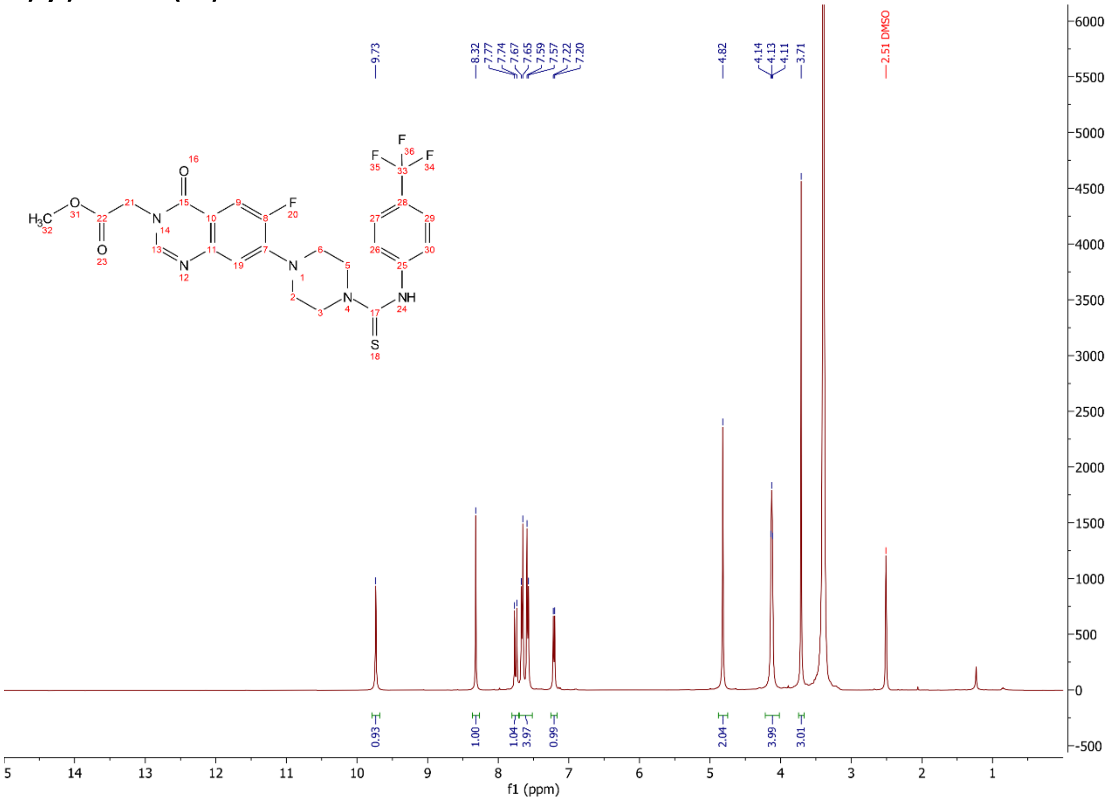

^{1}H NMR (400 MHz, DMSO-d_6) at 298 K of **28**.

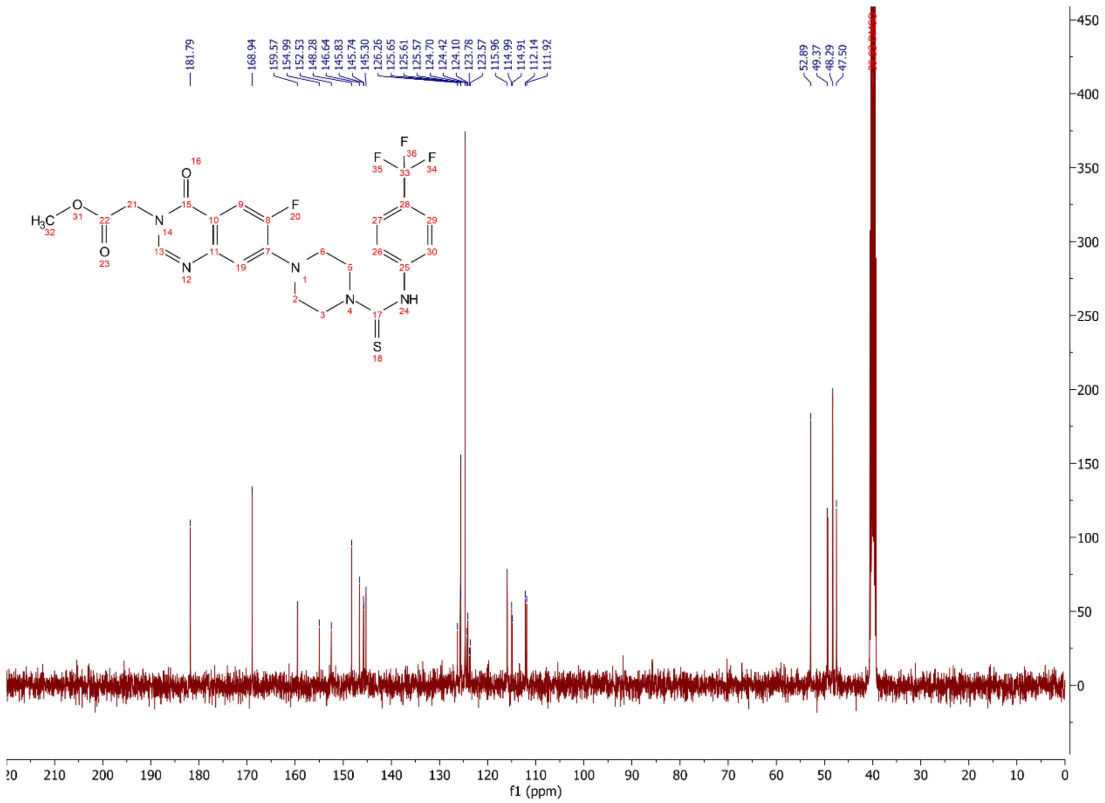

^{13}C NMR (101 MHz, DMSO-d_6) at 298 K of **28**.

4-(6-Fluoro-2,4-dioxo-1,2,3,4-tetrahydroquinazolin-7-yl)-N-(4-(trifluoromethyl)phenyl) piperazine-1-carbothioamide (29)

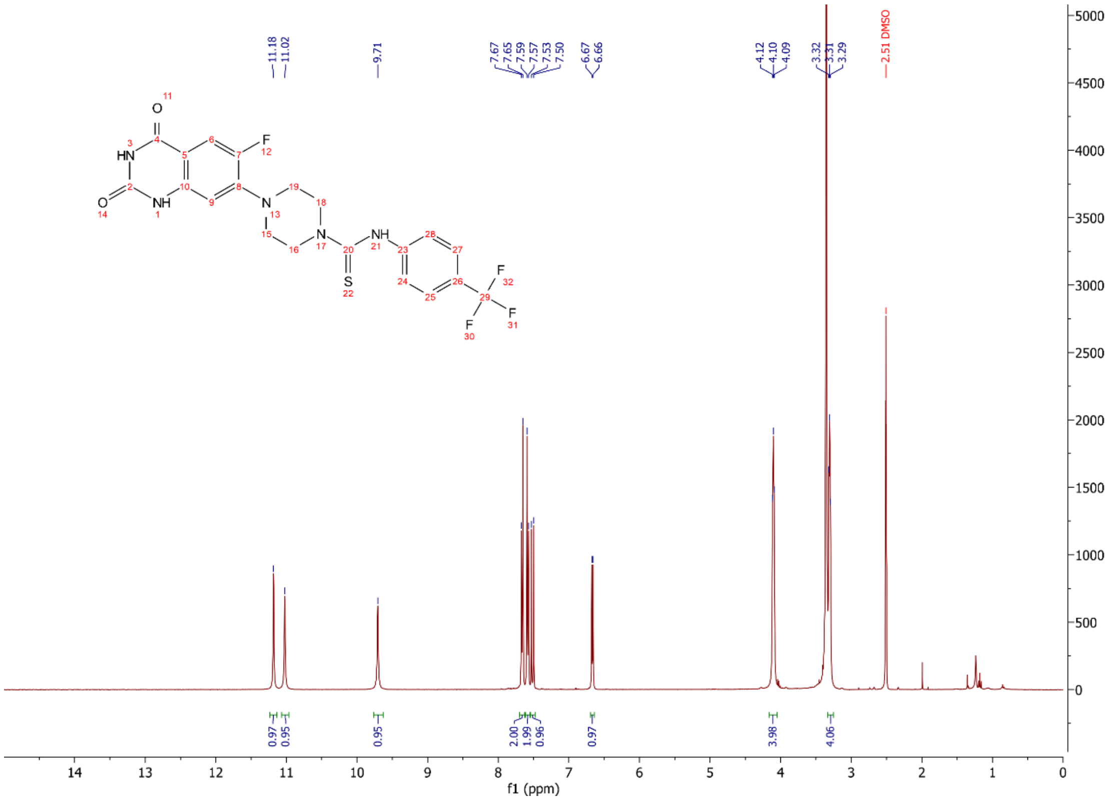

^{1}H NMR (400 MHz, DMSO-d_6) at 298 K of **29**.

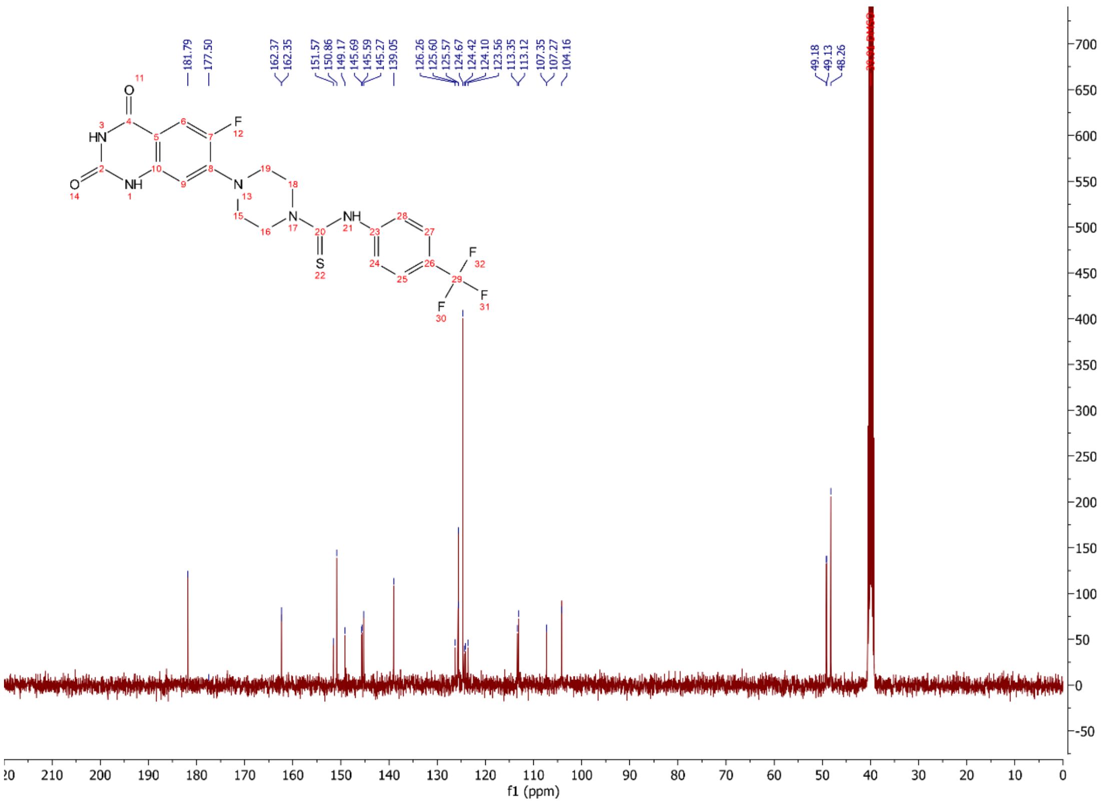

^{13}C NMR (101 MHz, DMSO-d_6) at 298 K of **29**.

Ethyl 2-(6-fluoro-2,4-dioxo-7-(4-((4-(trifluoromethyl)phenyl)carbamothioyl)piperazin-1-yl)-1,4-dihydroquinazolin-3(2H)-yl)acetate (30)

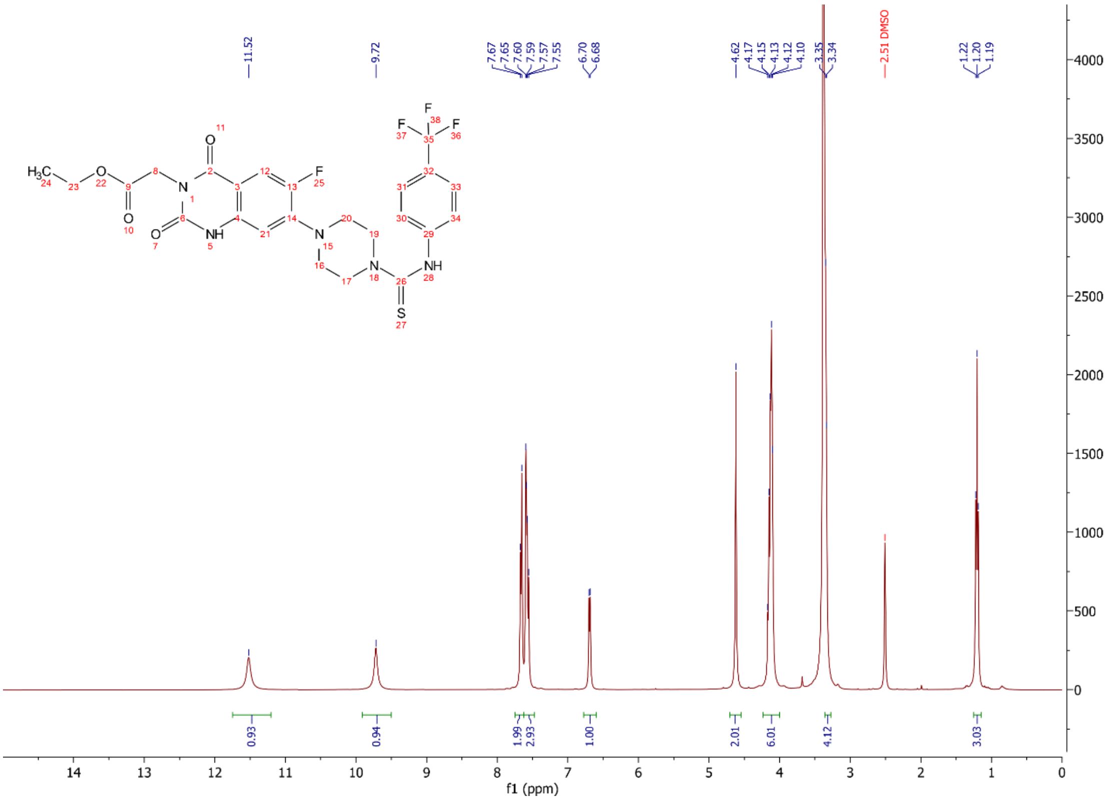

^{1}H NMR (400 MHz, DMSO-d_6) at 298 K of **30**.

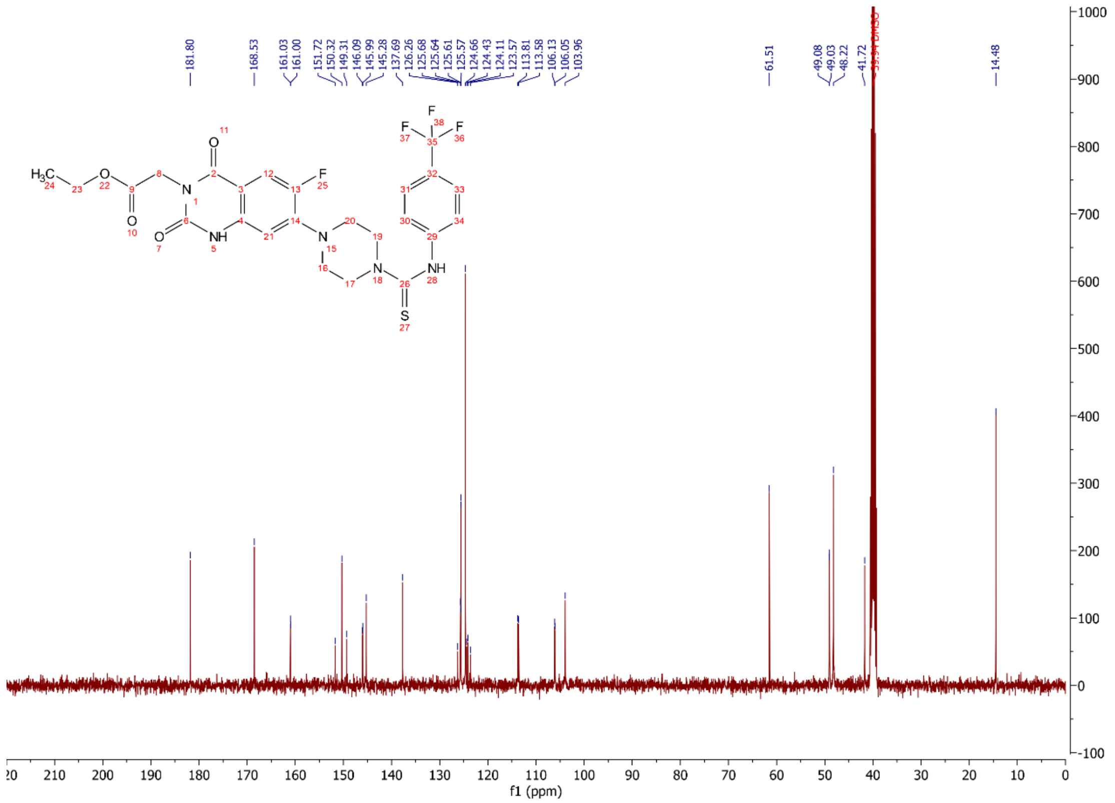

^{13}C NMR (101 MHz, DMSO-d_6) at 298 K of **30**.

2-(6-Fluoro-2,4-dioxo-7-(4-((4-(trifluoromethyl)phenyl)carbamothioyl)piperazin-1-yl)-1,4-dihydroquinazolin-3(2H)-yl)acetic acid (31)

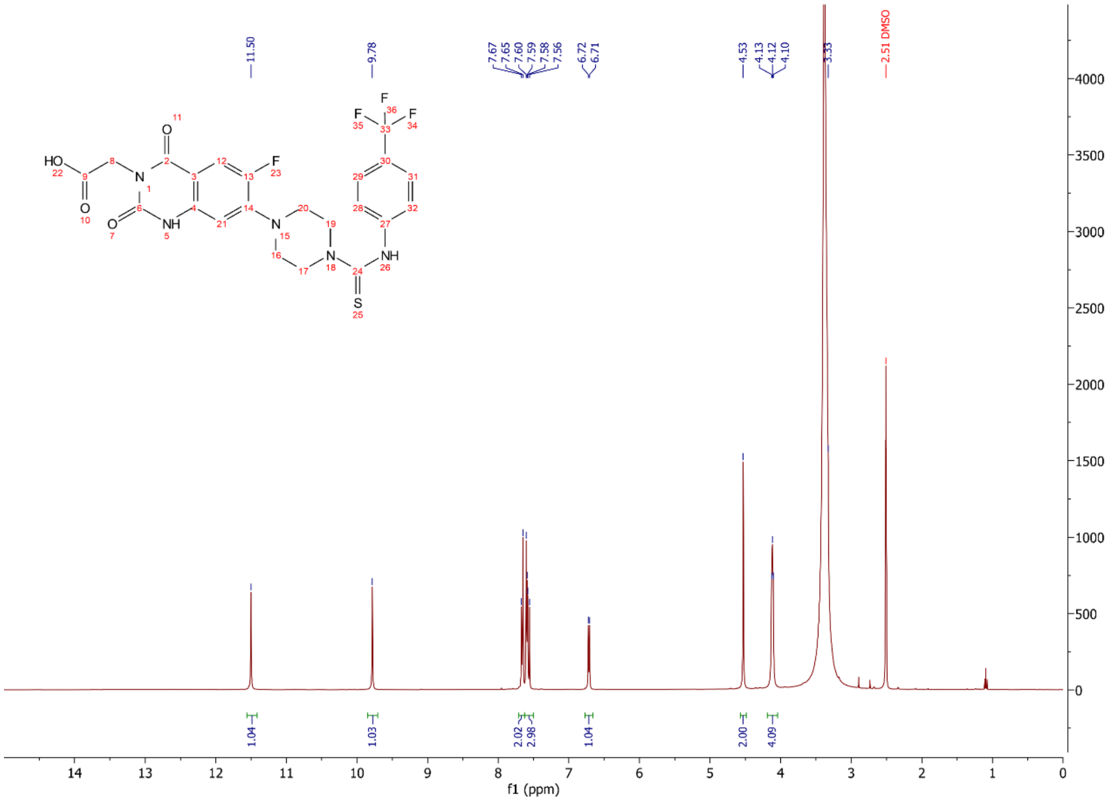

^{1}H NMR (400 MHz, DMSO-d_6) at 298 K of **31**.

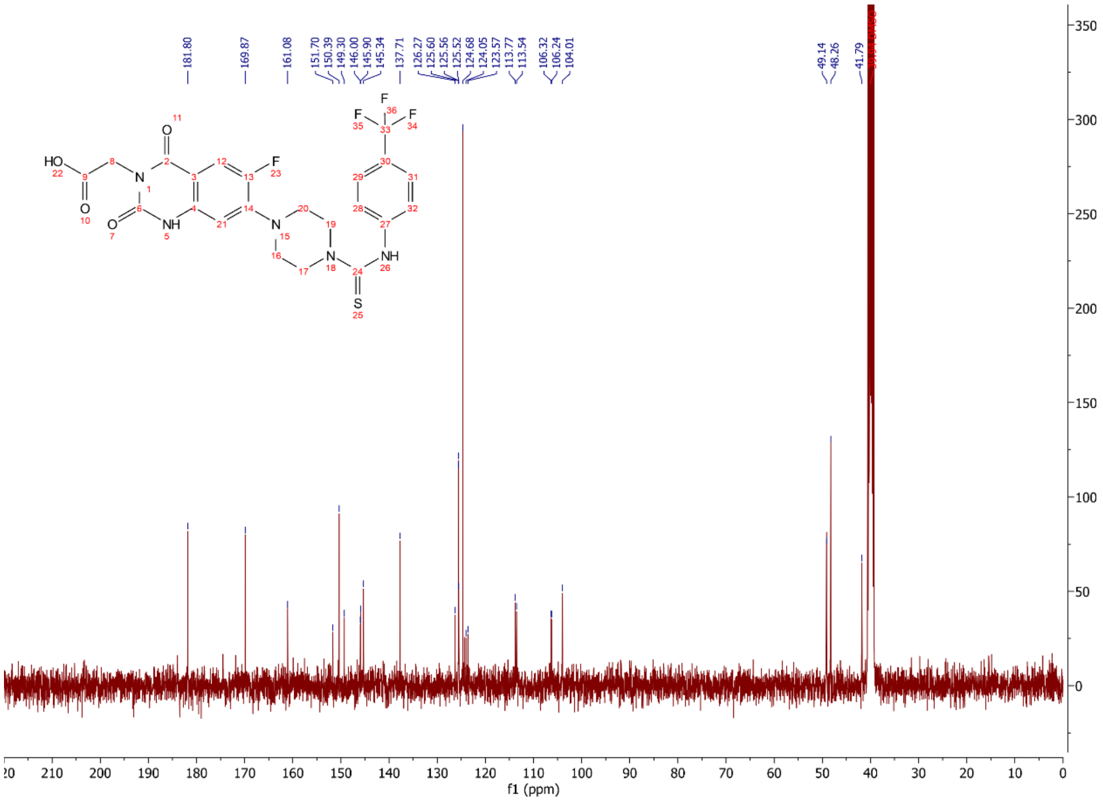

^{13}C NMR (101 MHz, DMSO-d_6) at 298 K of **31**.

2-(1-Ethyl-6-fluoro-2,4-dioxo-7-(4-((4-(trifluoromethyl)phenyl)carbamothioyl)piperazin-1-yl)-1,4-dihydroquinazolin-3(2H)-yl)acetic acid (32)

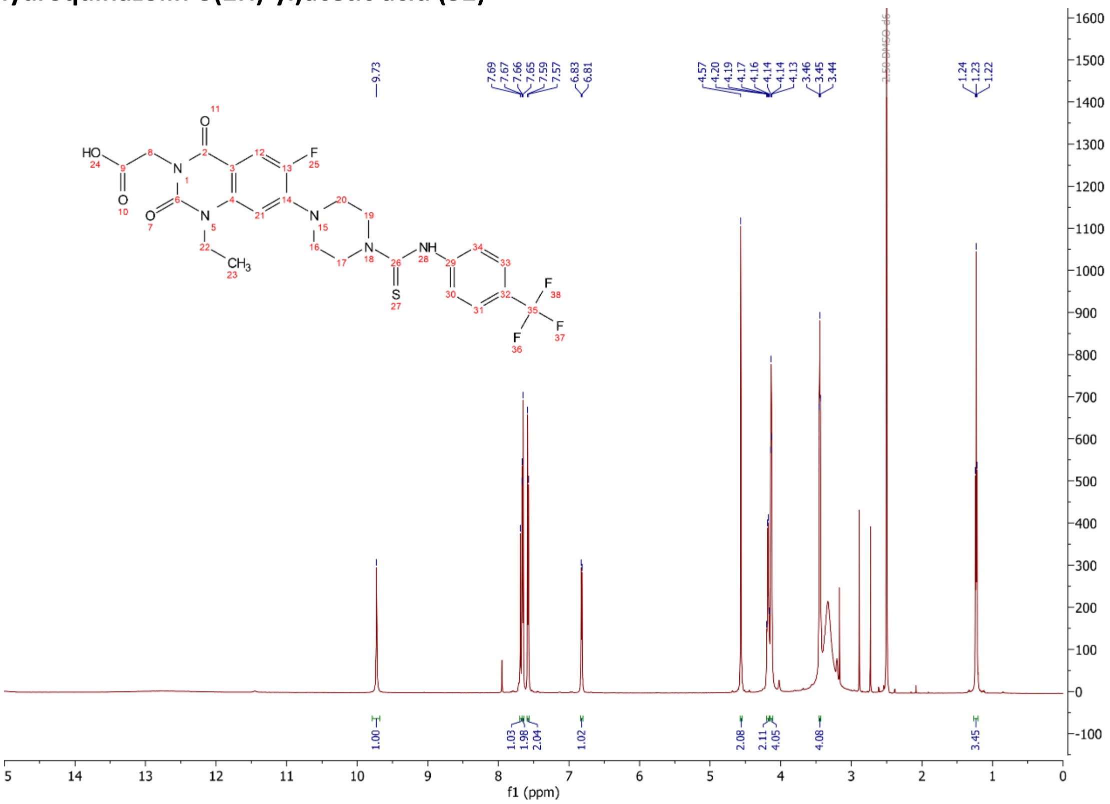

^{1}H NMR (600 MHz, DMSO-d_6) at 298 K of **32**.

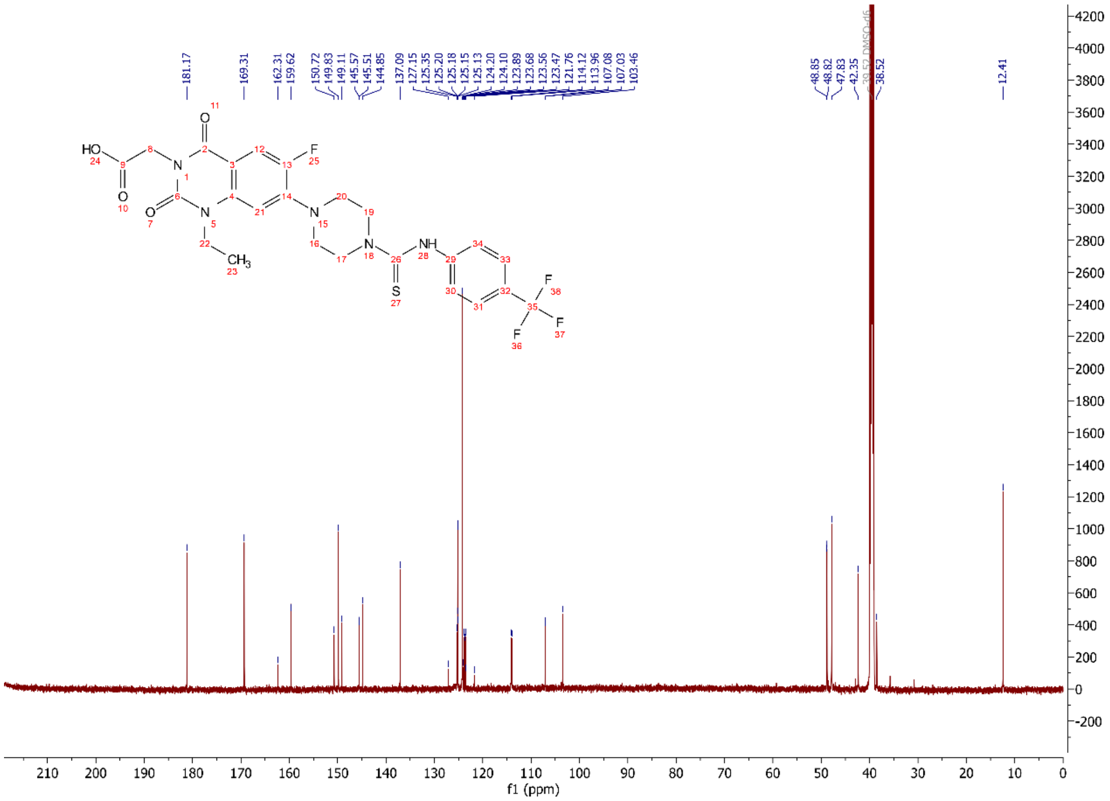

^{13}C NMR (151 MHz, DMSO-d_6) at 298 K of **32**.

Ethyl 1-cyclopropyl-6-fluoro-4-oxo-7-(4-((4-(trifluoromethyl)phenyl)carbamothioyl)piperazin-1-yl)-1,4-dihydroquinoline-3-carboxylate (33)

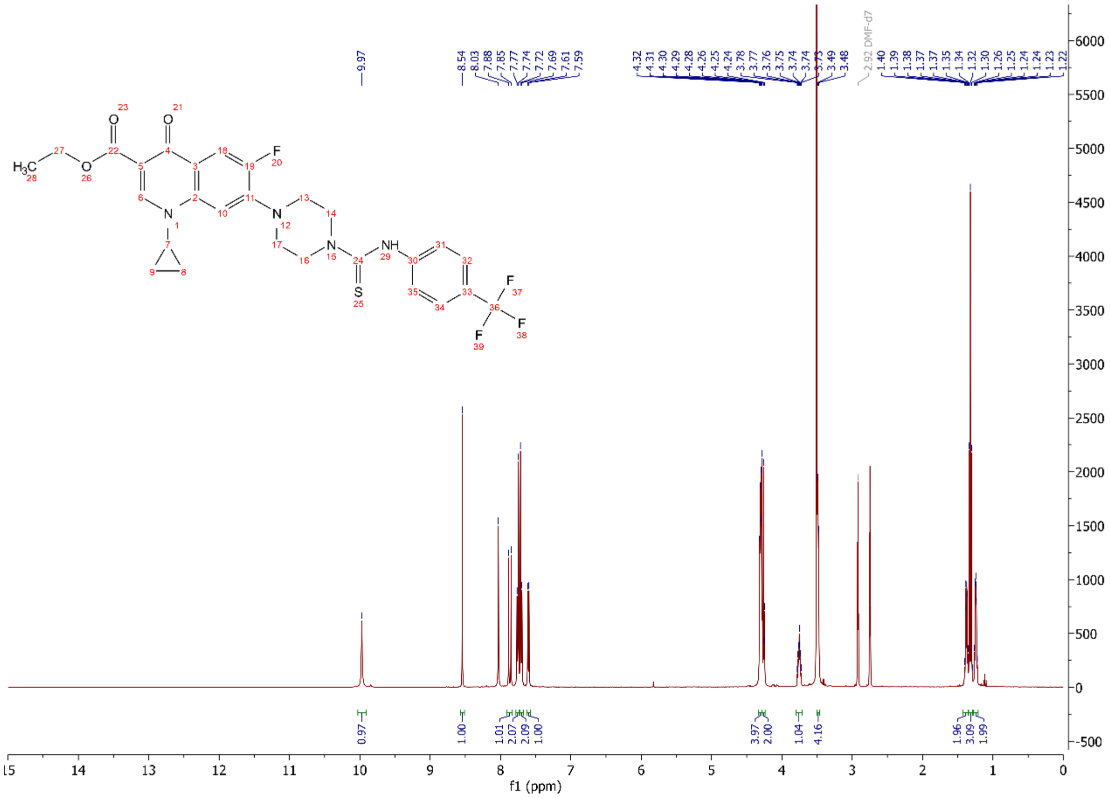

^{1}H NMR (400 MHz, DMF-d_7) at 298 K of **33**.

2-((1-Cyclopropyl-6-fluoro-4-oxo-7-(4-((4-(trifluoromethyl)phenyl) carbamothioyl) piperazin-1-yl)-1,4-dihydroquinoline-3-carbonyl)oxy) ethan-1-aminium chloride (34)

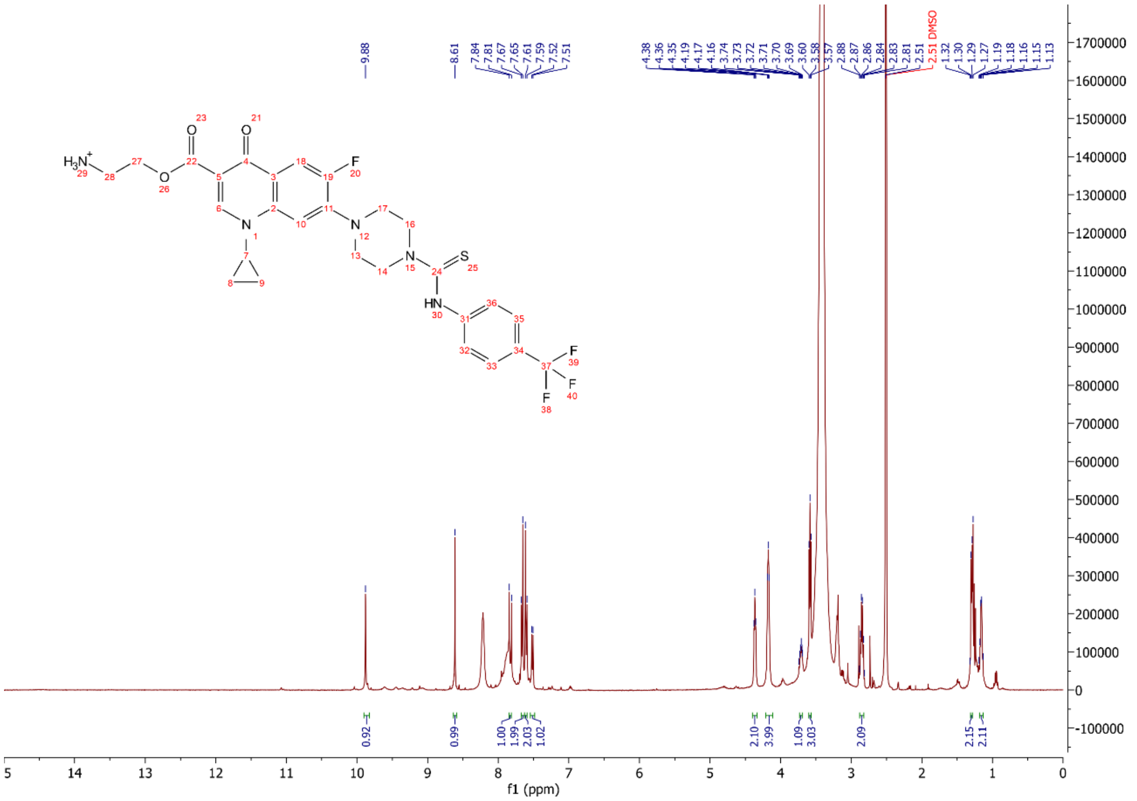

^{1}H NMR (400 MHz, DMSO-d_6) at 298 K of **34**.

1-Cyclopropyl-6-fluoro-*N* -(2-hydroxyethyl)-4-oxo-7-(4-((4-(trifluoromethyl) phenyl)carbamothioyl)piperazin-1-yl)-1,4-dihydroquinoline-3-carboxamide (35)

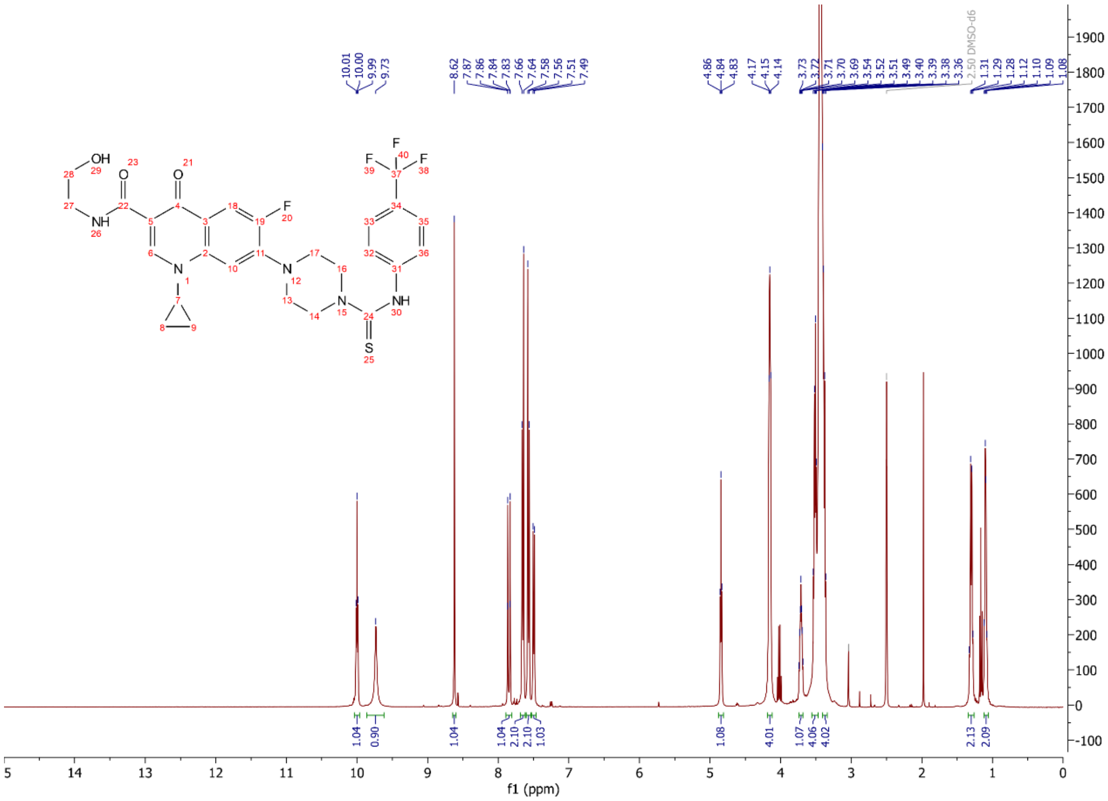

^{1}H NMR (400 MHz, DMSO-d_6) at 298 K of **35**.

1-Benzyl-6-fluoro-4-oxo-7-(4-((4-(trifluoromethyl)phenyl)carbamothioyl)piperazin-1-yl)-1,4-dihydroquinoline-3-carboxylic acid (36)

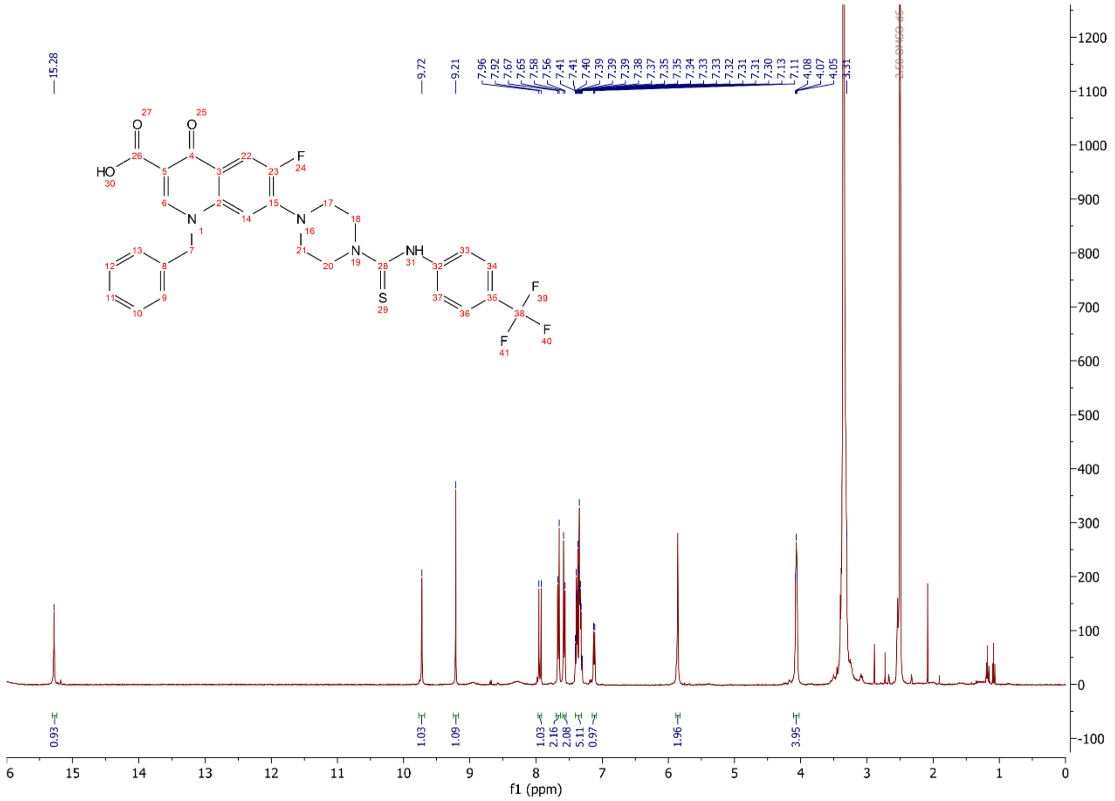

^{1}H NMR (400 MHz, DMSO-d_6) at 298 K of **36**.

Ethyl 6-fluoro-1-(4-fluorobenzyl)-4-oxo-7-(piperazin-1-yl)-1,4-dihydroquinoline-3-carboxylate (37)

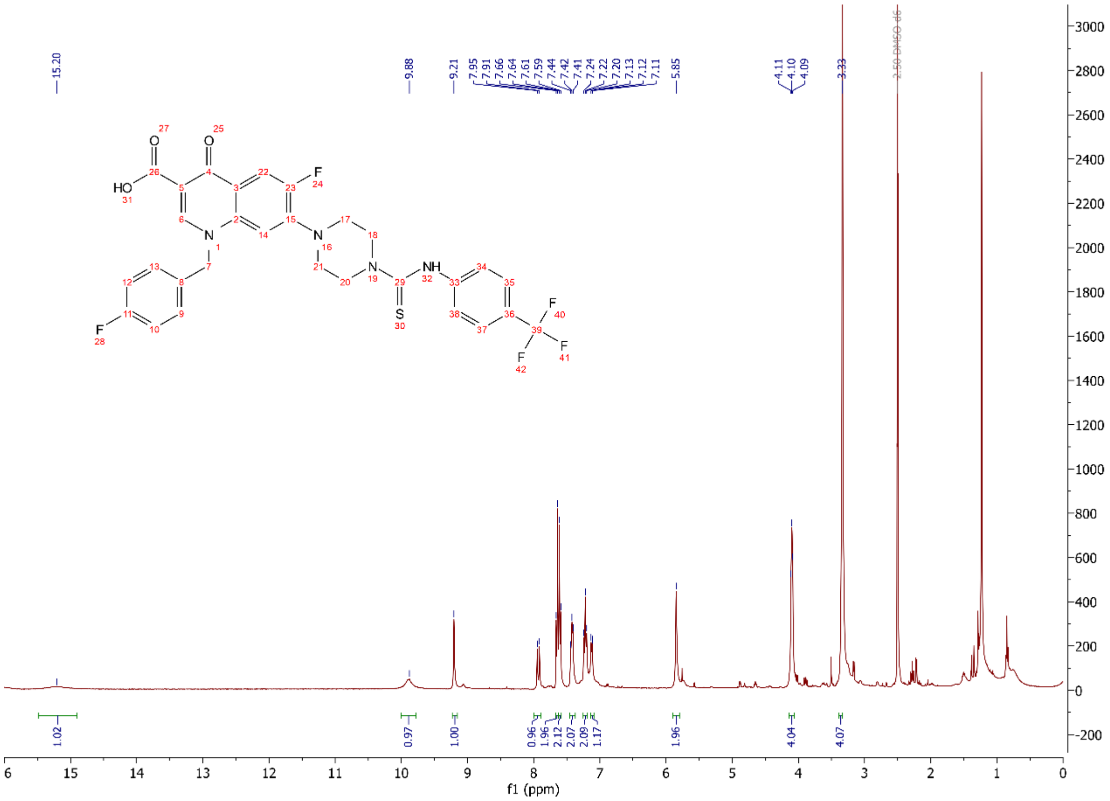

^{1}H NMR (400 MHz, DMSO-d_6) at 298 K of **37**.

1-Ethyl-6-fluoro-4-oxo-7-(4-((4-(trifluoromethyl)phenyl)carbamothioyl)piperazin-1-yl)-1,4-dihydroquinoline-3-carboxylic acid (38)

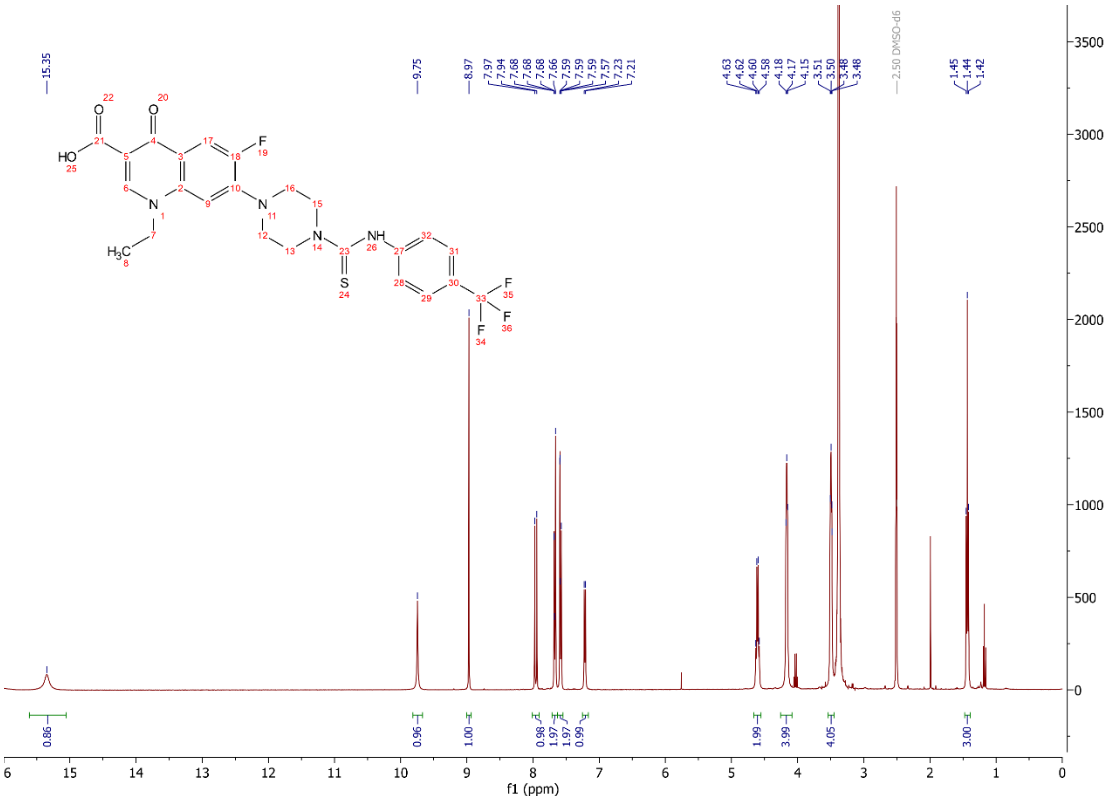

^{1}H NMR (400 MHz, DMSO-d_6) at 298 K of **38**.

6-Fluoro-1-isopropyl-4-oxo-7-(4-((4-(trifluoromethyl)phenyl)carbamothioyl)piperazin-1-yl)-1,4-dihydroquinoline-3-carboxylic acid (39)

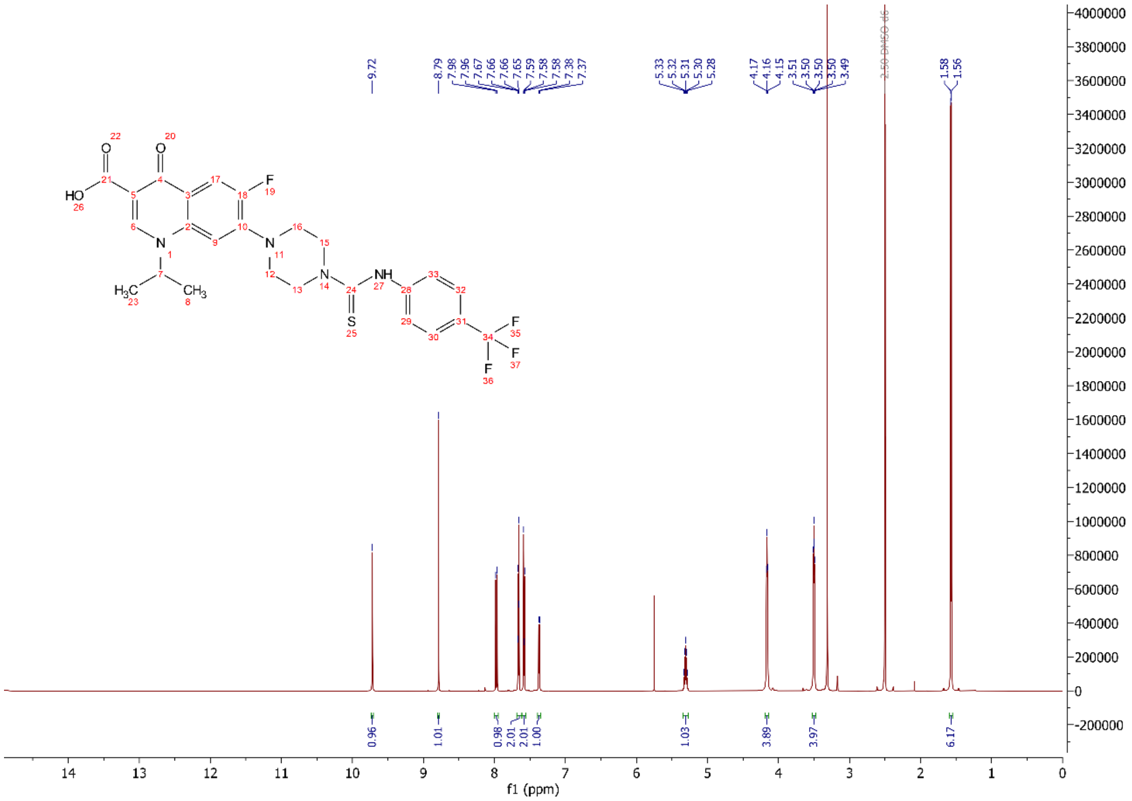

^{1}H NMR (600 MHz, DMSO-d_6) at 298 K of **39**.

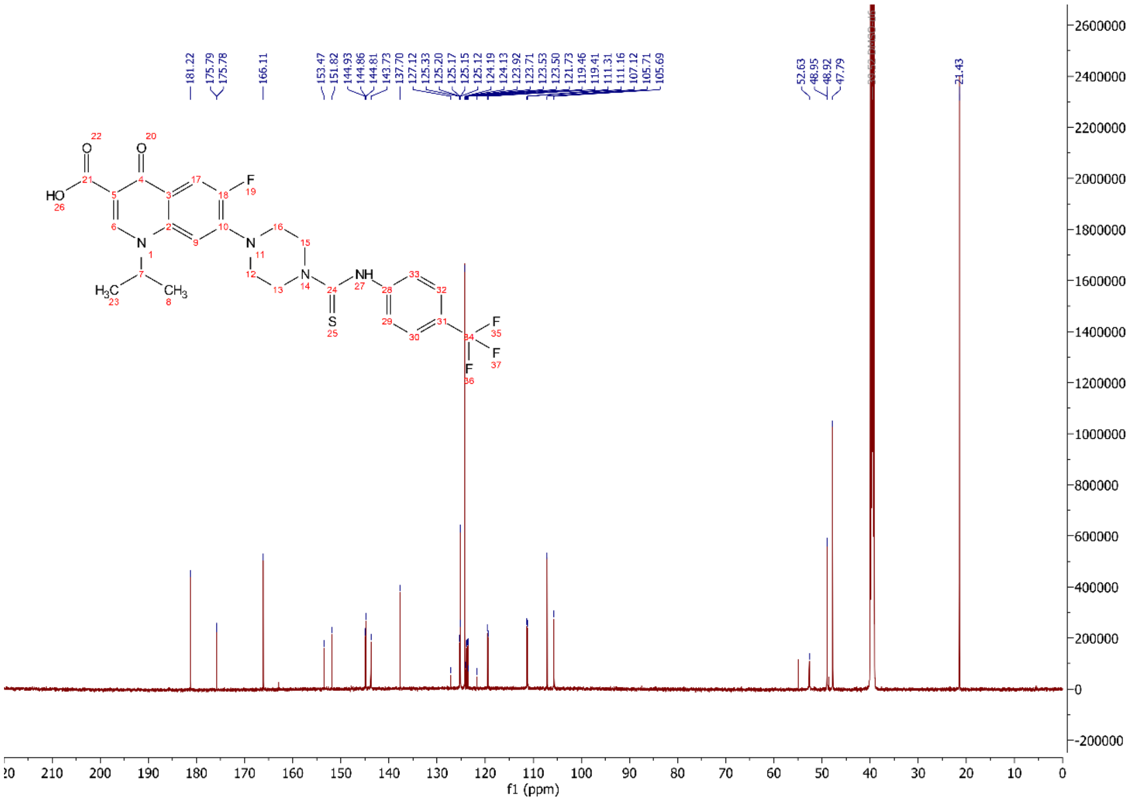

^{13}C NMR (151 MHz, DMSO-d_6) at 298 K of **39**.

6-Fluoro-1-isopropyl-4-oxo-7-(4-((4-(trifluoromethyl)phenyl)carbamoyl)piperazin-1-yl)-1,4-dihydroquinoline-3-carboxylic acid (40)

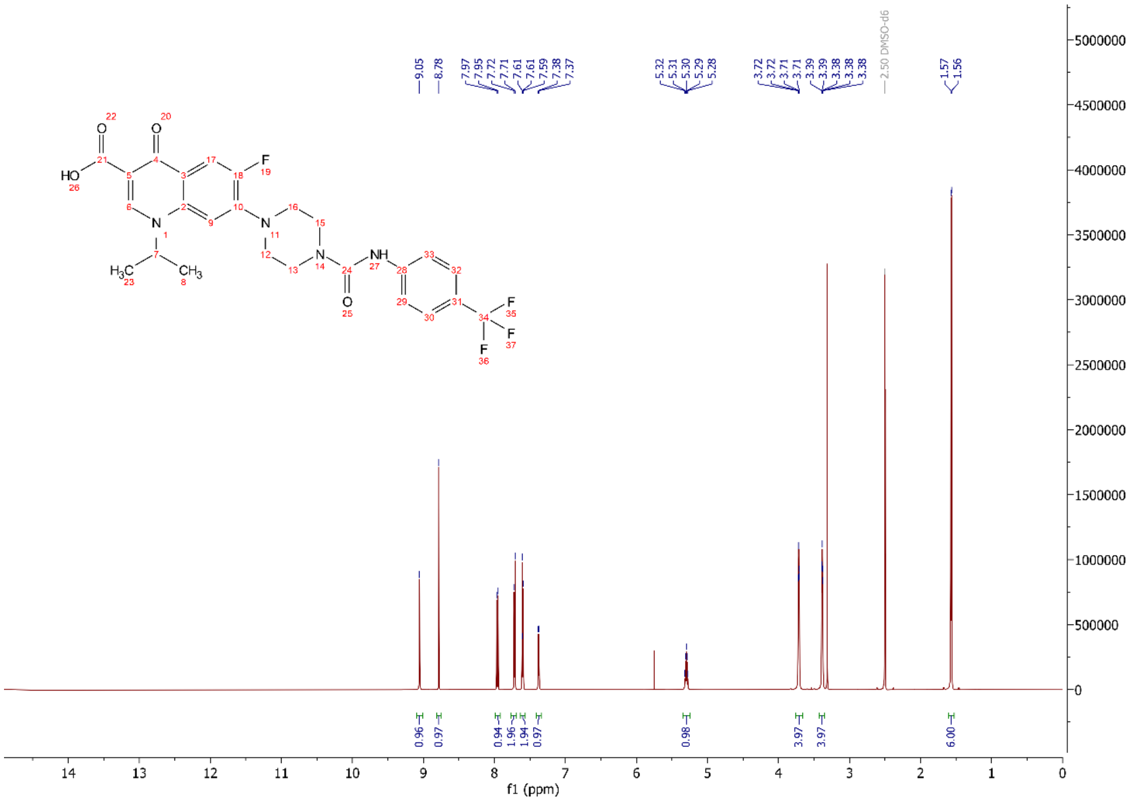

^{1}H NMR (600 MHz, DMSO-d_6) at 298 K of **40**.

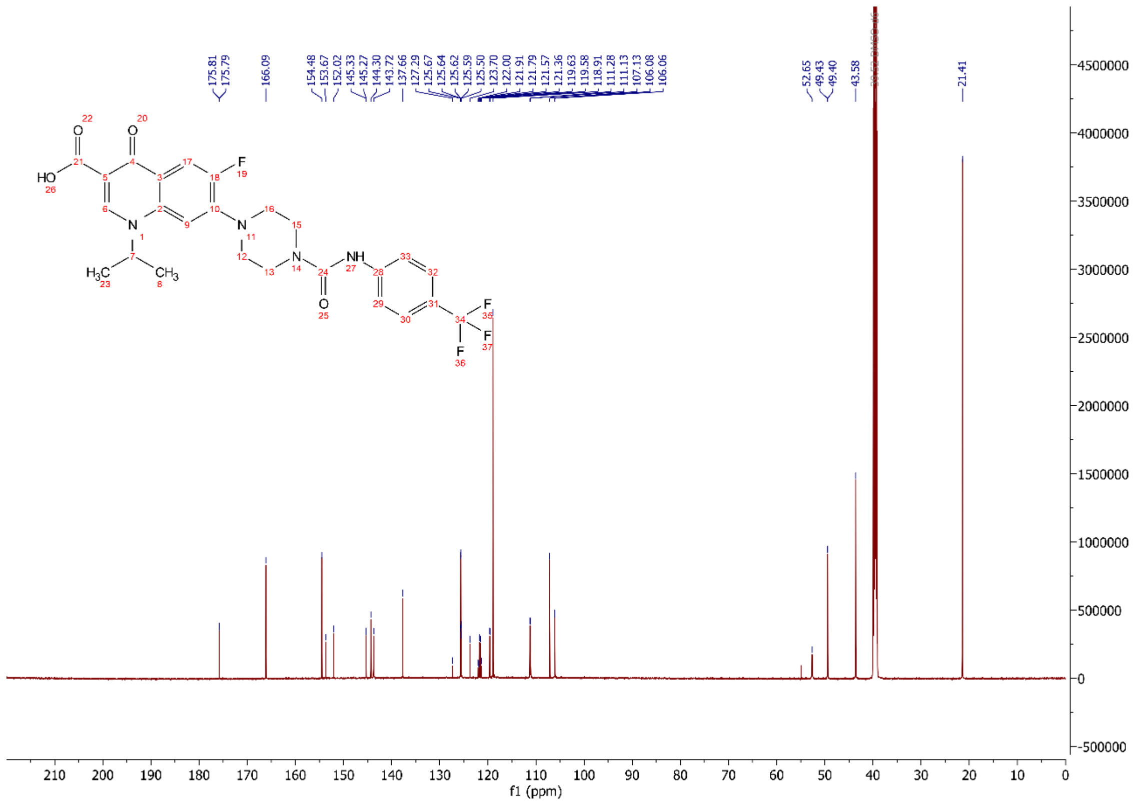

^{13}C NMR (151 MHz, DMSO-d_6) at 298 K of **40**.

HPLC Traces of Key Compounds

8-Ethyl-5-oxo-2-(4-((3-(trifluoromethyl)phenyl)carbamothioyl)piperazin-1-yl)-5,8-dihydropyrido[2,3-*d*]pyrimidine-6-carboxylic acid (ML328, 2)

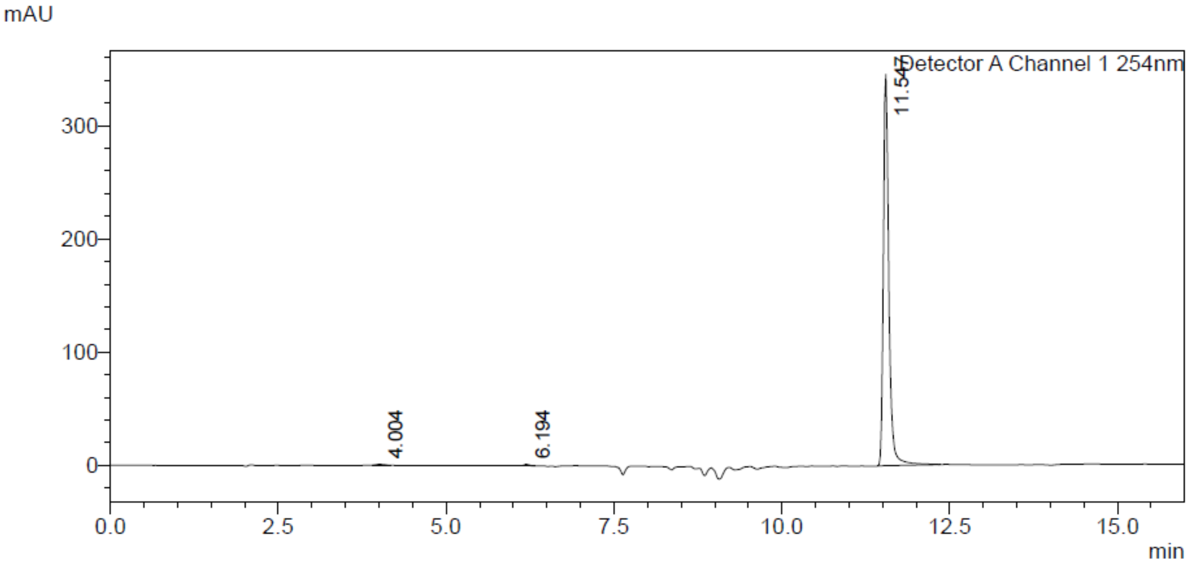

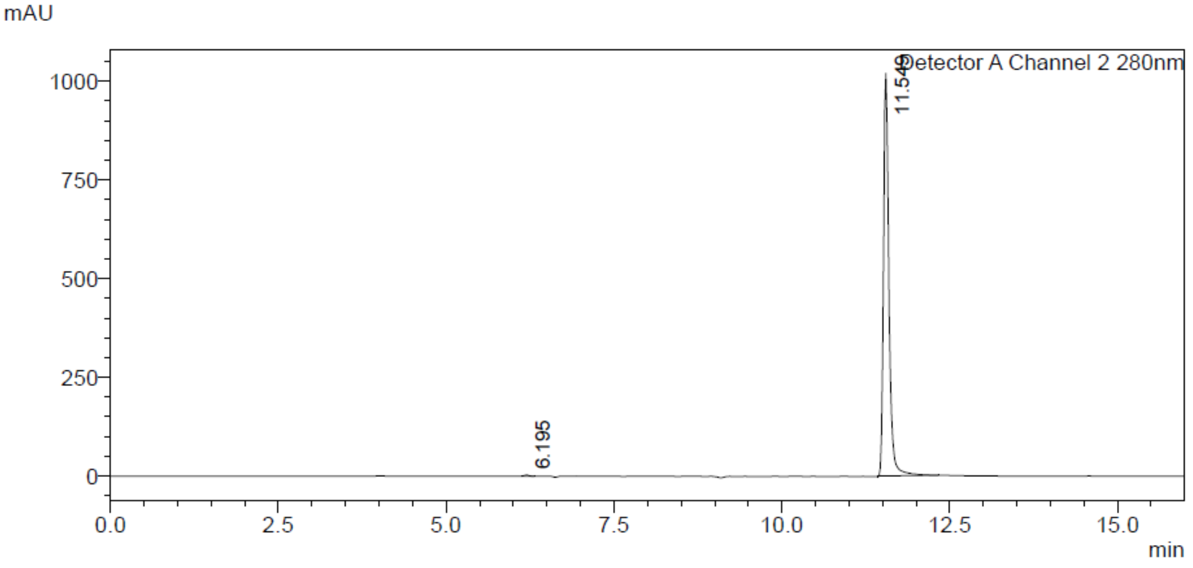

Detector A Channel 1 254 nm

Peak#	Ret. Time	Area	Height	Conc.	Unit	Mark	Name
1	4.004	6595	752	0.332		M	
2	6.194	4711	1069	0.237		M	
3	11.547	1973546	346132	99.430		M	
Total		1984852	347953				

Detector A Channel 2 280nm

Peak#	Ret. Time	Area	Height	Conc.	Unit	Mark	Name
1	6.195	13025	2953	0.225		M	
2	11.549	5779116	1020531	99.775		M	
Total		5792141	1023484				

1-Cyclopropyl-6-fluoro-4-oxo-7-(4-((4-(trifluoromethyl)phenyl)carbamothioyl) piperazin-1-yl)-1,4-dihydroquinoline-3-carboxylic acid (IMP-1700, 3)

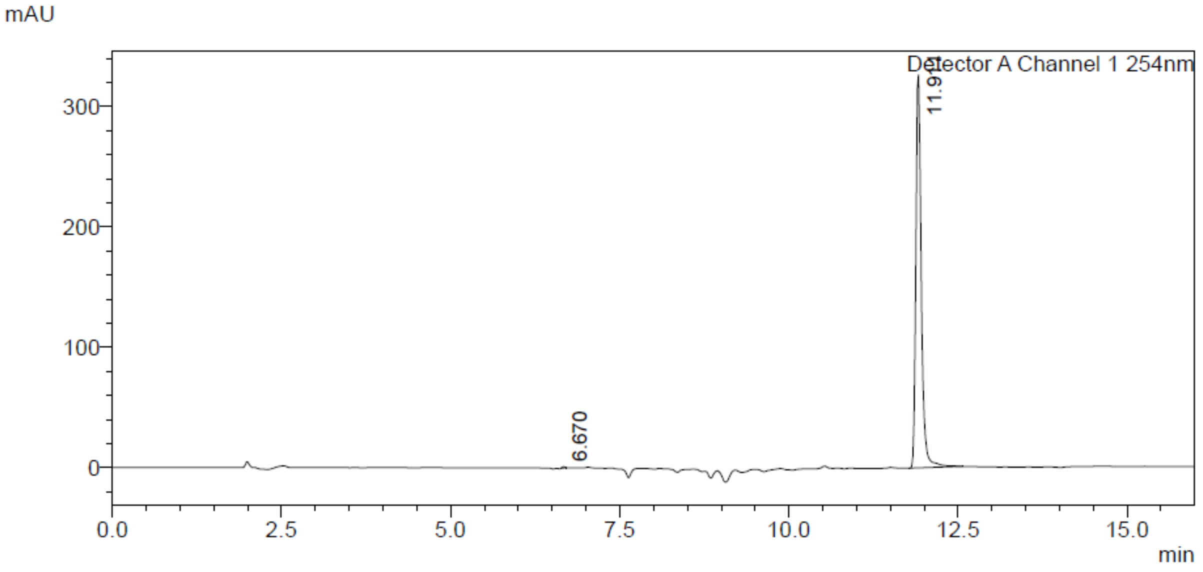

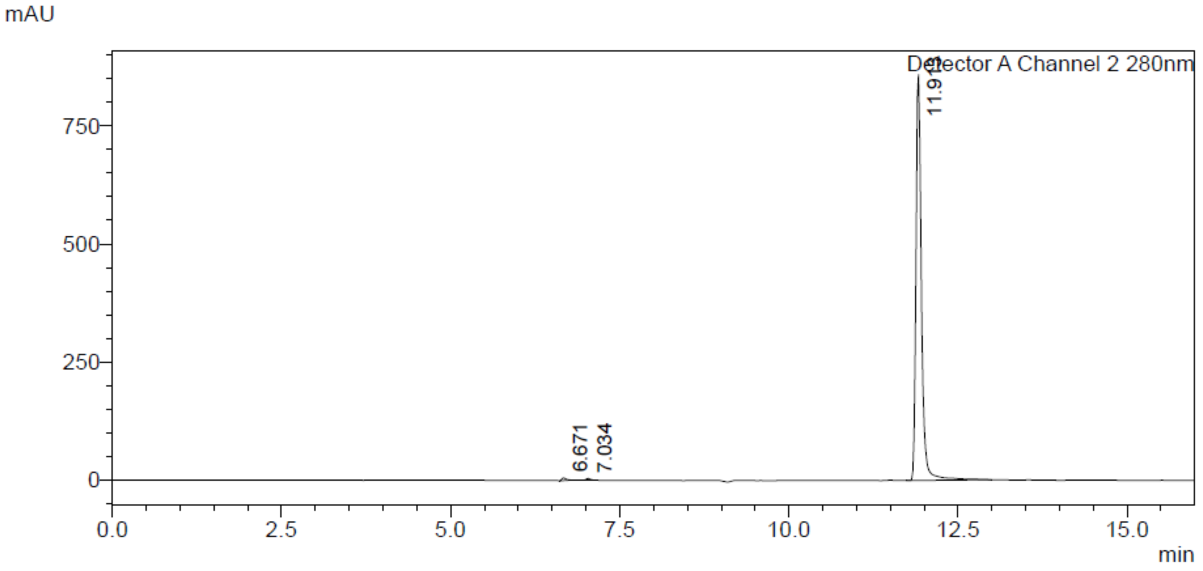

Detector A Channel 1 254 nm

Peak#	Ret. Time	Area	Height	Conc.	Unit	Mark	Name
1	6.670	2891	948	0.156		M	
2	11.911	1846981	327004	99.844		M	
Total		1849872	327952				

Detector A Channel 2 280nm

Peak#	Ret. Time	Area	Height	Conc.	Unit	Mark	Name
1	6.671	28697	6096	0.580		M	
2	7.034	12974	3091	0.262		M	
3	11.913	4905674	860019	99.158		M	
Total		4947345	869206				

1-Cyclopropyl-6-fluoro-4-oxo-7-(4-((4-(trifluoromethyl)phenyl)carbamoyl)piperazin-1-yl)-1,4-dihydroquinoline-3-carboxylic acid (11)

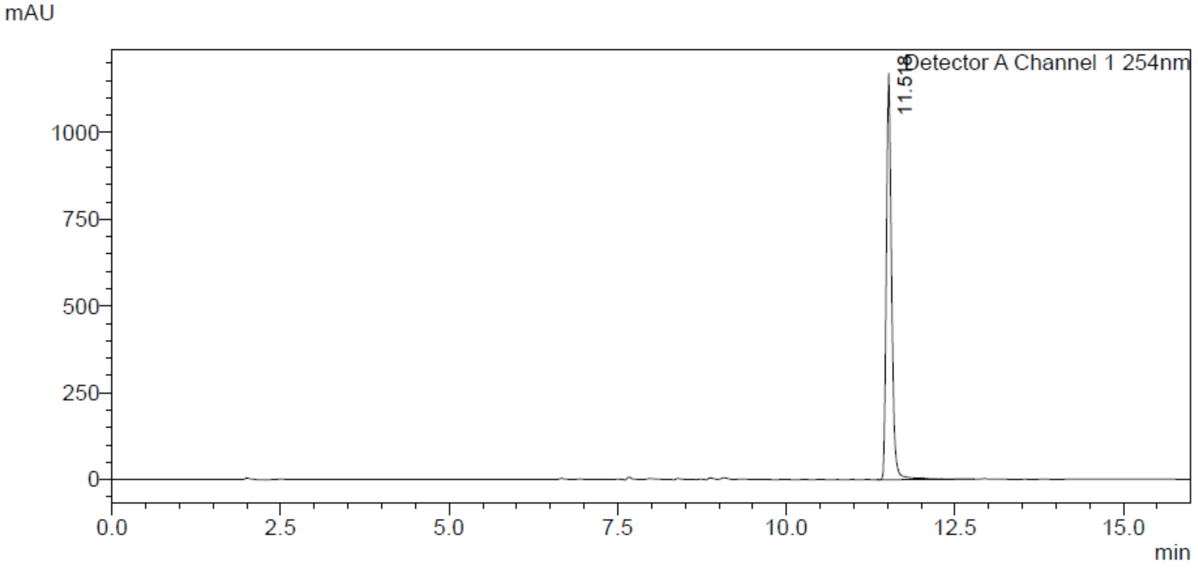

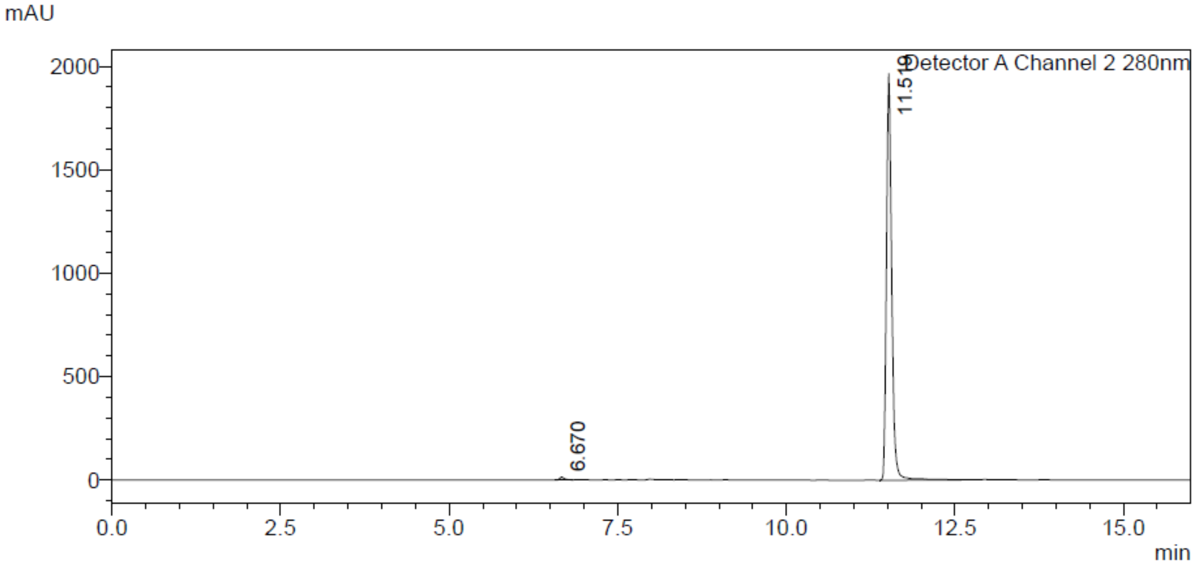

Detector A Channel 1 254 nm

Peak#	Ret. Time	Area	Height	Conc.	Unit	Mark	Name
1	11.518	6365882	1171945	100.000		M	
Total		6365882	1171945				

Detector A Channel 2 280nm

Peak#	Ret. Time	Area	Height	Conc.	Unit	Mark	Name
1	6.670	63326	13682	0.590		M	
2	11.519	10671247	1967119	99.410		M	
Total		10734573	1980801				

Ethyl 1-cyclopropyl-6-fluoro-4-oxo-7-(4-((4-(trifluoromethyl)phenyl)carbamothioyl)piperazin-1-yl)-1,4-dihydroquinoline-3-carboxylate (33)

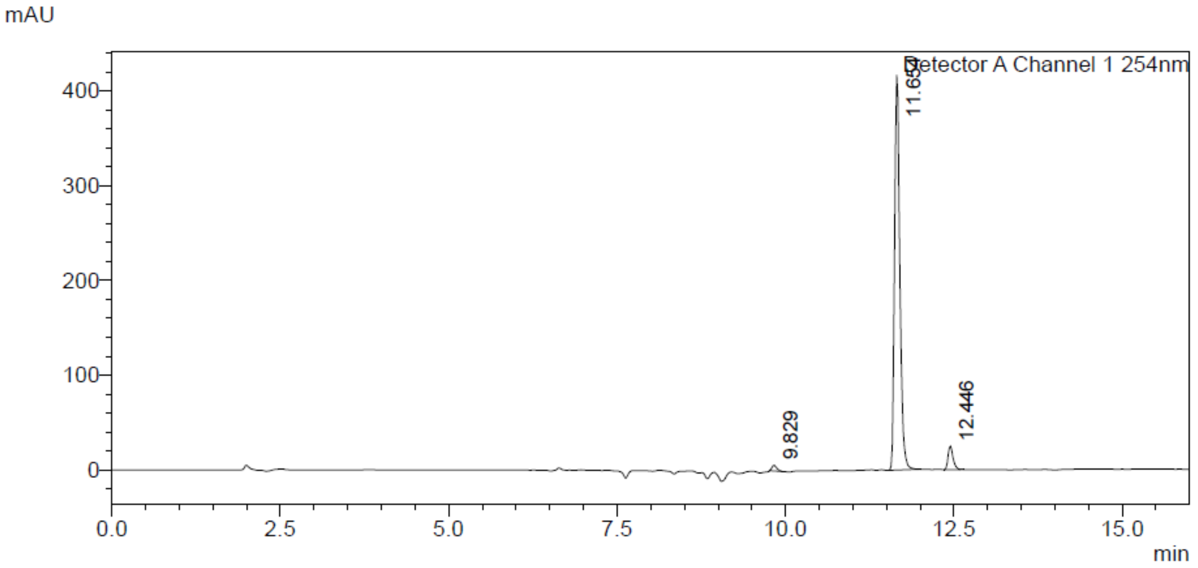

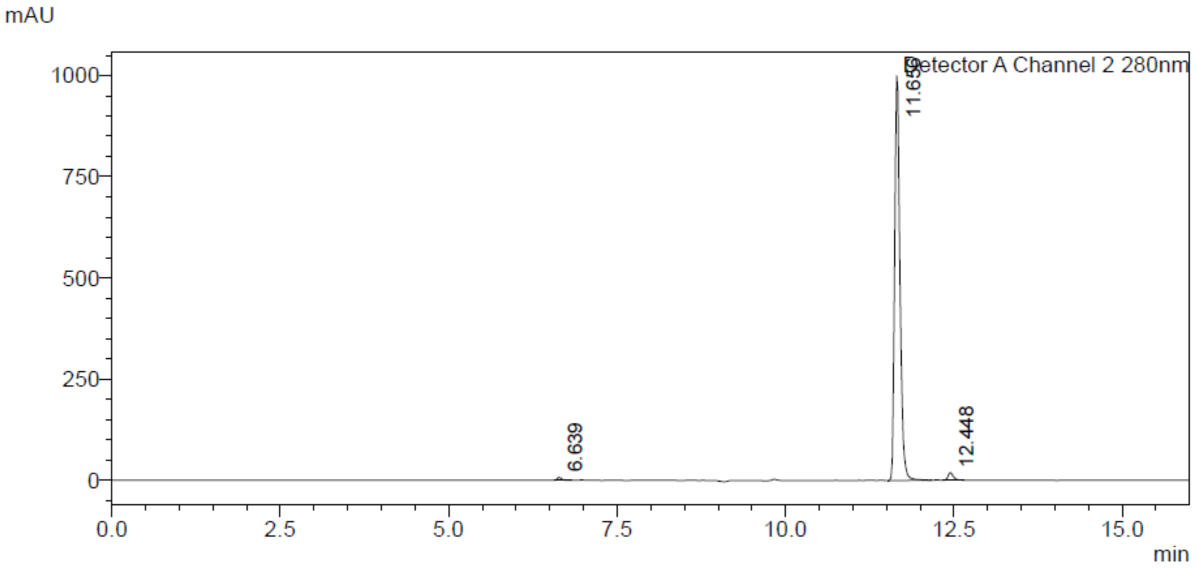

Detector A Channel 1 254 nm

Peak#	Ret. Time	Area	Height	Conc.	Unit	Mark	Name
1	9.829	29651	5890	1.221		M	
2	11.654	2269649	416773	93.468		M	
3	12.446	128956	24695	5.311		M	
Total		2428256	447359				

Detector A Channel 2 280nm

Peak#	Ret. Time	Area	Height	Conc.	Unit	Mark	Name
1	6.639	33310	7123	0.593		M	
2	11.656	5491046	1001018	97.745		M	
3	12.448	93396	17958	1.663		M	
Total		5617752	1026099				

6-Fluoro-1-isopropyl-4-oxo-7-(4-((4-(trifluoromethyl)phenyl)carbamothioyl)piperazin-1-yl)-1,4-dihydroquinoline-3-carboxylic acid (OXF-077, 39)

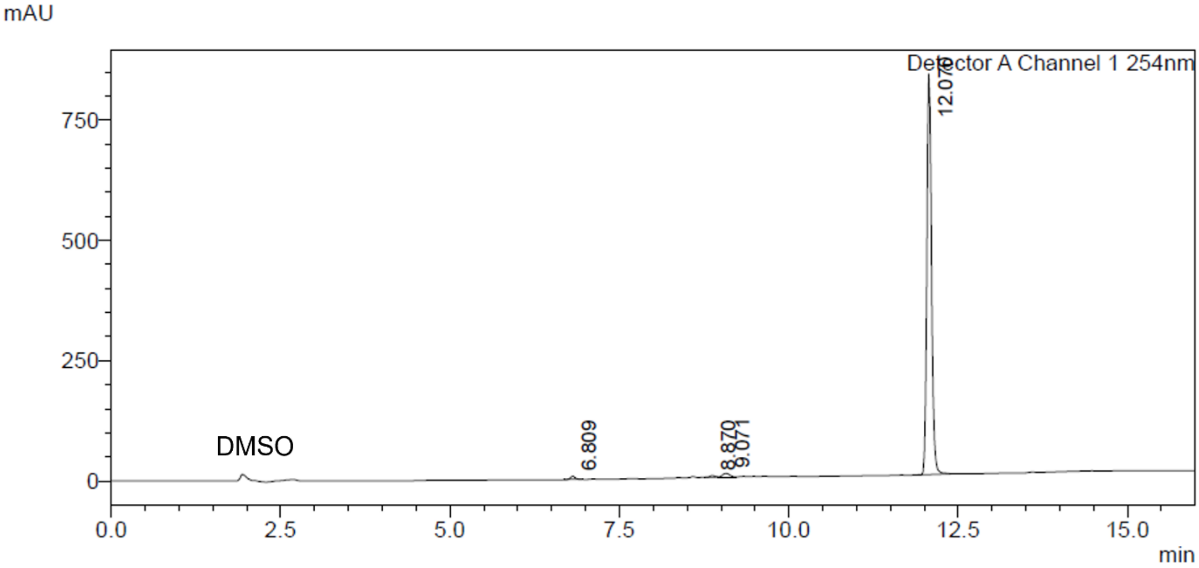

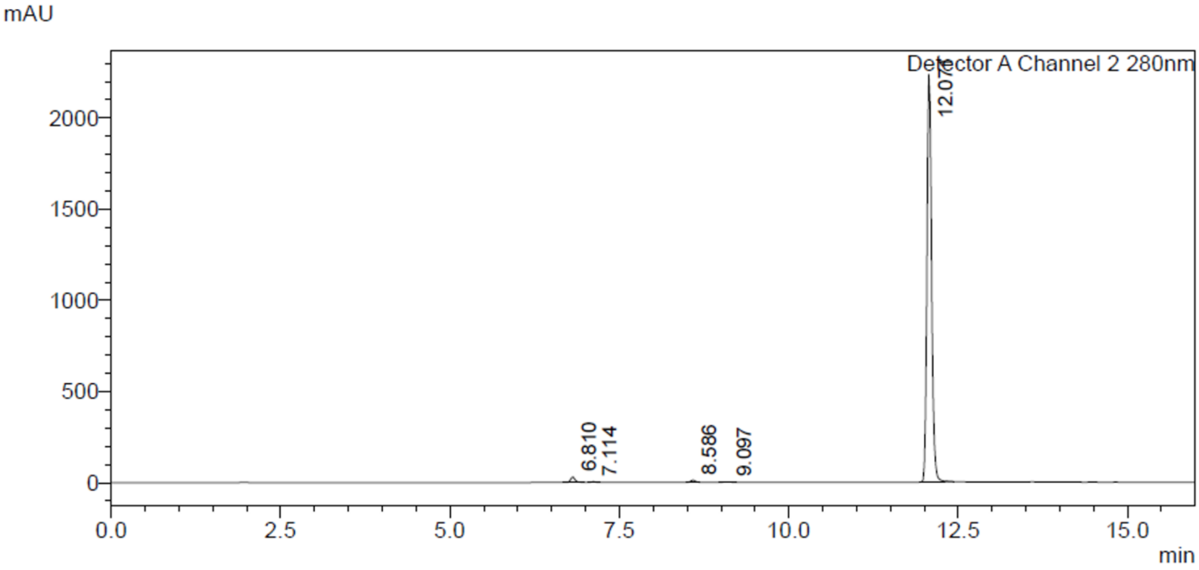

Detector A Channel 1 254 nm

Peak#	Ret. Time	Area	Height	Conc.	Unit	Mark	Name
1	6.809	31625	6560	0.765		M	
2	8.870	16683	3578	0.404		M	
3	9.071	51769	7485	1.253		M	
4	12.070	4032914	833703	97.579		M	
Total		4132991	851327				

Detector A Channel 2 280nm

Peak#	Ret. Time	Area	Height	Conc.	Unit	Mark	Name
1	6.810	139374	29107	1.263		M	
2	7.114	18273	4406	0.166		M	
3	8.586	59785	12839	0.542		M	
4	9.097	17537	2568	0.159		M	
5	12.071	10799472	2238465	97.871		M	
Total		11034441	2287384				

6-Fluoro-1-isopropyl-4-oxo-7-(4-((4-(trifluoromethyl)phenyl)carbamoyl)piperazin-1-yl)-1,4-dihydroquinoline-3-carboxylic acid (40)

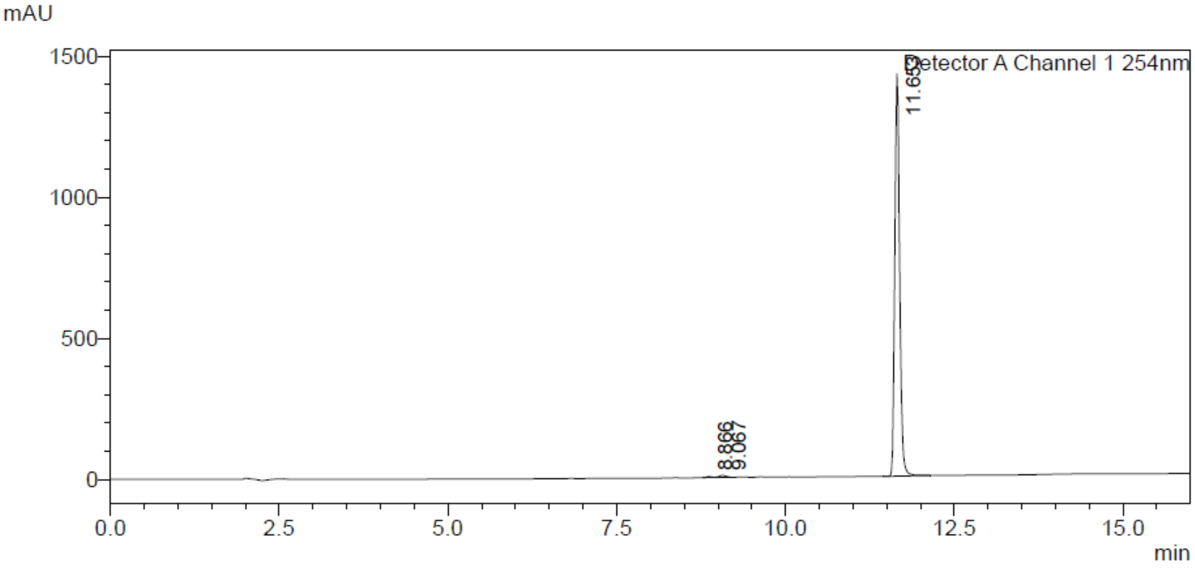

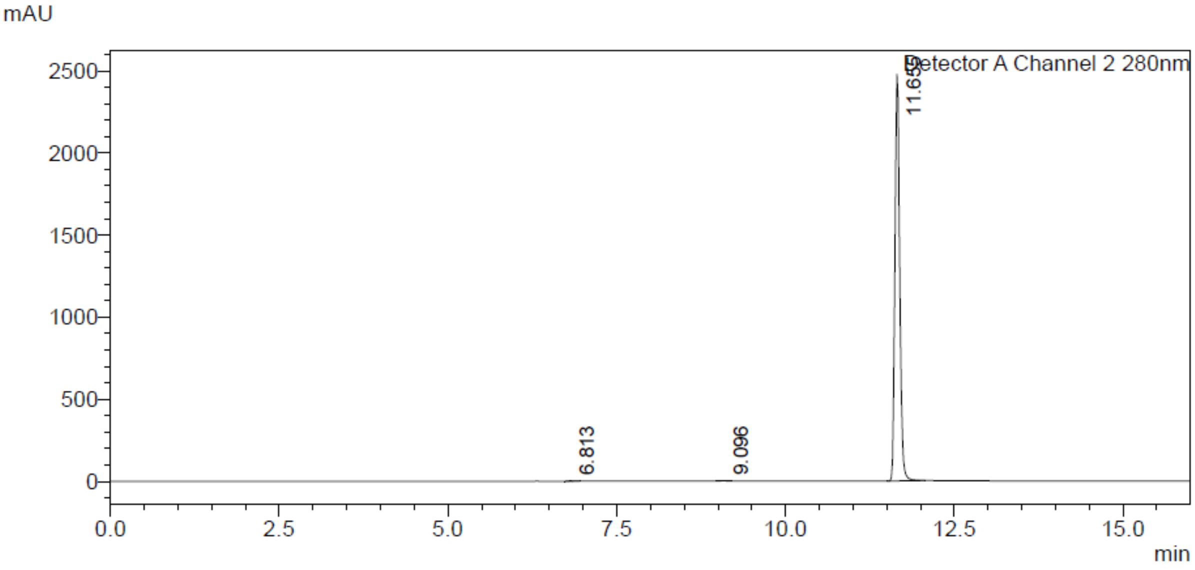

Detector A Channel 1 254 nm

Peak#	Ret. Time	Area	Height	Conc.	Unit	Mark	Name
1	8.866	13538	2955	0.189		M	
2	9.067	43978	6332	0.614		M	
3	11.653	7101033	1426619	99.197		M	
Total		7158549	1435906				

Detector A Channel 2 280nm

Peak#	Ret. Time	Area	Height	Conc.	Unit	Mark	Name
1	6.813	12992	2929	0.106		M	
2	9.096	13306	1948	0.109		M	
3	11.655	12205104	2480151	99.785		M	
Total		12231402	2485028				

References

1 N. Paesano, S. Marzocco, C. Vicidomini, C. Saturnino, G. Autore, G. De Martino and G. Sbardella, *Bioorg Med Chem Lett*, 2005, **15**, 539–543.

2 P. D. Fey, J. L. Endres, V. K. Yajjala, T. J. Widhelm, R. J. Boissy, J. L. Bose and K. W. Bayles, *mBio*, 2013, **4**, 2161–2129.

3 R. S. Clarke, M. S. Bruderer, K. P. Ha and A. M. Edwards, *Antimicrob Agents Chemother*, 2019, **63**, e00944-19.

4 M. J. Horsburgh, J. L. Aish, I. J. White, L. Shaw, J. K. Lithgow and S. J. Foster, *J Bacteriol*, 2002, **184**, 5457–5467.

5 K. M. Orritt, L. Feng, J. F. Newell, J. N. Sutton, S. Grossman, T. Germe, L. R. Abbott, H. L. Jackson, B. K. L. Bury, A. Maxwell, M. J. McPhillie and C. W. G. Fishwick, *RSC Med Chem*, 2022, **13**, 831–839.

6 CSLI, *Methods for Dilution Antimicrobial Susceptibility Tests for Bacteria That Grow Aerobically 11th Edition CLSI Document M07-A11*, 2018.

7 H. Lin, C. Dai, T. F. Jamison and K. F. Jensen, *Angewandte Chemie International Edition*, 2017, **56**, 8870–8873.

8 S. K. Amundsen, T. Spicer, A. C. Karabulut, L. M. Londoño, C. Eberhart, V. Fernandez Vega, T. D. Bannister, P. Hodder and G. R. Smith, *ACS Chem Biol*, 2012, **7**, 879–891.

9 C. S. Q. Lim, K. P. Ha, R. S. Clarke, L.-A. Gavin, D. T. Cook, J. A. Hutton, C. L. Sutherell, A. M. Edwards, L. E. Evans, E. W. Tate and T. Lanyon-Hogg, *Bioorg Med Chem*, 2019, **27**, 114962.

10 WO 2006094840, A1, 2006.

11 WO2011088192 A1, 2011.

12 EP1724268 A1, 2006.

13 Y. Zhang, Q. Hou, X. Li, J. Zhu, W. Wang, B. Li, L. Zhao and H. Xia, *Eur J Med Chem*, 2019, **178**, 417–432.

14 WO2004087680 A1, 2004.

15 S. Leyva and H. Hernández, *J Fluor Chem*, 2010, **131**, 982–988.

16 WO2021116451 A1, 2021.

17 V. Venepally, R. B. N. Prasad, Y. Poornachandra, C. G. Kumar and R. C. R. Jala, *Bioorg Med Chem Lett*, 2016, **26**, 613–617.

18 J. Sheu, Y. Chen, K. Fang, T. Wang, C. Tzeng and C. Peng, *J Heterocycl Chem*, 1998, **35**, 955–964.
